# Synthesis and Psychotropic Properties of Novel Condensed Triazines for Drug Discovery

**DOI:** 10.3390/ph17070829

**Published:** 2024-06-25

**Authors:** Ervand G. Paronikyan, Shushanik Sh. Dashyan, Suren S. Mamyan, Ruzanna G. Paronikyan, Ivetta M. Nazaryan, Kristine V. Balyan, Hrachik V. Gasparyan, Sona A. Buloyan, Lernik S. Hunanyan, Nina G. Hobosyan

**Affiliations:** 1 Scientific Technological Center of Organic and Pharmaceutical Chemistry of National Academy of Sciences of Republic of Armenia, Ave. Azatutyan 26, Yerevan 0014, Armenia; ervand.paronikyan@mail.ru (E.G.P.); suren.mamyan@gmail.com (S.S.M.); paronikyan.ruzanna@mail.ru (R.G.P.); ivettanazaryan@gmail.com (I.M.N.); balyan-79@inbox.ru (K.V.B.); hrachikgasparyan@mail.ru (H.V.G.); lernik.hunanyan@rau.am (L.S.H.); ninahobosyan@gmail.com (N.G.H.); 2 Pharmacy Faculty, Haybusak University of Yerevan, 6 Abelyan St., Yerevan 0038, Armenia

**Keywords:** new heterocyclic system, [1,2,4]triazolo[4,3-*a*]pyridines, thieno[3,2-*d*][1,2,3]triazin-ones, neurotrophic activity, docking analysis, histopathology, morphometry

## Abstract

The exploration of heterocyclic compounds and their fused analogs, featuring key pharmacophore fragments like pyridine, thiophene, pyrimidine, and triazine rings, is pivotal in medicinal chemistry. These compounds possess a wide array of biological activities, making them an intriguing area of study. The quest for new neurotropic drugs among derivatives of these heterocycles with pharmacophore groups remains a significant research challenge. The aim of this research work was to develop a synthesis method for new heterocyclic compounds, evaluate their neurotropic and neuroprotective activities, study histological changes, and perform docking analysis. Classical organic synthesis methods were used in the creation of novel heterocyclic systems containing pharmacophore rings. To evaluate the neurotropic activity of these synthesized compounds, a range of biological assays were employed. Docking analysis was conducted using various software packages and methodologies. The neuroprotective activity of compound **13** was tested in seizures with and without pentylenetetrazole (PTZ) administration. Histopathological examinations were performed in different experimental groups in the hippocampus and the entorhinal cortex. As a result of chemical reactions, 16 new, tetra- and pentacyclic heterocyclic compounds were obtained. The biologically studied compounds exhibited protection against PTZ seizures as well as some psychotropic effects. The biological assays evidenced that 13 of the 16 studied compounds showed a high anticonvulsant activity by antagonism with PTZ. The toxicity of the compounds was low. According to the results of the study of psychotropic activity, it was found that the selected compounds have a sedative effect, except compound **13**, which exhibited activating behavior and antianxiety effects (especially compound **13**). The studied compounds exhibited antidepressant effects, especially compound **13**, which is similar to diazepam. Histopathological examination showed that compound **13** produced moderate changes in the brain and exhibited neuroprotective effects in the entorhinal cortex against PTZ-induced damage, reducing gliosis and neuronal loss. Docking studies revealed that out of 16 compounds, 3 compounds bound to the γ-aminobutyric acid type A (GABA_A_) receptor. Thus, the selected compounds demonstrated anticonvulsant, sedative, and activating behavior, and at the same time exhibited antianxiety and antidepressant effects. Compound **13** bound to the GABA_A_ receptor and exhibited antianxiety, antidepressant, and neuroprotective effects in the entorhinal cortex against PTZ-induced changes.

## 1. Introduction

The synthesis of pharmaceuticals aimed at treating neuropsychiatric disorders, notably epilepsy, poses a significant challenge for the field of synthetic organic chemistry [1]. Epilepsy is a common neurological disorder and affects around 65 million people worldwide. Epilepsy can be deadly as epileptic patients are prone to depression, injuries, and accidents [2]. Usually, epilepsies have a complex genetic basis where defects of the genes contribute to the altered cellular excitability [3]. Nevertheless, almost 70% of patients who were diagnosed with epilepsy can be successfully cured by antiepileptic drugs (AEDs) [4]. The majority of antiepileptic agents employed in medicine often induce toxic side effects in various organs and systems [5]. Moreover, one-third of affected individuals have drug-resistant epilepsy and continue to experience spontaneous recurrent seizures. These patients not only have continuous seizure activity but are also at great risk of developing cognitive impairment and comorbid mental health problems [6,7]. Consequently, there is considerable interest in researching and exploring anticonvulsants with combined psychotropic properties within a new series of compounds.

The designing, synthesizing, and evaluating of nitrogen-containing heterocyclic analogs with promising therapeutic properties remains a primary focus for researchers in the fields of organic and medicinal chemistry [8]. The presence of multiple pharmacophore groups in such compounds often amplifies their biological effects by fostering additional interactions with active receptor sites, potentially unlocking novel therapeutic pathways.

In pharmaceutical chemistry, triazines occupy a unique role, serving as a foundational framework for designing molecules with diverse biological applications. These applications include antibacterial [9], antimicrobial and antitubercular [10,11], anticancer [12,13,14,15], antimalarial [16,17], antiviral [18], antidepressant and anxiolytic [19,20,21], and more. Triazines exist in three distinct isomeric forms—1,2,3-, 1,2,4-, and 1,3,5-triazine isomers—owing to variations in the positions of nitrogen atoms within the ring structure. Among these, the 1,2,3-triazine isomer is relatively less explored due to its inherent instability compared to the other isomers. Consequently, synthetic pathways for this particular isomer are also constrained [22].

On the other hand, numerous studies have shown that condensed nitrogen-containing heterocyclic systems display a wide range of biological activities [23,24,25,26,27,28]. For example, the condensed pyrimidine ring is a fundamental component of various essential molecules in biology, including riboflavin (vitamin B2), folic acid (vitamin B9), DNA, and RNA. These molecules play crucial roles in cellular processes, such as energy metabolism, DNA replication, and protein synthesis [23,28]. The presence of the triazolopyrimidine and triazolopyridine moiety in several marketed drugs like Trapidil, Trazodone, Essramycin, Pyroxsulam, Flumetsulam, and Cevipabulin underscores its potential in medicinal chemistry, especially when combined with diverse functional groups [24,25]. Moreover, in our previous research, we reported that condensed systems, namely pyrano[3,4-c][1,2,4]triazolo[4,3-a]pyridines and pyrano[4″,3″:4′,5′]pyrido[3′,2′:4,5]thieno[3,2-d]pyrimidines, demonstrated high neurotropic activity [26,27].

Expanding upon the aforementioned considerations, our proposed research aims to develop synthesis methods for novel condensed heterocyclic compounds containing pharmacophore rings such as pyrano, pyridine, triazolo, thiophene, and triazine. Our objectives also include assessing their psychotropic activity and investigating the impact of compound structure on biological activity. Additionally, we aim to elucidate potential mechanisms of action and examine morphological changes in the brain tissue of animals exposed to these compounds.

## 2. Results and Discussions

### 2.1. Chemistry

An effective method for obtaining derivatives of tetracyclic pyrano[3,4-*c*]thieno[3,2-*e*][1,2,4]triazolo[4,3-*a*]pyridines (**2**–**9**) based on conversion of 5-mercapto-8,8-dimethyl-7,10-dihydro-8*H*-pyrano[3,4-*c*][1,2,4]triazolo[4,3-*a*]pyridine-6-carbonitrile (**1**) [29] was developed. Synthesis of compounds 2–4 was previously reported in our published articles [30,31]. However, in this work, we optimized the synthetic method for compounds **2**–**4**, increasing the yields to 92–95% and confirming their structures. Thus, the reaction was carried out by reacting compound 5-mercapto-8,8-dimethyl-7,10-dihydro-8*H*-pyrano[3,4-c][1,2,4]triazolo[4,3-*a*]pyridine-6-carbonitrile (**1**) with derivatives of chloroacetic amide containing an active methylene group. This reaction took place in the presence of sodium acetate in dry ethanol at a temperature of 60 °C.

**Scheme 1:**
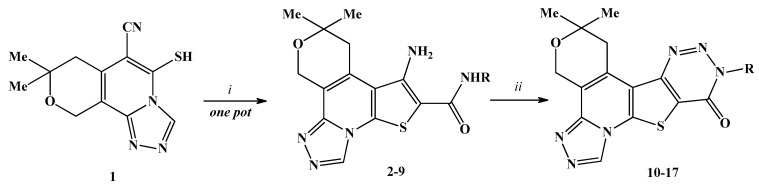
Synthesis of tetracyclic (compounds **2**–**9**) and pentacyclic (compounds **10**–**17**) heterocyclic compounds. (*i*) ClCH_2_CONHR, AcONa, dry EtOH, 2 h, 60 °C; (*ii*) DMF, MeCOOH, HCl, NaNO_2_/H_2_O, 3 h, 22 °C.

As a result of the *one-pot* reaction, the closure of the thiophene ring proceeds simultaneously with the alkylation, leading to the formation of tetracyclic pyrano[3,4-*c*]thieno[3,2-*e*][1,2,4]triazolo[4,3-*a*]pyridines (**2**–**9**) in high yields (81.8–95.0%) (Scheme 1, Table 1).

The structures of newly synthesized compounds **5**–**9** were confirmed by NMR, IR spectroscopy, and elemental analysis. Thus, in the ^1^H NMR spectra of these new compounds, the presence of the NH_2_ group proton at 6.78−7.01 ppm was observed. The IR spectra of **5**–**9** show amino group (NH, NH_2_) absorptions near 3111−3510 cm^−1^ and carboxyl group (C=O) absorptions near 1610−1673 cm^−1^ (see Supplementary Materials).

The synthesized new tetracyclic pyrano[3,4-*c*]thieno[3,2-*e*][1,2,4]triazolo[4,3-*a*]pyridines (**2**–**9**) still contain amino and amide functional groups, which can undergo new cyclization reactions. Thus, a method was developed based on compounds **2**–**9** for the synthesis of a new pentacyclic heterocyclic system—pyrano[4″,3″:4′,5′][1,2,4]triazolo[4″,3″:1′,6′]pyrido[3′,2′:4,5]thieno[3,2-*d*][1,2,3]triazin-11-ones (**10**–**17**) (Scheme 1, Table 1).

The structure of newly synthesized pyrano[4″,3″:4′,5′][1,2,4]triazolo[4″,3″:1′,6′]pyrido[3′,2′:4,5]thieno[3,2-*d*][1,2,3]triazin-11-ones **10**–**17** was supported by NMR and IR spectroscopy. Thus, in the ^1^H NMR spectra, the signals of NH and NH_2_ groups characteristic for the initial compounds **2**–**9** were absent, which indicated the cyclization of compounds **10**–**17**. The structure of compounds **10**–**17** was also supported by ^13^C NMR data. In ^13^C NMR spectra, the signals of CO groups of the triazine cycle are observed at 151.46–159.63 ppm. The IR spectra of compounds **10**–**17** did not show the characteristic bands of the amino group but showed the bands in the range of ν 1682–1708 cm^−1^ typical for the carbonyl group (see Supplementary Materials).

### 2.2. Biological Assays

The neurotropic activity of 16 newly synthesized heterocyclic compounds—tetracyclic pyrano[3,4-*c*]thieno[3,2-*e*][1,2,4]triazolo[4,3-*a*]pyridines (**2**–**9**) and pentacyclic pyrano[4″,3″:4′,5′][1,2,4]triazolo[4″,3″:1′,6′]pyrido[3′,2′:4,5]thieno[3,2-*d*][1,2,3]triazin-11-ones (**10**–**17**)—was determined according to indicators characterizing anticonvulsant, sedative, antianxiety activity, and side effects.

The anticonvulsive action of the tested compounds was assessed by evaluating the antagonism between the convulsive pentylenetetrazole (PTZ), thiosemicarbazide (TSC), camphor action, and maximal electroshock seizures (MESs) [32,33,34,35]. The PTZ-induced test is considered an experimental model for the clonic component of epilepsy seizures and prognostic anxiolytic [36] activities of the compounds. The MES test is used as an animal model for the generalized tonic seizures of epilepsy [37,38,39]. Ethosuximide and diazepam were used as controls [40]. The side effects of the compounds—neurotoxicity (movement coordination disorder, myorelaxation, and ataxia—were also studied in mice using the “rotating rod” test [33,41] and maximum tolerated dose (MTD). All studied compounds were tested in the model of TSC seizures (affecting GABA metabolism). To determine the 50% effective (ED_50_, causing the anticonvulsant effect in 50% of animals, which is calculated by testing PTZ antagonism) and 50% neurotoxic (TD_50_, causing myorelaxid effect in 50% of animals) doses, a statistical method of probit analysis by Litchfield and Wilcoxon was used [42,43]. From a practical point of view, the active compounds’ protective (TI = LD_50_/ED_50_) index was identified. All experiments were conducted eight times for each statistical analysis (n = 8).

The evaluation of the anticonvulsant activity of all the synthesized compounds revealed that they, to varying degrees, exhibit a PTZ antagonism. Thus, at a dose of 50 mg/kg, the compounds prevented PTZ clonic seizures in 50–80% of animals. Compounds **2**–**4** exhibited a lower efficacy, preventing PTZ seizures in only 20–30% of the animals. This level of efficacy was deemed insufficient, leading to the discontinuation of further investigation into these compounds. However, compounds **5**–**17** had a pronounced anticonvulsant action. Intraperitoneal injections of these compounds in mice, starting with a dose of 20 mg/kg, were accompanied by the prevention of PTZ seizures, and the ED_50_ ranged from 20 mg/kg to 55 mg/kg (Table 2).

It should be mentioned that tested compounds are more active than ethosuximide according to the test on PTZ but less than diazepam. The effective dose of ethosuximide (ED_50_, mg/kg) in the antagonism with PTZ in mice was 155.0 mg/kg, while for diazepam it was 0.5 mg/kg (Table 2).

The compounds were tested by the “rotating rod” method in a dose of 200 mg/kg in mice. The results showed that the compounds did not violate the coordination of movements; no signs of muscle relaxation were observed. The MTDs for the examined compounds are >500–1000 mg/kg (Table 2). Ethosuximide in the studied doses of 100–200 mg/kg in mice also did not cause muscle relaxation. The tested compounds are low-toxic and acute daily toxicity is in the range of over 500–900 mg/kg. Their therapeutic indexes (TIs) are much greater than that of the reference drug ethosuximide (Table 2), especially for compounds **13** and **14**. The compounds at the 100 mg/kg dose increased the latency of thiosemicarbazide seizures to 1.2–2.0 times compared with the control. Ethosuximide has approximately the same effect and an increase in the latent time of TSC convulsions was observed. Diazepam was not effective in this model.

The structure–activity relationship study revealed that all synthesized compounds exhibited anticonvulsant activity; however, the presence of a condensed triazine ring was found to be beneficial for enhancing this activity.

According to the MES test, the compounds studied, as well as the reference drugs, did not exhibit an anticonvulsant effect. They exhibit a weak effect by camphor antagonism (20–40% effectivity).

The most effective 13 compounds, **5**–**17**, were studied on the “open field”, “elevated plus maze” (EPM), and “forced swimming” tests, and on the model of “electroshock retrograde amnesia” at a dose of 50 mg/kg since the ED_50_ of these compounds are within 50 mg/kg of the confidence intervals.

**Figure 1:**
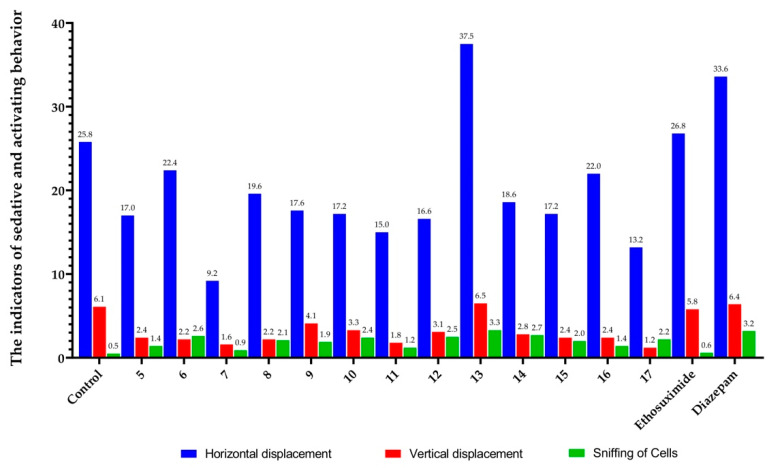
Graphical representation of the activity of the control group: compounds **5**–**17**, ethosuximide, and diazepam in the “open field” model.

In the “open field” behavioral model [44,45,46] in rats of the control group, the numbers of horizontal and vertical displacements and the number of examined cells were 25.8, 6.1, and 0.5, respectively (Table 3, Figure 1). The compounds under study caused changes in the behavioral indices in comparison with the control group; with the injection of the compounds, marked changes in the horizontal and vertical movements of animals were observed. Compounds **5**, **7**, **8**, **9**, **10**, **11**, **12**, **14**, **15**, and **17** statistically significantly decreased the horizontal movements of animals; the others did not result in any behavior changes associated with horizontal movements (compounds **6** and **16**). Only compound **13** increased horizontal movements, as in diazepam. As for vertical movements, all compounds statistically significantly reduced them, exhibiting some sedative effect, except for compound **13**, which increased vertical movements, thereby leading to activation of behavior.

However, all compounds found to be statistically significant compared with the control group increased the number of sniffing cell examinations, which may be due to the manifestation of the antianxiety activity of the compounds (Table 3), which was especially pronounced in compounds **6**, **12**, **13**, and **14**. Ethosuximide at the effective dose of 200 mg/kg had no effect on all indicators of research activities, while diazepam (2 mg/kg), in comparison with the control group of mice, caused a significant increase in the number of cells examined, i.e., pronounced antianxiety effect. In fact, compound **13**, like diazepam, exhibited activating behavior and the same anxiolytic effect.

The structure–activity relationship study revealed that the presence of a phenethyl radical substituted in the triazine ring (as seen in compound **13**) increased the horizontal movements of animals, exhibiting an activating effect similar to diazepam. All other compounds statistically decreased both horizontal and vertical movements, demonstrating a sedative effect. Antianxiety activity was observed in all synthesized compounds, with particularly notable effects observed in compounds containing a triazine ring, exemplified by compounds **12**, **13**, and **14**.

In order to assess the state of fear and despair in mice, the elevated plus maze (EPM) methodology developed by S. Pellow et al. (1986) was used [47,48,49]. The EPM is a behavioral assay (fear) used to estimate the antianxiety effect of pharmacological agents, synthetic compounds, etc. In brief, mice are placed at the junction of the four arms of the maze facing an open arm, which is followed by the recording of the entries/duration of the mice in each arm by a video-tracking system and an observer simultaneously for 5 min.

**Figure 2:**
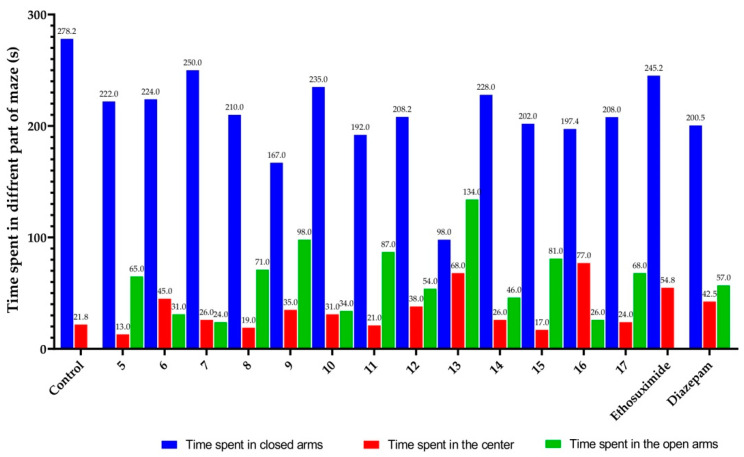
Graphical representation of the time mice spent in different parts of the maze after receiving compounds **5**–**17** and the compared drugs in the EPM model.

In the EPM model, the control animals remained predominantly in the closed arms of the maze (Table 4, Figure 2). After the introduction of all compounds, the time spent in closed arms and the number of entries into closed arms decreased statistically significantly. Compounds **6**, **9**, **10**, **12**, **13**, **14**, **16**, and **17** are statistically significant, compared with the control, which increased the time spent by experienced animals in the center; compounds **5**, **7**, **8**, **11**, and **15** either reduced or did not change this time.

After injection of the compounds, the experimental animals entered the open arms and remained there from 24 (compound **7**) to 134 (compound **13**) s in contrast to the control. At the same time, the control mice as well as mice who received ethosuximide at a dose of 200 mg/kg did not enter the open arms due to fear. Animals that received diazepam at a dose of 2 mg/kg also entered the open arms and stayed there for 57 s. The data obtained indicate the antianxiety activity of all compounds, which was especially expressed in compound **13**.

According to the structure–activity relationship study, the presence of a phenyl, phenethyl, or 4-ethoxyphenyl substituent on the nitrogen atom of the pentacyclic thieno[3,2-d][1,2,3]triazin-11-one moiety is favorable for antidepressant activity. However, the phenethyl functional group (as seen in compound **13**) exhibited enhanced activity, approximately twice as promising for antidepressant effects compared to diazepam.

The forced swimming test (FST, also known as Porsolt’s test) is one of the most commonly used assays [50,51,52]. The FST is used to monitor depressive-like behavior and is based on the assumption that immobility reflects a measure of behavioral despair.

**Figure 3:**
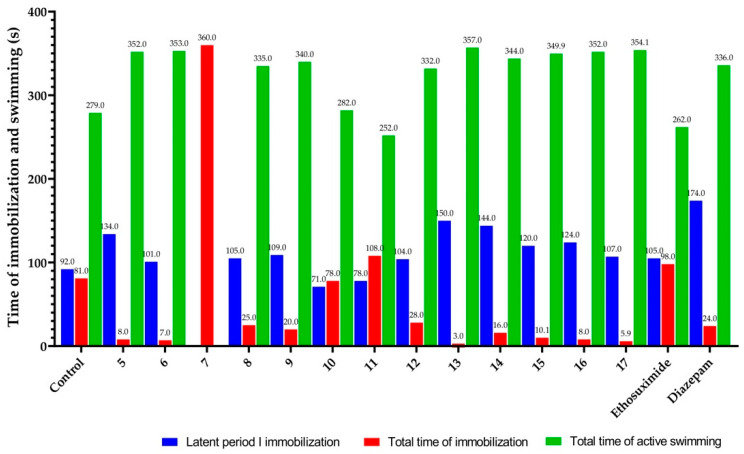
Graphical representation of the effects of compounds **5**–**17** and reference preparations in the “forced swimming” model.

In the “forced swimming” model (Table 5, Figure 3) in the control mice, the first immobilization occurs after 92 s. Many of the studied compounds (**5**, **6**, **9**, **12**, **13**, **14**, **15**, **16**, and **17**) tested at a dose of 50 mg/kg statistically significantly increased the latent period of the first immobilization and decreased the total time immobilization. Under the influence of compound **7**, immobilization was completely observed. Under the influence of compounds **10** and **11**, an increase in immobilization time was observed.

The total time of immobilization increased for many compounds (**5**, **6**, **8**, **9**, **11**, **12**, **14**, **15**, **16**, and **17**), and the greatest value was 357 s for compound **13** (Table 5). These compounds and diazepam increased the total time of active swimming. This suggests that the specified compounds studied at a dose of 50 mg/kg show antidepressant effects in the same manner as diazepam. The data obtained with the use of ethosuximide at a dose of 200 mg/kg coincide with the control data.

According to the structure–activity relationship study, the additional condensed triazine ring is favorable for antidepressant activity. However, the phenethyl functional group (as seen in compound **13**) substituted on the nitrogen atom of the triazine ring exhibits superior antidepressant activity compared to all the studied compounds and reference drugs.

For the evaluation of learning and memory, all the synthesized compounds were studied in a rodent model of CNS disorders. In the model of electroshock retrograde amnesia [43,53,54], which consisted of assessing conditioned passive avoidance reaction (CPAR) (Table 5) for 6 min, the control rats on the 1st and 2nd days of the experiment were in the light compartment for almost the entire period (280.0 s) (Table 6). Under conditions of retrograde amnesia in rats, the administration of compounds **5**, **8**, **12**, **13**, **14**, **15**, and **17** at a dose of 50 mg/kg every other day contributed to a slight increase in the time of reflex reproduction in animals. These values are statistically and significantly different from the control values and indicate the antiamnestic effect of the compounds. Similar data were obtained for the nootropic drug piracetam at a dose of 1000 mg/kg [55], in contrast to diazepam, which did not have an antiamnestic effect (under the same conditions, diazepam even leads to a decrease in behavioral indicators compared to the control) (Table 6).

**Figure 4:**
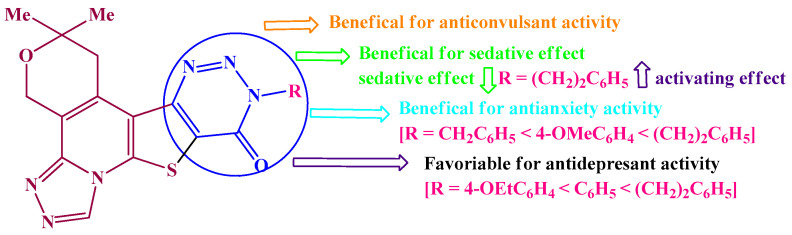
Summarization of the structure–activity relationship (SAR) analysis for the pentacyclic thieno[3,2-*d*][1,2,3]triazin-11-ones.

Summarizing the structure–activity relationship (SAR) study, the incorporation of an additional condensed triazine ring was found to enhance anticonvulsant, sedative antianxiety, and antidepressant activities across various biological tests. However, the functional group substituted on the nitrogen atom of the triazine ring can change the level of activity (Figure 4).

### 2.3. Histopathological Observation

To study the neuroprotective activity of compound **13**, we modeled experiments with prolonged exposure to this compound. After three days, half of the animals were treated with pentylenetetrazole (PTZ). The convulsant effects of PTZ are attributed to the inhibition of chloride channels in the GABA_A_–receptor complex, impairing the GABAergic inhibitory mechanisms in neurons [56,57]. Repeated injections of PTZ gradually induce seizures and lead to significant neuron loss in the CA1 and CA3 regions of the hippocampus [58,59]. Our observations align with these findings, indicating that the main regions undergoing pathological alterations after PTZ treatment are the hippocampus and entorhinal cortex, confirming the severity of PTZ’s impact on these critical areas of the brain. Our observations align with these findings, indicating that the main regions undergoing pathological alterations after PTZ treatment are the hippocampus and entorhinal cortex, confirming the severity of PTZ’s impact on these critical areas of the brain [60].

Consequently, we studied histological changes in these regions in various experimental groups.

**Figure 5:**
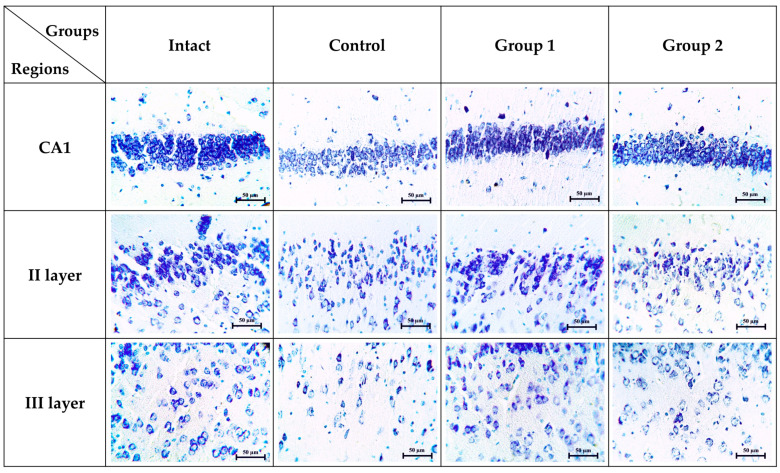
The results of the histological examination of the hippocampus CA1 region and II and III layers of the entorhinal cortex in various experimental groups: intact group—normal animals; control group—PTZ-treated animals; group 1—animals treated with compound **13**; and group 2—animals treated with PTZ and compound **13**. Magnification of ×400 (Nissl stain).

The hippocampi of the intact animals displayed a normal morphological appearance. Specifically, pyramidal cells in the CA1 region were neatly arranged with clear nuclei and visible nucleoli, and the cytoplasm showed a normal distribution of Nissl bodies (Figure 5).

**Figure 6:**
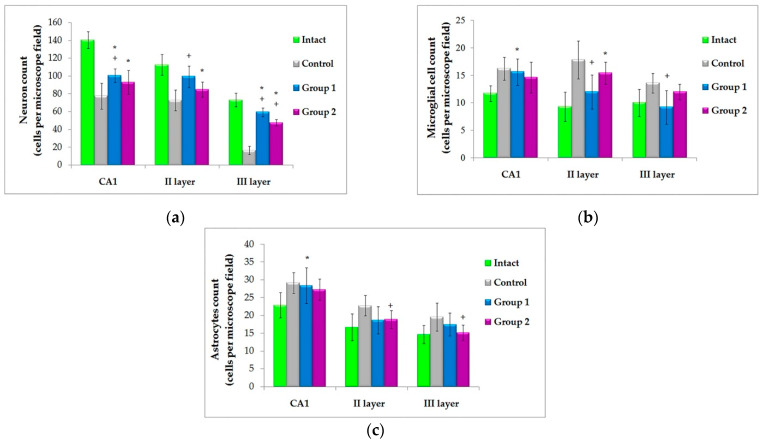
Morphometric analysis of hippocampus CA1 region and II and III layers of the entorhinal cortex in the intact group—normal animals; control group—PTZ-treated animals; group 1—animals treated with compound **13**; and group 2—animals treated with PTZ and compound **13**. (**a**) The count of neuronal cells; (**b**) the count of microglial cells; and (**c**) the count of astrocytes. Data are expressed as mean ± SD. * *p* < 0.05 compared to the intact group; + *p* < 0.05 compared to the pentylenetetrazole-treated group.

The average neuron count in the CA1 region was found to be 140.40 ± 9.3 cells per microscopic field (Figure 6a). In this region, microglial cells were sparse, totaling only 11.70 ± 1.418 cells, whereas astrocytes, constituting the majority of the glial cells, numbered 22.90 ± 3.510 cells (Figure 6b,c). Histological examination of the intact entorhinal cortex revealed that layer I contained few cells, while layer II was characterized by clusters of round, hyperchromatic neurons with prominent nucleoli (Figure 5). Morphometric analysis determined the neuron count in layer II to be 112.7 ± 11.767 cells per microscopic field (Figure 6a). Layer III contained medium-sized pyramidal cells with clear oval nuclei (Figure 5), numbering 73.30 ± 7.689 cells per microscopic field (Figure 6a). Across all layers, glial cells were present in normal quantities. Specifically, the count of microglial cells in layers II and III was 9.30 ± 2.669 and 10.00 ± 2.494 cells per microscopic field, respectively. The counts of astrocytes were 16.70 ± 3.743 and 14.70 ± 2.497 cells in these layers (Figure 6b,c).

Compared to the intact group, animals treated with PTZ exhibited significant neuronal chromatolysis across all regions of the hippocampus. Notably, neurons were largely absent, and the CA1 region specifically showed signs of sclerosis (Figure 5). The count of pyramidal cells was dramatically reduced to 77.4 ± 14.5 cells per microscopic field (Figure 6a), indicating a profound impact of PTZ treatment. Furthermore, there was a notable activation of glial cells, with gliosis being a characteristic feature of epilepsy [61]. Activated microglia and astrocytes in seizures cause inflammation in brain tissue by releasing a number of proinflammatory mediators [62].

The microglial and astroglial cell counts in the hippocampus increased to 16.20 ± 2.044 and 29.20 ± 2.936 cells per microscopic field, respectively (Figure 6b,c), compared to the norm. Similarly, the entorhinal cortex showed significant neuronal cell destruction, with neurons in all layers undergoing chromatolysis and becoming scarce (Figure 5). In layers II and III, the number of pyramidal neurons markedly decreased to 72.7 ± 11.7 and 16.6 ± 4.835 cells per microscopic field, respectively (Figure 6a). Correspondingly, glial cell counts in these layers of the cortex were elevated, with microglia numbers reaching 17.80 ± 3.425 and 13.60 ± 1.776 cells per microscopic field in layers II and III, respectively (Figure 6b). The count of astrocytes in these layers increased to 22.80 ± 2.860 and 19.60 ± 3.921 cells per microscopic field (Figure 6c).

The hippocampi of animals from experimental group 1 showed similarities to those of the intact animals. In the CA1 region, neurons were mostly neatly arranged with clear nuclei and visible nucleoli, and only a few exhibited chromatolysis of Nissl bodies. Morphometric analysis revealed that the count of pyramidal cells in this group (100.40 ± 7.633 cells) was significantly lower compared to the intact animals, yet significantly higher than in the pentylenetetrazole-treated group (*p* < 0.05). Similarly, the counts of microglial cells (15.60 ± 2.413 cells) and astrocytes (28.40 ± 4.993 cells) were also significantly higher compared to the intact group (*p* < 0.05) (Figure 6b,c).

In group 1, the morphological characteristics of the cortex closely resembled those observed in the intact animals (Figure 5). Although the number of neuronal cells in layer II was slightly reduced (99.40 ± 12.094 cells) compared to the intact group, it was significantly higher than that in the pentylenetetrazole-treated group (*p* < 0.05). In layer III, the number of pyramidal cells was the highest among all experimental groups, with 59.50 ± 4.882 cells per microscopic field (Figure 6a). The counts of microglial cells in these layers did not differ from those in intact animals (12.00 ± 3.091 and 9.20 ± 3.048 cells per microscopic field, respectively) but were significantly lower compared to the PTZ-treated group (*p* < 0.05) (Figure 6b). The only increase was observed in the count of astrocytes compared to intact animals (18.70 ± 3.802 and 17.50 ± 3.206 cells per microscopic field, respectively) (Figure 6b). Overall, pathological processes were moderate in this group compared with other experimental groups.

In the hippocampi of animals from group 2, compared with the control group, moderate changes were observed in the hippocampus, including mild gliosis. The number of pyramidal cells in the CA1 region did not show a significant reduction, though some nerve cells exhibited chromatolysis of Nissl bodies (Figure 5). The neuron count was 93 ± 84.7 cells per microscopic field, which was not statistically different from the control group but was statistically lower than that in the intact animals (Figure 6a). The microglial count was 14.60 ± 2.797 cells per microscopic field, which did not statistically differ from both the intact and control groups (Figure 6b,c). This group also demonstrated a reduction in the number of nerve cells in the entorhinal cortex. However, neurons in layer II were relatively well preserved (Figure 5), numbering 84.70 ± 8.525 cells per microscopic field. The pyramidal cells in layer III were counted at 47.7 ± 3.713 per microscopic field, which was significantly higher than those in the PTZ-treated group (*p* < 0.05) (Figure 6a).

Gliosis was also moderate, with the number of microglial cells not significantly different from the PTZ-treated group, totaling 15.4 ± 2.011 and 12 ± 1.414 cells, respectively (Figure 6b). The count of astrocytes was significantly lower compared to the control animals, with 18.9 ± 2.47 and 15.2 ± 2.098 cells, respectively (*p* < 0.05) (Figure 6c).

Thus, compound **13** exhibited a neuroprotective effect in the entorhinal cortex against PTZ-induced changes, while its effect in the hippocampus was less pronounced.

### 2.4. Molecular Docking

The AEDs act by lowering the electrical activity of neurons, either through the depolarization of action potentials or through post-synaptic inhibition of impulse generation [63]. These effects are achieved by acting on voltage-gated ion channels and gamma-aminobutyric acid (GABA) receptors, respectively [64]. Due to that, docking analysis has been performed to indicate the interaction of synthesized compounds with GABA receptors and give us the possible mechanisms of interaction.

#### Docking and Conformational Analysis of GABA_A_ Receptor Complexation

Docking analysis of the studied compounds revealed that from 16 compounds, the interaction of 3 (compounds **7**, **8**, and **13**) with the GABA_A_ receptor was observed. This type of receptor is ionotropic: when GABA binds to the receptor, an ion channel opens in the membrane of the nerve cell, allowing chloride ions to rush into the cell. This reduces the cell’s reactivity, which is crucial for its psychotropic activity. Hence, we specifically targeted this type of GABA receptor.

**Figure 7:**
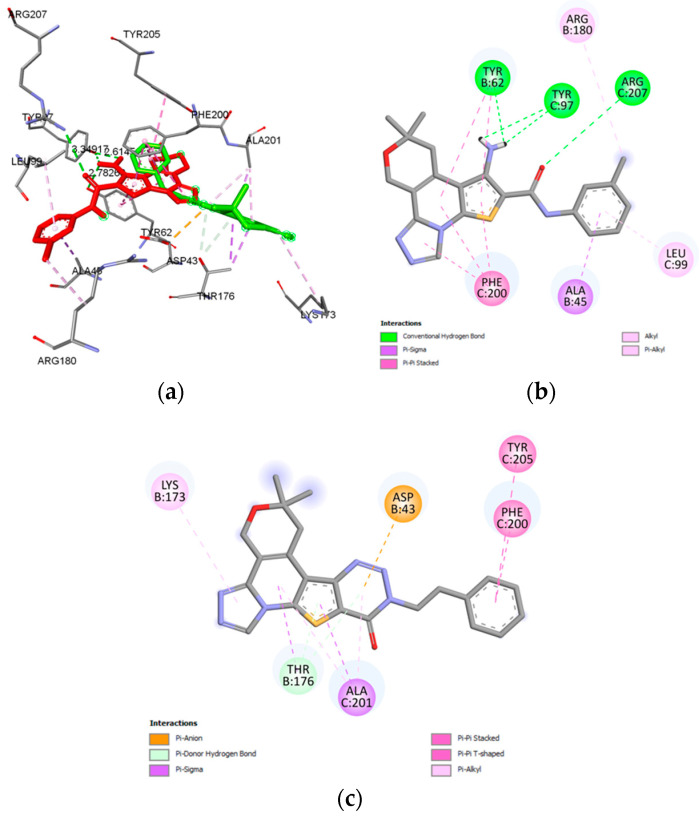
The superpositions and conformational maps of compounds **8** and **13**. The superpositions of compounds **8** and **13** (**a**); the conformational map of compound **8** (**b**); and the conformational map of compound **13** (**c**).

In our previous article [26], we reported the results of molecular docking of diazepam with the GABA_A_ receptor. This analysis revealed interactions between diazepam and specific amino acid residues, including Phe200, Tyr205, Ala201, Tyr97, Glu155, Tyr202, Asp43, Tyr176, Tyr66, Asn41, Gln64, and Tyr62 within chains B and C. The positioning of the control compound and the engagement of certain amino acid residues in complexation suggest that compounds **8** and **13** interact with the same site as diazepam, namely the Extracellular Domain (ECD) of chains B and C, specifically at subsite 1 (benzamide site) [65]. In this case, the localization and type of binding differ from each other (Figure 7).

The steric parameters we obtained indicate that compounds **8** and **13** are mirror-oriented relative to the binding site and form an angle of 97.2° relative to each other (Figure 7a). Compound **8** exhibited three hydrogen bonds with amino acid residues Tyr62 of chain B, Tyr97, and Arg207 of chain C (Figure 7b). The maximum distance was observed in the NH_2_ group of Arg207 with a value of 3.3 Å at an interaction angle of 41.91°. In addition, hydrophobic interactions with Arg180, Leu99, Ala45, and Phe200 were also observed for compound **8** (Figure 7b). In contrast to compound **8**, compound **13** did not exhibit any hydrogen bonding. The interaction is facilitated by hydrophobic forces with amino acid residues Lys173, Tyr205, Phe200, and Ala201 (Figure 7c). Furthermore, an anionic bond with Asp43 of chain B was observed for compound **13**.

Thus, compared to compound **8**, diazepam did not form hydrogen bonds during complexation. At the same time, compound **13** showed a similar picture with diazepam, where hydrophobic interactions predominate.

**Figure 8:**
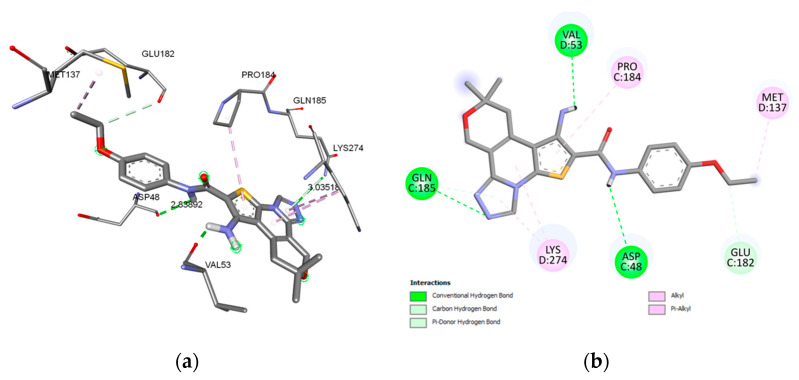
Spatial location and conformational map of compound **7**. The spatial location of compound **7** in GABA_A_ subsite 3 (**a**); the conformational map of compound **7** (**b**).

The binding site of compound **7** differs from those of the two previous compounds (**8** and **13**). Complexation occurs at subsite 3 of chains C and D (Figure 8a). This site is situated near the Transmembrane Domain (TMD) on the Extracellular Domain (ECD), where, in the literature, interactions with endocannabinoid signaling molecules such as anandamide are described [66]. Complexation of compound **7** occurred due to both hydrogen and hydrophobic forces.

Hydrogen bonds were observed with amino acid residues Asp48, Val53, and Gln185 with distances of 2.8 Å, 1.9 Å, and 3.0 Å, respectively. The hydrophobic type of binding occurred due to residues of Pro184, Met137, and Lys274 (Figure 8b).

We also calculated biophysical indicators for the complexation of compounds **7**, **8**, and **13** (Table 7).

The results indicate that the maximum binding constant was observed in compound **13**, with a value of 7.7 × 10^6^. In second place is compound **8**, whose binding constant is 5.3 × 10^6^. The smallest was compound **7** with a value of 9.4 × 10^5^.

Thus, compounds **8** and **13** exhibit different spatial localization within subsite 1. During complexation, compound **8** primarily engages in hydrogen bonding forces, while the interaction of compound **13** predominantly relies on hydrophobic forces. At the same time, only compound **13** exhibits an anionic bond with Asp43. The interaction of compound **7** is noteworthy; despite its low biophysical parameters, it binds to a specific site (subsite 3) where endocannabinoid analogs like anandamide are predominantly bound.

## 3. Materials and Methods

### 3.1. Chemistry

General Information: All chemicals and solvents were of commercially high purity grade and purchased from Sigma-Aldrich (Saint Louis, MO, USA). Melting points (m.ps.) were determined using a Boetius microtable and are expressed in degrees centigrade (°C). ^1^H NMR and ^13^C NMR spectra were recorded in DMSO-*d_6_*/CCl_4_, 1/3, *v*/*v* solution (300 MHz for ^1^H and 75.462 MHz for ^13^C) using a Varian mercury spectrometer (Varian Inc., Palo Alto, CA, USA). Chemical shifts are reported as δ (parts per million) relative to TMS (tetramethylsilane) as the internal standard. IR spectra were recorded using a Nicolet Avatar 330-FTIR spectrophotometer (Thermo Nicolet, Foster, CA, USA) and the reported wave numbers are given in cm^−1^. Mass spectra were recorded using XEVO G3 QTof spectrometers (Waters Corporation Company, Milford, MA, USA). Elemental analyses were performed using a Euro EA 3000 Elemental Analyzer (EuroVector, Pavia, Italy).

Compound **1** [29] was prepared by the method presented in the literature. Its spectroscopic data and physical and chemical properties were similar to that reported earlier.

i. General synthesis method of compounds **2**–**9**. A mixture containing compound **1** (1.3 g, 5 mmol) and its corresponding chloroacetic amide derivative (5 mmol) in dry ethanol (15 mL) was maintained at 60 °C for 6 h with anhydrous sodium acetate (1.23 g, 15 mmol) serving as a soft basic reagent. Following cooling of the reaction mixture, the resultant crystals were isolated via filtration, washed sequentially with water and ethanol, and subsequently recrystallized from ethanol.

7-Amino-9,9-dimethyl-8,11-dihydro-9*H*-pyrano[3,4-*c*]thieno[3,2-*e*][1,2,4]triazolo[4,3-*a*]pyridine-6-carboxamide (**2**)
White solid (1.49 g, 93%). All other physicochemical parameters were similar to the literature data [30].
7-Amino-9,9-dimethyl-*N*-phenyl-8,11-dihydro-9*H*-pyrano[3,4-*c*]thieno[3,2-*e*][1,2,4]triazolo[4,3-*a*]pyridine-6-carboxamide (**3**)
White solid (1.81 g, 92%). All other physicochemical parameters were similar to the literature data [31].
7-Amino-*N*-benzyl-9,9-dimethyl-8,11-dihydro-9*H*-pyrano[3,4-*c*]thieno[3,2-*e*][1,2,4]triazolo[4,3-*a*]pyridine-6-carboxamide (**4**)
White solid (1.94 g, 95%). All other physicochemical parameters were similar to the literature data [31].
7-Amino-9,9-dimethyl-*N*-(2-phenylethyl)-8,11-dihydro-9*H*-pyrano[3,4-*c*]thieno[3,2-*e*][1,2,4]triazolo[4,3-*a*]pyridine-6-carboxamide (**5**)
White solid (1.84 g, 87.1%), m.p.: 292–293 °C; IR ν/cm^−1^: 3201, 3275, 3458 (NH, NH_2_); 3090 (N=CH); 1610 (CO). ^1^H NMR (300 MHz, DMSO-*d_6_*/CCl_4_, 1/3) δ_H_ (ppm): 1.34 (s, 6H, C(Me)_2_), 2.85 (t, ***J*** = 7.6 Hz, 2H, NHCH_2_C*H_2_*), 3.14 (t, ***J*** = 1.8 Hz, 2H, 8-CH_2_), 3.40–3.49 (m, 2H, NHC*H_2_*), 4.95 (t, ***J*** = 1.8 Hz, 2H, 11-CH_2_), 6.78 (s, 2H, NH_2_), 7.12–7.30 (m, 5H, 5CH_Ar_), 7.76 (t, ***J*** = 5.6 Hz, 1H, NH)), 9.32 (s, 1H, 3-CH). ^13^C NMR (75.462 MHz, DMSO-*d_6_*/CCl_4_, 1/3) δ_C_: 26.05 (2Me), 35.33 (8-CH_2_), 35.42 (CH_2_), 40.49 (NHCH_2_), 69.45 (C^9^), 115.56 (C), 118.75 (C), 125.51 (CH_Ar_), 127.81 (2CH_Ar_), 128.27 (2CH_Ar_), 129.54 (C), 131.40 (C), 134.38 (CH), 139.23 (C), 145.50 (C), 149.01 (C), 164.19 (CO). Anal. calcd for C_22_H_23_N_5_O_2_S: C 62.69; H 5.50; N 16.61; S 7.61%. Found: C 62.75; H 5.54; N 16.53; S 7.56.
7-Amino-*N*-(4-methoxyphenyl)-9,9-dimethyl-8,11-dihydro-9*H*-pyrano[3,4-*c*]thieno[3,2-*e*][1,2,4]triazolo[4,3-*a*]pyridine-6-carboxamide (**6**)
White solid (1.89 g, 89.1%), m.p.: 280–281 °C; IR ν/cm^−1^: 3123, 3197, 3257, 3365, 3463 (NH, NH_2_); 3067 (N=CH); 1673 (CO). ^1^H NMR (300 MHz, DMSO-*d_6_*) δ_H_ (ppm): 1.31 (s, 6H, C(Me)_2_), 3.15 (t, ***J*** = 1.8 Hz, 2H, 8-CH_2_), 3.74 (s, 3H, OCH_3_), 4.95 (t, ***J*** = 1.8 Hz, 2H, 11-CH_2_), 6.87–6.94 (m, 2H, 2CH_Ar_), 6.95 (br. s, 2H, NH_2_), 7.50–7.57 (m, 2H, 2CH_Ar_), 9.54 (s, 1H, 3-CH), 9.57 (br. s, 1H, NH). ^13^C NMR (75.462 MHz, DMSO-*d_6_*) δ_C_: 26.19 (2Me), 35.58 (8-CH_2_), 55.14 (OCH_3_), 58.48 (11-CH_2_), 69.81 (C^9^), 95.39 (C), 113.59 (2CH_Ar_), 115.81 (C), 118.58 (C), 122.90 (2CH_Ar_), 129.67 (C), 131.60 (C), 132.42 (C), 135.30 (CH), 145.72 (C), 150.12 (C), 155.57 (C), 163.32 (CO). Anal. calcd for C_21_H_21_N_5_O_3_S: C 59.56; H 5.00; N 16.54; S 7.57%. Found: C 59.63; H 4.96; N 16.47; S 7.62.
7-Amino-*N*-(4-ethoxyphenyl)-9,9-dimethyl-8,11-dihydro-9*H*-pyrano[3,4-*c*]thieno[3,2-*e*][1,2,4]triazolo[4,3-*a*]pyridine-6-carboxamide (**7**)
White solid (2.0 g, 91.4%), m.p.: 299–300 °C; IR ν/cm^−1^: 3111, 3194, 3268, 3460 (NH, NH_2_); 3081 (N=CH); 1673 (CO). ^1^H NMR (300 MHz, DMSO-*d_6_*/CCl_4_, 1/3) δ_H_ (ppm): 1.36 (s, 6H, C(Me)_2_), 1.40 (t, ***J*** = 7.0 Hz, 3H, OCH_2_C*H_3_*), 3.14 (t, ***J*** = 1.8 Hz, 2H, 8-CH_2_), 4.00 (q, ***J*** = 7.0 Hz, 3H, OC*H_2_*CH_3_), 4.97 (t, ***J*** = 1.8 Hz, 2H, 11-CH_2_), 6.74–6.80 (m, 2H, 2CH_Ar_), 6.87 (s, 2H, NH_2_), 7.51–7.57 (m, 2H, 2CH_Ar_), 9.24 (br. s, 1H, NH), 9.26 (s, 1H, CH). ^13^C NMR (75.462 MHz, DMSO-*d_6_*/CCl_4_, 1/3) δ_C_: 14.42 (Me), 26.03 (2Me), 35.46 (8-CH_2_), 58.41 (11-CH_2_), 62.59 (OCH_2_), 69.36 (C^9^), 113.43 (2CH_Ar_), 115.75 (C), 118.49 (C), 122.37 (2CH_Ar_), 129.37 (C), 131.49 (C), 131.87 (C), 134.11 (CH), 145.50 (C), 149.50 (C), 154.51 (C), 162.78 (CO). Anal. calcd for C_22_H_23_N_5_O_3_S: C 60.39; H 5.30; N 16.01; S 7.33%. Found: C 60.32; H 5.34; N 16.12; S 7.25.
7-Amino-*N*-(3-chlorophenyl)-9,9-dimethyl-8,11-dihydro-9*H*-pyrano[3,4-*c*]thieno[3,2-*e*][1,2,4]triazolo[4,3-*a*]pyridine-6-carboxamide (**8**)
White solid (1.99 g, 93.0%), m.p.: 319–320 °C; IR ν/cm^−1^: 3181, 3258, 3329, 3510 (NH, NH_2_); 3105 (N=CH); 1629 (CO). ^1^H NMR (300 MHz, DMSO-*d_6_*/CCl_4_, 1/3) δ_H_ (ppm): 1.36 (s, 6H, C(Me)_2_), 3.14 (t, ***J*** = 1.8 Hz, 2H, 8-CH_2_), 4.97 (t, ***J*** = 1.8 Hz, 2H, 11-CH_2_), 6.96–7.02 (m, 1H, CH_Ar_), 7.01 (br. s, 2H, NH_2_), 7.18–7.26 (m, 1H, CH_Ar_), 7.59–7.65 (m, 1H, CH_Ar_), 7.88–7.91 (m, 1H, CH_Ar_), 9.30 (s, 1H, CH), 9.53 (br. s, 1H, NH). ^13^C NMR (75.462 MHz, DMSO-*d_6_*/CCl_4_, 1/3) δ_C_: 26.02 (2Me), 35.48 (8-CH_2_), 58.39 (11-CH_2_), 69.37 (C^9^), 94.41 (C), 115.86 (C), 118.26 (C), 118.50 (CH_Ar_), 120.22 (CH_Ar_), 122.25 (C), 128.79 (CH_Ar_), 129.37 (C), 132.37 (C), 132.84 (CH_Ar_), 134.23 (CH), 140.27 (C), 145.50 (C), 150.83 (C), 163.17 (CO). Anal. calcd for C_20_H_18_ClN_5_O_2_S: C 56.14; H 4.24; N 16.37; S 7.49%. Found: C 56.23; H 4.30; N 16.46; S 7.41.
7-Amino-*N*-(2-furylmethyl)-9,9-dimethyl-8,11-dihydro-9*H*-pyrano[3,4-*c*]thieno[3,2-*e*][1,2,4]triazolo[4,3-*a*]pyridine-6-carboxamide (**9**)
Pale yellow solid (1.63 g, 81.8%), m.p.: 279–280 °C; IR ν/cm^−1^: 3133, 3324, 3495 (NH, NH_2_, N=CH); 1615 (CO). ^1^H NMR (300 MHz, DMSO-*d_6_*) δ_H_ (ppm): 1.31 (s, 6H, C(Me)_2_), 3.14 (t, ***J*** = 1.8 Hz, 2H, 8-CH_2_), 4.41 (d, ***J*** = 5.6 Hz, 2H, NHC*H_2_*), 4.94 (t, ***J*** = 1.8 Hz, 2H, 11-CH_2_), 6.25 (dd, ***J*** = 3.2, 0.8 Hz, 1H, CH_furyl_), 6.39 (dd, ***J*** = 3.2, 1.8 Hz, 1H, CH_furyl_), 6.88 (s, 2H, NH_2_), 7.56 (dd, ***J*** = 1.8, 0.8 Hz, 1H, CH_furyl_), 8.41 (t, ***J*** = 5.6 Hz, 1H, NH), 9.52 (s, 1H, CH). ^13^C NMR (75.462 MHz, DMSO-*d_6_*) δ_C_: 26.17 (2Me), 35.34 (8-CH_2_), 35.62 (CH_2_), 58.46 (11-CH_2_), 69.78 (C^9^), 95.34 (C), 106.62 (CH), 110.37 (CH), 115.74 (C), 118.67 (C), 129.68 (CH), 132.08 (C), 135.30 (3-CH), 141.78 (C), 145.69 (C), 149.52 (C), 152.57 (C), 164.43 (CO). Anal. calcd for C_19_H_19_ClN_5_O_3_S: C 57.42; H 4.82; N 17.62; S 8.07%. Found: C 57.51; H 4.87; N 17.71; S 7.98.

ii. General synthesis method of compounds **10**–**17**. Compounds **2**–**9** (3 mmol) were added to a solution of the corresponding amino amide in a mixture of 3 mL of DMF, 4.5 mL of acetic acid, and 1.5 mL of hydrochloric acid; stirring was initiated at 20 °C. Subsequently, a solution of 0.6 g (9 mmol) of sodium nitrite in 3 mL of water was added, and stirring was continued for 3 h at 20 °C. The obtained precipitate was then separated by filtration, washed with water, dried, and subsequently recrystallized from a solvent mixture of chloroform and ethanol (2:1 *v*/*v*).

6,6-Dimethyl-6,7-dihydro-4*H*-pyrano[4″,3″:4′,5′][1,2,4]triazolo[4″,3″:1′,6′]pyrido[3′,2′:4,5]thieno[3,2-*d*][1,2,3]triazin-11(10*H*)-one (**10**)
White solid (0.85 g, 85.9%), m.p.: >360 °C; IR ν/cm^−1^: 3050, 3093 (NH, N=CH); 1708 (CO). ^1^H NMR (300 MHz, DMSO-*d_6_*/CCl_4_, 1/3) δ_H_ (ppm): 1.38 (s, 6H, C(Me)_2_), 3.38 (t, ***J*** = 1.8 Hz, 2H, 7-CH_2_), 5.03 (t, ***J*** = 1.8 Hz, 2H, 4-CH_2_), 9.90 (s, 1H, CH), 157.4 (br. s, 1H, NH). ^13^C NMR (75.462 MHz, DMSO-*d_6_*/CCl_4_, 1/3) δ_C_: 26.15 (2Me), 36.37 (7-CH_2_), 58.38 (4-CH_2_), 69.85 (C^6^), 118.82 (C), 119.88 (C), 129.53 (C), 136.26 (1-CH), 136.28 (C), 136.47 (C), 145.75 (C), 149.25 (C), 153.19 (CO). TOF MS ES+ [MH]^+^ *m*/*z* 329.0822 (calcd for C_14_H_12_N_6_O_2_S, 329.0821). Anal. calcd for C_14_H_12_N_6_O_2_S: C 51.21; H 3.68; N 25.59; S 9.77%. Found: C 51.13; H 3.75; N 25.50; S 9.83.
6,6-Dimethyl-10-phenyl-6,7-dihydro-4*H*-pyrano[4″,3″:4′,5′][1,2,4]triazolo[4″,3″:1′,6′]pyrido[3′,2′:4,5]thieno[3,2-*d*][1,2,3]triazin-11(10*H*)-one (**11**)
White solid (1.05 g, 86.6%), m.p.: 328–329 °C; IR ν/cm^−1^: 3065 (N=CH); 1685 (CO). ^1^H NMR (300 MHz, DMSO-*d_6_*) δ_H_ (ppm): 1.37 (s, 6H, C(Me)_2_), 3.36 (t, ***J*** = 1.8 Hz, 2H, 7-CH_2_), 5.03 (t, ***J*** = 1.8 Hz, 2H, 4-CH_2_), 7.57–7.69 (m, 3H, 3CH_Ar_), 7.71–7.76 (m, 2H, 2CH_Ar_), 9.93 (s, 1H, CH). ^13^C NMR (75.462 MHz, DMSO-*d_6_*) δ_C_: 26.11 (2Me), 36.26 (7-CH_2_), 58.45 (4-CH_2_), 69.92 (C^6^), 119.09 (C), 119.74 (C), 122.68 (C), 126.56 (2CH_Ar_), 129.02 (2CH_Ar_), 129.34 (C), 129.54 (CH_Ar_), 136.38 (1-CH), 136.78 (C), 138.04 (C), 145.72 (C), 148.04 (C), 152.26 (CO). Anal. calcd for C_20_H_16_N_6_O_2_S: C 59.39; H 3.99; N 20.78; S 7.93%. Found: C 59.46; H 4.05; N 20.70; S 7.86.
10-Benzyl-6,6-dimethyl-6,7-dihydro-4*H*-pyrano[4″,3″:4′,5′][1,2,4]triazolo[4″,3″:1′,6′]pyrido[3′,2′:4,5]thieno[3,2-*d*][1,2,3]triazin-11(10*H*)-one (**12**)
White solid (1.08 g, 86.2%), m.p.: 305–306 °C; IR ν/cm^−1^: 3111 (N=CH); 1676 (CO). ^1^H NMR (300 MHz, DMSO-*d_6_*/CCl_4_, 1/3) δ_H_ (ppm): 1.38 (s, 6H, C(Me)_2_), 3.33 (t, ***J*** = 1.8 Hz, 2H, 7-CH_2_), 5.00 (t, ***J*** = 1.8 Hz, 2H, 4-CH_2_), 5.67 (s, 2H, NCH_2_), 7.25–7.38 (m, 3H, 3CH_Ar_), 7.44–7.50 (m, 2H, 2CH_Ar_), 9.77 (s, 1H, CH). ^13^C NMR (75.462 MHz, DMSO-*d_6_*/CCl_4_, 1/3) δ_C_: 26.00 (2Me), 36.12 (7-CH_2_), 52.71 (NCH_2_), 58.29 (4-CH_2_), 69.44 (C^6^), 119.10 (C), 119.52 (C), 121.50 (C), 127.74 (CH_Ar_), 128.18 (2CH_Ar_), 128.30 (2CH_Ar_), 128.99 (C), 134.93 (C), 135.65 (1-CH), 136.43 (C), 148.39 (C), 148.63 (C), 151.64 (CO). TOF MS ES+ [MH]^+^ *m*/*z* 419.1293 (calcd for C_21_H_18_N_6_O_2_S, 419.1290). Anal. calcd for C_21_H_18_N_6_O_2_S: C 60.27; H 4.34; N 20.08; S 7.66%. Found: C 60.34; H 4.28; N 19.96; S 7.58.
6,6-Dimethyl-10-(2-phenylethyl)-6,7-dihydro-4*H*-pyrano[4″,3″:4′,5′][1,2,4]triazolo[4″,3″:1′,6′]pyrido[3′,2′:4,5]thieno[3,2-*d*][1,2,3]triazin-11(10*H*)-one (**13**)
White solid (1.14 g, 87.8%), m.p.: 333–334 °C; IR ν/cm^−1^: 3110 (N=CH); 1682 (CO). ^1^H NMR (300 MHz, DMSO-*d_6_*) δ_H_ (ppm): 1.33 (s, 6H, C(Me)_2_), 3.21 (t, ***J*** = 7.4 Hz, 2H, NCH_2_C*H_2_*), 3.25 (t, ***J*** = 1.8 Hz, 2H, 7-CH_2_), 4.71 (t, ***J*** = 7.4 Hz, 2H, NC*H_2_*CH_2_), 4.97 (t, ***J*** = 1.8 Hz, 2H, 4-CH_2_), 7.19–7.34 (m, 5H, 5CH_Ar_), 9.88 (s, 1H, CH). ^13^C NMR (75.462 MHz, DMSO-*d_6_*) δ_C_: 26.09 (2Me), 34.09 (CH_2_), 36.22 (7-CH_2_), 50.87 (NCH_2_), 58.37 (4-CH_2_), 69.83 (C^6^), 118.93 (C), 119.57 (C), 121.54 (C), 126.58 (CH_Ar_), 128.50 (2CH_Ar_), 128.68 (2CH_Ar_), 129.30 (C), 136.28 (1-CH), 136.59 (C), 137.58 (C), 145.67 (C), 148.67 (C), 152.20 (CO). Anal. calcd for C_22_H_20_N_6_O_2_S: C 61.09; H 4.66; N 19.43; S 7.41%. Found: C 61.16; H 4.70; N 19.21; S 7.29.
10-(4-Methoxyphenyl)-6,6-dimethyl-6,7-dihydro-4*H*-pyrano[4″,3″:4′,5′][1,2,4]triazolo[4″,3″:1′,6′]pyrido[3′,2′:4,5]thieno[3,2-*d*][1,2,3]triazin-11(10*H*)-one (**14**)
Yellow solid (1.24 g, 94.8%), m.p.: 222–223 °C; IR ν/cm^−1^: 3102 (N=CH); 1671 (CO). ^1^H NMR (300 MHz, DMSO-*d_6_*/CCl_4_, 1/3) δ_H_ (ppm): 1.41 (s, 6H, C(Me)_2_), 3.38 (t, ***J*** = 1.8 Hz, 2H, 7-CH_2_), 3.91 (s, 3H, OCH_3_), 5.05 (t, ***J*** = 1.8 Hz, 2H, 4-CH_2_), 7.07–7.13 (m, 2H, 2CH_Ar_), 7.57–7.63 (m, 2H, 2CH_Ar_), 9.76 (s, 1H, CH). ^13^C NMR (75.462 MHz, DMSO-*d_6_*/CCl_4_, 1/3) δ_C_: 26.02 (2Me), 36.15 (7-CH_2_), 55.06 (OCH_3_), 58.36 (4-CH_2_), 69.53 (C^6^), 95.43 (C), 113.74 (2CH_Ar_), 119.14 (C), 119.60 (C), 122.25 (C), 127.10 (2CH_Ar_), 129.04 (C), 130.44 (C), 135.75 (1-CH), 136.51 (C), 145.46 (C), 147.85 (C), 151.77 (C), 159.63 (CO). Anal. calcd for C_21_H_18_N_6_O_3_S: C 58.05; H 4.18; N 19.34; S 7.38%. Found: C 58.21; H 4.13; N 19.42; S 7.27.
10-(4-ethoxyphenyl)-6,6-dimethyl-6,7-dihydro-4*H*-pyrano[4″,3″:4′,5′][1,2,4]triazolo[4″,3″:1′,6′]pyrido[3′,2′:4,5]thieno[3,2-*d*][1,2,3]triazin-11(10*H*)-one (**15**)
Yellow solid (1.3 g, 96.9%), m.p.: 329–330 °C; IR ν/cm^−1^: 3107 (N=CH); 1682 (CO). ^1^H NMR (300 MHz, DMSO-*d_6_*/CCl_4_, 1/3) δ_H_ (ppm): 1.41 (s, 6H, C(Me)_2_), 1.45 (t, ***J*** = 7.0 Hz, 3H, OCH_2_C*H_3_*), 3.40 (t, ***J*** = 1.8 Hz, 2H, 7-CH_2_), 4.15 (q, ***J*** = 7.0 Hz, 2H, OC*H_2_*CH_3_), 5.05 (t, ***J*** = 1.8 Hz, 2H, 4-CH_2_), 7.06–7.13 (m, 2H, 2CH_Ar_), 7.54–7.61 (m, 2H, 2CH_Ar_), 9.83 (s, 1H, CH). ^13^C NMR (75.462 MHz, DMSO-*d_6_*/CCl_4_, 1/3) δ_C_: 14.37 (Me), 26.05 (2Me), 36.19 (7-CH_2_), 58.36 (4-CH_2_), 63.19 (OCH_2_), 69.59 (C^6^), 95.44 (C), 114.20 (2CH_Ar_), 119.13 (C), 119.66 (C), 122.32 (C), 127.14 (2CH_Ar_), 129.12 (C), 130.31 (C), 135.84 (1-CH), 136.57 (C), 145.52 (C), 147.91 (C), 151.86 (C), 158.99 (CO). Anal. calcd for C_22_H_20_N_6_O_3_S: C 58.92; H 4.49; N 18.74; S 7.15%. Found: C 59.04; H 4.53; N 18.61; S 7.08.
10-(3-chlorophenyl)-6,6-dimethyl-6,7-dihydro-4*H*-pyrano[4″,3″:4′,5′][1,2,4]triazolo[4″,3″:1′,6′]pyrido[3′,2′:4,5]thieno[3,2-*d*][1,2,3]triazin-11(10*H*)-one (**16**)
Pale yellow solid (1.29 g, 98.2%), m.p.: 325–326 °C; IR ν/cm^−1^: 3062, 3120 (N=CH); 1683 (CO). ^1^H NMR (300 MHz, DMSO-*d_6_*) δ_H_ (ppm): 1.39 (s, 6H, C(Me)_2_), 3.41 (t, ***J*** = 1.8 Hz, 2H, 7-CH_2_), 5.07 (t, ***J*** = 1.8 Hz, 2H, 4-CH_2_), 7.65–7.77 (m, 2H, 3CH_Ar_), 7.86–7.90 (m, 1H, CH_Ar_), 9.89 (s, 1H, CH). ^13^C NMR (75.462 MHz, DMSO-*d_6_*) δ_C_: 25.94 (2Me), 36.19 (7-CH_2_), 58.27 (4-CH_2_), 69.70 (C^6^), 119.13 (C), 119.65 (C), 122.49 (C), 125.10 (CH_Ar_), 126.33 (CH_Ar_), 129.13 (C), 129.26 (CH_Ar_), 130.43 (CH_Ar_), 132.91 (C), 136.10 (1-CH), 136.75 (C), 138.98 (C), 145.60 (C), 147.79 (C), 151.95 (CO). Anal. calcd for C_20_H_15_ClN_6_O_2_S: C 54.73; H 3.44; N 19.15; S 7.31%. Found: C 54.81; H 3.38; N 19.21; S 7.39.
10-(2-furylmethyl)-6,6-dimethyl-6,7-dihydro-4*H*-pyrano[4″,3″:4′,5′][1,2,4]triazolo[4″,3″:1′,6′]pyrido[3′,2′:4,5]thieno[3,2-*d*][1,2,3]triazin-11(10*H*)-one (**17**)
White solid (1.05 g, 85.7%), m.p.: 328–329 °C; IR ν/cm^−1^: 3115 (N=CH); 1688 (CO). ^1^H NMR (300 MHz, DMSO-*d_6_*/CCl_4_, 1/3) δ_H_ (ppm): 1.39 (s, 6H, C(Me)_2_), 3.37 (t, ***J*** = 1.8 Hz, 2H, 7-CH_2_), 5.03 (t, ***J*** = 1.8 Hz, 2H, 4-CH_2_), 5.69 (s, 2H, NCH_2_), 6.40 (dd, ***J*** = 3.2, 1.8 Hz, 1H, CH_furyl_), 6.53 (dd, ***J*** = 3.2, 0.8 Hz, 1H, CH_furyl_), 7.50 (dd, ***J*** = 1.8, 0.8 Hz, 1H, CH_furyl_), 9.81 (s, 1H, CH). ^13^C NMR (75.462 MHz, DMSO-*d_6_*/CCl_4_, 1/3) δ_C_: 26.01 (2Me), 36.16 (7-CH_2_), 45.53 (NCH_2_), 58.31 (4-CH_2_), 69.49 (C^6^), 109.88 (CH_furyl_), 110.32 (CH_furyl_), 119.13 (C), 119.62 (C), 121.46 (C), 129.08 (C), 135.73 (1-CH), 136.54 (C), 142.71 (CH_furyl_), 145.47 (C), 147.75 (C), 148.66 (C), 151.46 (CO). Anal. calcd for C_19_H_16_N_6_O_3_S: C 55.87; H 3.95; N 20.58; S 7.85%. Found: C 55.96; H 4.00; N 20.49; S 7.79.

### 3.2. Biological Evaluation

General Information: Compounds were studied for their possible neurotropic activities (anticonvulsant, sedative, and antianxiety activity) as well as side effects on 450 white mice of both sexes weighing 18–24 g and 180 male rats of the Wistar line weighing 120–140 g. All groups of animals were maintained at 25 ± 2 °C in the same room, on a common food ration with normal correspondence between periods of sleep and wakefulness. All the biological experiments were carried out in full compliance with the European Convention for the Protection of Vertebrate Animals used for Experimental and other Scientific Purposes.

#### 3.2.1. Evaluation of the Anticonvulsant Activity of the Synthesized Compounds

The anticonvulsant spectrum of action of the compounds was studied using the following tests: pentylenetetrazole (PTZ) convulsions [32,33,34,35,36], maximal electroshock seizure (MES) [36,37,38,39], thiosemocarbazide (TSC), and camphor convulsions [33,43] on outbred mice of both sexes weighing 18–24 g. The PTZ test is an experimental model for obtaining absence and myoclonic seizures, as well as for predicting the antianxiety properties of compounds. PTZ was tested on mice by subcutaneous administration of analeptic at a dose of 80 mg/kg and the effectiveness of the drugs was determined by the prevention of clonic seizures. The anticonvulsant activity of the compounds was also determined by preventing the tonic extensor phase of a convulsive seizure of maximal electroshock seizure (MES). The parameters of the maximum electric current are 50 mA, duration of 0.2 s, and oscillation frequency of 50 impulses/s. The evaluation criterion is the prevention of the tonic extensor phase of a convulsive seizure. TSC is an inhibitor of glutamic acid decarboxylase, an enzyme responsible for the synthesis of GABA. TSC was administered at a dose of 18 mg/kg subcutaneously [67]. In the experiments, the latent period, the incidence of seizures, and mortality after 24 h were recorded.

The test for camphor cramps is included in the methodological recommendation for conducting preclinical studies of drugs. In our experiments, camphor convulsions occurred after intraperitoneal injection of 0.04 mL of 5% slightly warmed camphor oil per 20 g of white mice weight (1 g/kg animal weight). The effect of drugs on survival and the nature of seizures caused by camphor is being studied. The experimental results were taken into account in an alternative form, and the average effective doses were determined for the active compounds.

The substances were administered intraperitoneally in doses of 10, 25, 50, 75, 100, 200, and 300 mg/kg in suspension with carboxymethylcellulose with Tween-80 (“Ferak Berlin”) 45 min before the administration of convulsants and application of the electrical stimulation. Each dose of the compound in each test was studied in five animals. The control animals were administered an emulsifier. Analogs of comparison were an anticonvulsant drug from the succinimide ethosuximide group (neuraxpharm Arzneimittel GmbH, Elisabeth-Selbert-Straße, 23, 40764 Langenfeld, Germany) and the tranquilizer diazepam [40]. A comparison drug, ethosuximide, in doses from 100 to 300 mg/kg was administered intraperitoneally as well as diazepam at a dose of 2 mg/kg. The acute daily toxicity of the drugs was tested in mice. The experimental results were statistically processed by calculating their effective 50% (ED_50_) and neurotoxic 50% (TD_50_) doses and using Student’s *t*-test. Therapeutic (TI = LD_50_/ED_50_) and protective (TI = TD_50_/ED_50_) indices were calculated [42,43].

#### 3.2.2. Evaluation of the Psychotropic Properties of the Synthesized Compounds

The psychotropic properties of the compounds were studied using the following tests: “open field”, “elevated plus maze”, and “forced swimming”, as well as the effect on the “electroshock retrograde amnesia” model.

The exploratory motor behavior of rats weighing 120–140 g was studied using a modified “open field” model [44,45,46]. For this purpose, an installation was used, the bottom of which was divided into squares with holes (cells). The experiments were carried out in the daytime under natural light. During the 5 min experiment, we determined the indicators of sedative and activating behavior: the number of horizontal movements, standing on the hind legs (vertical movements), and sniffing the cells. The number of animals in this model was eight for each compound, control, and comparison analog. The test compounds were administered to rats at the most effective dose of 50 mg/kg intraperitoneally.

Antianxiety, sedative, and antidepressant effects were studied using the “elevated plus maze” model in mice [47,48,49]. Normal animals prefer to spend most of their time in the closed arms of the maze. The antianxiety effect of the drug is assessed by an increase in the number of entries into the open arms of the maze and the time spent in them, without an increase in general motor activity. In this case, the time spent in the closed arms and the number of attempts to enter the center of the installation are recorded. In the above model, test compounds and comparison drugs were administered intraperitoneally prior to experiments. The control animals were administered an emulsifier. The results were processed statistically (*p* ≤ 0.05).

To assess “despair and depression”, the “forced swimming” model was used [50,51,52]. Experimental animals were forced to swim in a glass container (height 22 cm, diameter 14 cm) filled 1/3 with water. Intact mice swim very actively but are quickly forced to immobilize. The latent period of immobilization and the total duration of active swimming and immobilization are fixed for 6 min. The experiments were carried out under natural lighting conditions.

The antiamnesic properties of the compounds were studied using the model of electroshock retrograde amnesia according to the modification method of J. Buresh and O. Bureshova [53,54]. In rats, a conditioned passive avoidance reaction (CRPA) in a dark–light setting was developed while the time required to train the animals with CRPA was recorded. The residence time in the light and dark compartments was recorded for 5 min (300 s). Then, in the dark compartment, the animal received a single current shock (0.4 mA) through the electrode floor (training). To obtain amnesia, a maximum electroconvulsive seizure is created in animals (conducting a maximum electric current of 50 Hz; 0.2 s through corneal electrodes). Conducting an electric shock immediately after training causes the erasure of the memory trace. The test for reproduction is carried out 24 h after training—after the development of CRPA. During this period, the control animals forget the training and prefer to be in the dark part of the chamber. An increase in the time spent by rats in the light compartment compared to the control on the second day indicates the presence of antiamnestic properties of the compounds.

The side neurotoxic (muscle relaxant) effects of the substances have also been studied. Myorelaxation was studied using the “rotating rod” test in mice [33,41]. For this purpose, mice were placed on a metal rod with a corrugated rubber coating, which rotated at a speed of 5 revolutions per minute. The number of animals unable to stay on it for 2 min was determined.

#### 3.2.3. Histopathological Examination

For histopathological evaluation of the brain tissue, we conducted experiments on 24 male and female mice weighing 18–24 g.

Design of pentylenetetrazole-induced seizures: Novel compound **13** was administered via intraperitoneal injection at a 50 mg/kg dose for 3 successive days. To model the seizures, 1 h after the injections, all animals from the control group and experimental group 1 received the same dose of PTZ via subcutaneous injection.

The animals were divided into 4 groups (n = 6 in each group):

- Intact group: normal animals;
- Pentylenetetrazole-treated group;
- Experimental group 1: animals treated with compound **13**;
- Experimental group 2: animals treated with PTZ and compound **13**.

After day 3 of the experiment, the mice were sacrificed under anesthesia (40 mg/kg Nembutal Sodium via intraperitoneal injection). Brain tissue was collected and stored in a 10% neutral buffered formalin. The samples were dehydrated and embedded in paraffin; 3–5 μm microtome sections were prepared. Brain tissues were cut into coronal sections and stained with Nissl stain.

For morphometric analysis, ten samples of the hippocampal CA1 region and entorhinal cortex were photographed at 400× magnification using an AmScope MU500 5MP USB2.0 Microscope Digital Camera and Software AmScop 3.7.11443 (USA). In the CA1 region, we counted normal neurons with a light nucleus. In the entorhinal cortex, we counted neurons of layers II and III, microglial cells, and astrocytes. All cells were counted with ImageJ 1.x software using Cell Counter Plugin.

Statistical analysis: All data are expressed as mean ± SD. Statistical data analysis was performed using IBM SPSS Statistics 22.0.0 software with one-way ANOVA and the Levene test for equality of variances followed by the nonparametric Games–Howell post hoc test.

### 3.3. Docking Studies

#### 3.3.1. Design of Molecular Models

To create three-dimensional molecular models of the studied compounds, the ChemOffice program version 13.0 was used [68]. Minimization and stabilization of the obtained 3D structures were performed by using force fields MM2, which are used to optimize small molecule models [69]. The molecular models of the studied compounds were saved in *.PDB format after optimization. Molecular models of the studied targets were taken from the RCSB [70] with GABA_A_ receptor identification number PDBID:4COF. The molecular model of diazepam was taken from the database https://pubchem.ncbi.nlm.nih.gov/ (assessed on 15 June 2024) with identification number CID:3016.

#### 3.3.2. Molecular Docking

Molecular docking was carried out using the AutoDock Vina v.1.5.7 software package, which provides detailed information about the standards, protocols, and algorithms of the molecular docking program [71]. The reliability of the docking results was ensured by a 5-fold repeatability of 20 initial conformations. The virtual box volume does not exceed 27,000 Å^3^. The exhaustiveness equally is 200. The selection of the best conformers was based on the values of the standard deviation during complexation RMSD ≤ 2 Å. The criteria for the radius of interaction were calculated according to the standard: hydrogen bond length—3.4 Å; Coulomb interactions—9 Å; and van der Waals interactions—14 Å.

#### 3.3.3. Binding Constant Calculation

To determine the binding constant [72] of the studied compounds with targets, the following equations were used:

$$\Delta G_{\exp}=-RT\ln\left(\frac{1}{K}\right)$$

where Δ*G_exp_*—interaction energy, *R*—gas constant, *T*—absolute temperature, and *K*—binding constant.

#### 3.3.4. Conformational Analysis and Visualization

To identify the types of binding during the complexation of the studied compounds with targets, the Discovery Studio Visualizer [73] was used. The calculated criteria for the radius of interaction were calculated according to the standard.

#### 3.3.5. Statistical Analysis

Statistical analysis of the research results was carried out on the basis of a comprehensive application of standard statistical methods, including calculations of standard deviations, average values, and standard average errors using MS Excel 2016.

## 4. Conclusions

New tetracyclic pyrano[3,4-*c*]thieno[3,2-*e*][1,2,4]triazolo[4,3-*a*]pyridines were synthesized by the optimization of a previously used method. Moreover, a new pentacyclic heterocyclic system—pyrano[4″,3″:4′,5′][1,2,4]triazolo[4″,3″:1′,6′]pyrido[3′,2′:4,5]thieno[3,2-*d*][1,2,3]triazin-11-ones—were synthesized based on them. The psychotropic activity of sixteen newly synthesized tetra- and pentacyclic heterocyclic compounds was studied. The evaluation of the anticonvulsant activity of all synthesized compounds revealed that, except for compounds **2**–**4**, all compounds exhibited pronounced anticonvulsant action. It should be noted that, according to the PTZ test, these compounds appeared to be more active than ethosuximide. The most active compounds (**5**–**17**) were studied for their potential antianxiety and antidepressant activities. According to the “open field” behavioral model, all compounds statistically significantly decreased both horizontal and vertical movements, suggesting a sedative effect. However, compound **13** stands out as it increased vertical movements, leading to the activation of behavior. Additionally, the compounds increased the number of sniffing cell examinations, suggesting potential antianxiety activity. It should be noted that compound **13**, like diazepam, exhibits activating behavior and the same anxiolytic effect. The results of the EPM model confirmed the antianxiety effect of all tested compounds, especially compound **13**. According to the “forced swimming” model, all tested compounds, except for compounds **7**, **10**, and **11**, showed some antidepressant effect. These compounds, along with diazepam, increased the total time of active swimming, suggesting an antidepressant effect similar to that of diazepam. Once again, compound **13** emerged as the most active. In the “electroshock retrograde amnesia” model, all tested compounds showed an antiamnestic effect similar to piracetam. Histological and pathological analyses of brain tissues revealed that compound **13** induces moderate morphological alterations in the hippocampus and entorhinal cortex. Importantly, these studies also demonstrated that compound **13** possesses neuroprotective properties in the entorhinal cortex, effectively mitigating gliosis and neuronal loss triggered by PTZ. This evidence highlights the potential of compound **13** as a protective agent against PTZ-induced neurodegeneration, offering promising avenues for future therapeutic applications. Docking analysis and obtained biophysics properties of complexations showed that among sixteen compounds, only compounds **7**, **8**, and **13** interacted with the GABA_A_ receptor. The results indicate that the maximum binding constant was observed in compound **13.**

## Figures and Tables

**Table 1:** Yields and functional groups of tetracyclic (**2**–**9**) and pentacyclic (**10**–**17**) heterocyclic compounds.

Compound	R	Yield (%) ^1^	Compound	R	Yield (%) ^1^
**2**	H	93.0	**10**	H	85.9
**3**	C_6_H_5_	92.0	**11**	C_6_H_5_	86.6
**4**	CH_2_C_6_H_5_	95.0	**12**	CH_2_C_6_H_5_	86.2
**5**	(CH_2_)_2_C_6_H_5_	87.1	**13**	(CH_2_)_2_C_6_H_5_	87.8
**6**	C_6_H_4_-4-OMe	89.1	**14**	C_6_H_4_-4-OMe	94.8
**7**	C_6_H_4_-4-OEt	91.4	**15**	C_6_H_4_-4-OEt	96.9
**8**	C_6_H_4_-3-Cl	93.0	**16**	C_6_H_4_-3-Cl	98.2
**9**	2-furylmethyl	81.8	**17**	2-furylmethyl	85.7

^1^ Yield after recrystallization.

**Table 2:** Anticonvulsant activity by PTZ antagonism, myorelaxation, and maximum tolerated doses (MTDs) of the examined compounds **5**–**17** (intraperitoneal injection).

Compounds (50 mg/kg) n = 8	Antagonism by PTZ (ED_50_, mg/kg) *	Myorelaxation (TD_50,_ mg/kg) *	MTD, (mg/kg) *	TI	Latency of Convulsions Induced by TSC, min	
					M ± м	I **
**Control**	−	−	−	−	−	−
**5**	30.0 (25.0 ÷ 36)	>200	>500	>16.7	74.8 ± 12.2	1.2
**6**	40.0 (32.8 ÷ 48.8)	>200	>600	>15	80.4 ± 8.9	1.3
**7**	36.0 (23.0 ÷ 56.1)	>200	>700	>19.4	89.6 ± 7.2	1.4
**8**	41.0 (21.6 ÷ 77.9	>200	>600	>14.6	73.2 ± 9.7	1.2
**9**	34.0 (28.3 ÷ 40.8)	>200	>600	>17.6	125 ± 10.8	2.0
**10**	55.0 (45.5 ÷ 65.5)	>200	>700	>23	106 ± 10.8	1.7
**11**	35.0 (30 ÷ 41.3)	>200	>500	>14.3	96.6 ± 9.1	1.5
**12**	42.0 (35 ÷ 50.4)	>200	>800	>19	108 ± 9.1	1.7
**13**	20.0 (16.6 ÷ 24)	>200	>1000	>50	128 ±15.2	2.05
**14**	23.0 (19.2 ÷ 27.6)	>200	>800	>34.8	120.3 ± 10	1.9
**15**	28.0 (22.4 ÷ 35)	>200	>700	>25	82.0 ± 8.2	1.3
**16**	35.0 (28.2 ÷ 43.4)	>200	>500	>14.3	90.0 ± 12.3	1.4
**17**	44.0 (36.4 ÷ 53.7)	>200	>800	>18.2	108.0 ± 13.5	1.7
**Ethosuximide** **(200 mg/kg)**	155.0 (117.5 ÷ 205)	520.0 (426 ÷ 634)	1325 (1200 ÷ 1462)	8.5	118 ± 14.0	1.8
**Diazepam** **(2 mg/kg)**	0.5 (0.4÷0.7)	2.7 (1.4÷5.5)	180 (128.5 ÷ 252.0)	360	65 ± 3.5	1.04

* *p* = 0.05 at a probability level. I **—an increase in the threshold.

**Table 3:** Effect of compounds (**5**–**17**) in rats in the “open field” test.

Compounds n = 8	Dose, mg/kg	Amount (Absolute Data during 5 min) *		
		Horizontal Displacement	Vertical Displacement	Sniffing of Cells
**Control**	–	25.8 ± 3.2	6.1 ± 0.7	0.5 ± 0.2
**5**	50	17.0 ± 4.6 **	2,4 ± 0.7 **	1.4± 0.3 **
**6**	50	22.4 ± 5.4	2.2 ± 0.6 **	2.6 ± 0.6 **
**7**	50	9.2 ± 3.5 **	1.6 ± 0.5 **	0.9 ± 0.1 **
**8**	50	19.6 ± 2.1 **	2.2 ± 0.7 **	2.1 ± 0.5 **
**9**	50	17.6 ± 4.1 **	4.1 ± 0.9 **	1.9 ± 0.5 **
**10**	50	17.2 ± 3.3 **	3.3 ± 1.6 **	2.4 ± 0.6 **
**11**	50	15.0 ± 3.8 **	1.8 ± 1.3 **	1.2 ± 0.2 **
**12**	50	16.6 ± 3.3 **	3.1 ± 0.9 **	2.5 ± 0.5 **
**13**	50	37.5 ± 3.3 **	6.5 ± 1.6	3.3 ± 0.6 **
**14**	50	18.6 ± 3.0 **	2.8 ± 0.7 **	2.7 ± 0.3 **
**15**	50	17.2 ± 3.1 **	2.4 ± 0.4 **	2.0 ± 0.4 **
**16**	50	22.0 ± 2.5	2.4 ± 0.2 **	1.4 ± 0.1 **
**17**	50	13.2 ± 2.8 **	1.2 ± 0.4 **	2.2 ± 0.3 **
**Ethosuximide**	200	26.8 ± 3.8	5.8 ± 1.9	0.6 ± 0.08
**Diazepam**	2	33.6 ± 4.2 **	6.4 ± 1.0	3.2 ± 0.9 **

* *p* ≤ 0.05 at a probability level. ** The differences are statistically significant compared with the control group.

**Table 4:** Effect of compounds (**5**–**17**) on the state of “fear and despair” in mice in the EPM model (observation time of 5 min).

Compound n = 8	Time Spent in Closed Arms, /s/*	Number of Entries into the Closed Arms *	Time Spent in the Center, /s/*	Time Spent in the Open Arms, /s/*
**Control**	278.2 ± 20.0	7.0 ± 1.2	21.8 ± 4.4	–
**5 (50 mg/kg)**	222.0 ± 15.8 **	4.6 ± 0.7 **	13.0 ± 3.2 **	65.0 ± 8.7 **
**6 (50 mg/kg)**	224.0 ± 20.6 **	4.4 ± 0.8 **	45.0 ± 6.2 **	31.0 ± 7.7 ***
**7 (50 mg/kg)**	250.0 ± 17.0 **	3.8 ± 0.9 **	26.0 ± 7.1	24.0 ± 8.1 **
**8 (50 mg/kg)**	210.0 ± 18.9 **	3.9 ± 0.9 **	19.0 ± 5.1	71.0 ± 10.2 **
**9 (50 mg/kg)**	167.0 ± 15.5 **	4.8 ± 0.9 **	35.0 ± 5.7 **	98.0 ± 8.6 **
**10 (50 mg/kg)**	235.0 ± 21.0 **	4.7 ± 1.0 **	31.0 ± 5.7 **	34.0 ± 5.4 **
**11 (50 mg/kg)**	192.0 ± 16.6 **	3.8 ± 1.3 **	21.0 ± 4.2	87.0 ± 13.0 **
**12 (50 mg/kg)**	208.2 ± 19.8 **	3.9 ± 1.1 **	38.0 ± 5.5 **	54.0 ± 5.5 **
**13 (50 mg/kg)**	98.0 ± 10.1 **	3.9 ± 1.1 **	68.0 ± 9.9 **	134.0 ± 11.2 **
**14 (50 mg/kg)**	228.0 ± 23.7 **	3.6 ± 0.8 **	26.0 ± 4.2 **	46.0 ± 6.6 **
**15 (50 mg/kg)**	202.0 ± 19.9 **	2.2 ± 0.7 **	17.0 ± 3.1	81.0 ± 12.2 **
**16 (50 mg/kg)**	197.4 ± 14.7 **	4.0 ± 1.0 **	77.0 ± 8.8 **	26.0 ± 7.2 **
**17 (50 mg/kg)**	208.0 ± 20.1 **	4.2 ± 1.0 **	24.0 ± 3.5 **	68.0 ± 7.7 **
**Ethosuximide** **200 mg/kg**	245.2 ± 15.0	8.1 ± 2.5	54.8 ± 4.7 **	–
**Diazepam** **2 mg/kg**	200.5 ± 15.2 **	5.5 ± 1.2	42.5 ± 3.9 **	57.0 ± 4.2 **

* *p* ≤ 0.05 at a probability level. ** The differences are statistically significant compared with the control.

**Table 5:** The effects of compounds **5**–**17** on the “forced swimming” model (observation time of 6 min).

Compound n = 8	Dose, mg/kg	Latent Period I Immobilization, /s/*	Total Time of Immobilization/s/*	Total Time of Active Swimming, /s/*
**Control**	–	92.0 ± 7.8	81.0 ± 8.8	279.0 ± 13.3
**5**	50	134.0 ± 25.2 **	8.0 ± 2.7	352.0 ± 25.3 **
**6**	50	101.0 ± 18.6 **	7.0 ± 1.2 **	353.0 ± 21.0 **
**7**	50	–	360	–
**8**	50	105.0 ± 9.8 **	25.0 ± 7.8 **	335.0 ± 21.0 **
**9**	50	109.0 ± 10.6 **	20.0 ± 5.5 **	340.0 ± 19.9 **
**10**	50	71.0 ± 8.6	78.0 ± 2.7	282.0 ± 17.7
**11**	50	78.0 ± 15.3	108.0 ± 22.1 **	252.0 ± 20.1 **
**12**	50	104.0 ± 6.4 **	28.0 ± 6.8 **	332.0 ± 21.0 **
**13**	50	150.0 ± 17.2 **	3.0 ± 0.5 **	357.0 ± 23.3 **
**14**	50	144.0 ± 8.0 **	16.0 ± 4.8 **	344.0 ± 18.8 **
**15**	50	120.0 ± 9.7 **	10.1 ± 2.7 **	349.9 ± 20.1 **
**16**	50	124.0 ± 11.7 **	8.0 ± 1.8 **	352.0 ± 23.0 **
**17**	50	107.0 ± 5.8 **	5.9 ± 0.7 **	354.1 ± 20.7 **
**Ethosux.**	200	105 ± 9.6	98 ± 9.9 **	262 ±14.4
**Diazep.**	2	174 ± 18.1 **	24 ± 6.6 **	336 ± 18.9 **

* *p* ≤ 0.05 at a probability level. ** The differences are statistically significant compared with the control.

**Table 6:** Characterization of the parameters of amnestic functions in the model of electroshock retrograde amnesia after administration of **5**–**17** compounds (study for 5 min).

Compounds (50 mg/kg)	The Time Spent in the Light Chamber during the CRPA Training (s), First Day *	The Time Spent in the Light Chamber When Playing CRPA + MES after 24 h (s), Second Day *
**Control**	280.0 ± 7.5	281.0 ± 6.1
**5**	286.0 ± 4.3	297.4 ± 6.9 **
**6**	283.0 ± 5.2	287.0 ± 7.2
**7**	286.0 ± 5.3	289.0 ± 6.3
**8**	267.0 ± 7.2	292.2 ± 5.8 **
**9**	286.0 ± 7.3	292.0 ± 5.3
**10**	281.0 ± 6.4	287.0 ± 5.3
**11**	285.0 ± 6.8	290.0 ± 7.2
**12**	285.0 ± 6.1	295.4 ± 5.3 **
**13**	283.0 ± 5.8	298. 0 ± 5.3 **
**14**	282.0 ± 6.8	296.0 ± 5.4 **
**15**	283.0 ± 7.1	297.5 ± 6.8 **
**16**	280.0 ± 8.0	295.4 ± 5.3
**17**	285.0 ± 6.5	295.4 ± 5.3 **
**Diazepam** **(2 mg/kg)**	187.0 ± 6.7 *	126.0 ± 6.5 **
**Piracetam** **(1000 mg/kg)**	158.0 ± 5.9 *	243.7 ± 8.4 **

CRPA + MES—conditioned reaction of passive avoidance + maximal electroshock seizure; *—the significance of differences in values from the intact control without MES; and **—the significance of differences in values from the control with MES, at *p* ≤ 0.05.

**Table 7:** Energetic and biophysical indicators of complexation of the studied compounds (**7**, **8**, and **13**) and diazepam with GABAA receptor.

Structure ID	Complexation Energy kcal/mol	RMSD	Binding Constant Kb
**7**	−8.20 ± 0.41	1.773	9.4 × 10^5^
**8**	−9.23 ± 0.46	1.866	5.3 × 10^6^
**13**	−9.45 ± 0.47	1.370	7.7 × 10^6^
**Diazepam**	−7.5 ± 0.37	1.20	2.9 × 10^5^

## Data Availability

Data is contained within the article and Supplementary Materials.

## Supplementary Materials

The following supporting information can be downloaded at: https://www.mdpi.com/article/10.3390/ph17070829/s1.
