# Supplementary material for: Synthesis and Psychotropic Properties of Novel Condensed Triazines for Drug Discovery
Source: Pharmaceuticals (Basel). 2024 Jun 25;17(7):829. doi: 10.3390/ph17070829 (PMC11280098; doi:10.3390/ph17070829)
Supplement: Supplementary file 1 [file pharmaceuticals-17-00829-s001.zip › pharmaceuticals-3008536-supplementary.pdf]

^{1}H and ^{13}C NMR spectrum of compound 5

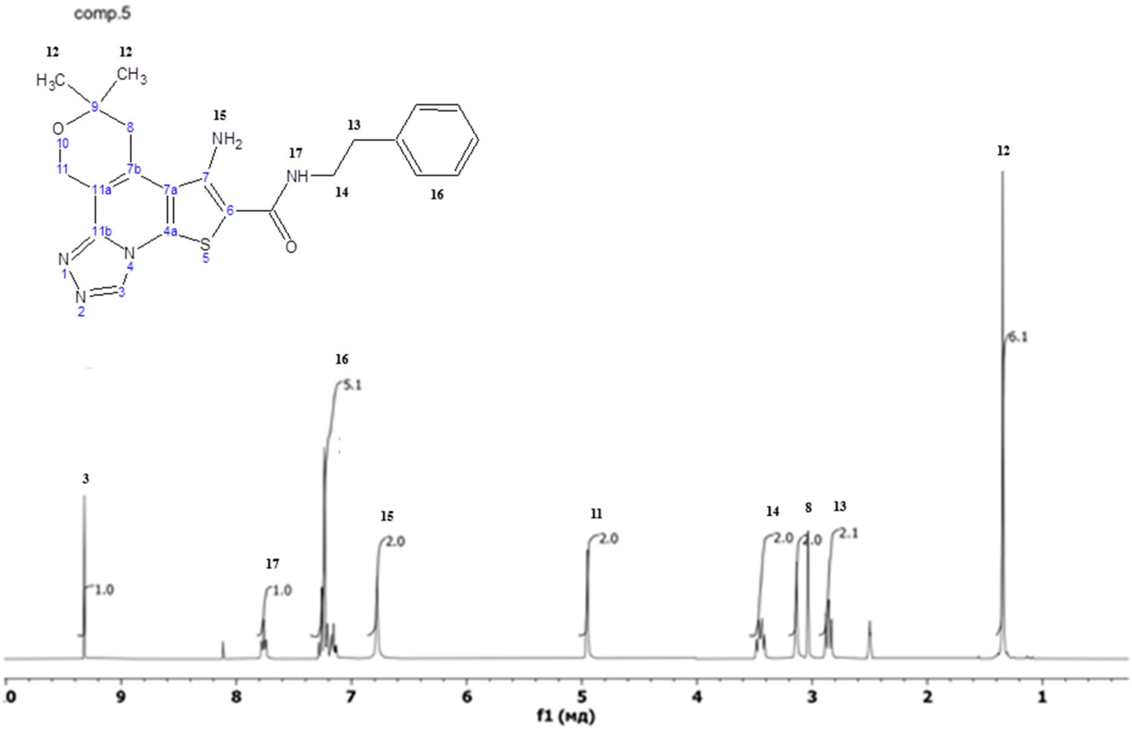

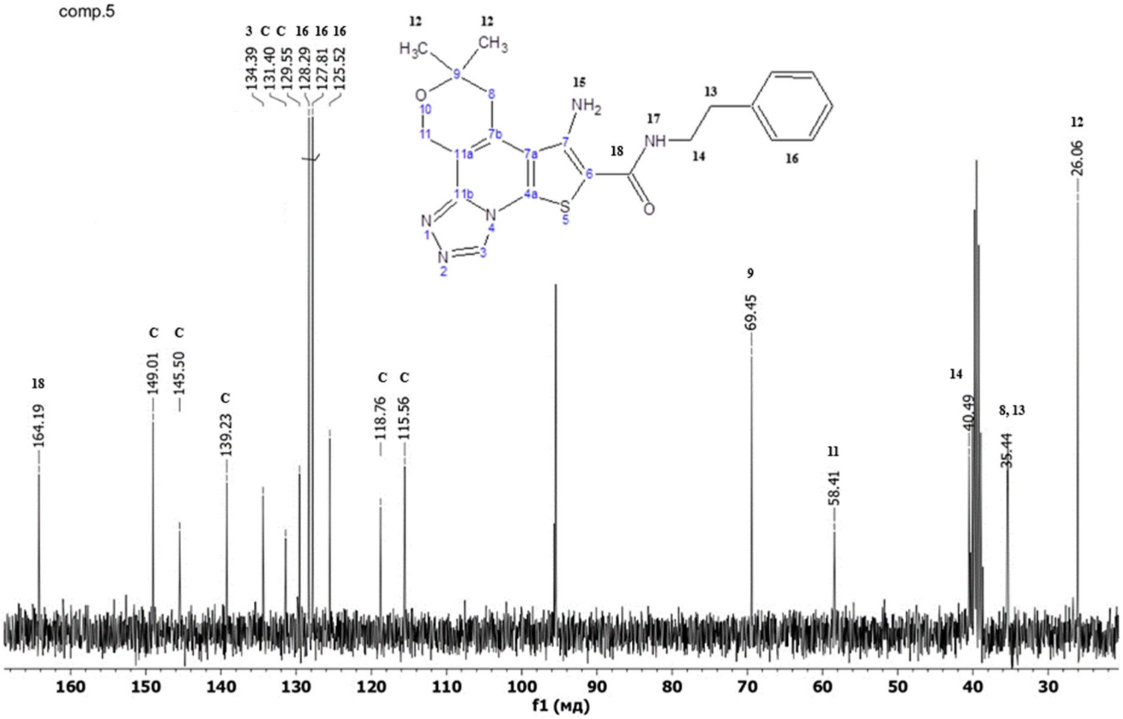

DEPT ^{13}C spectrum of compound 5

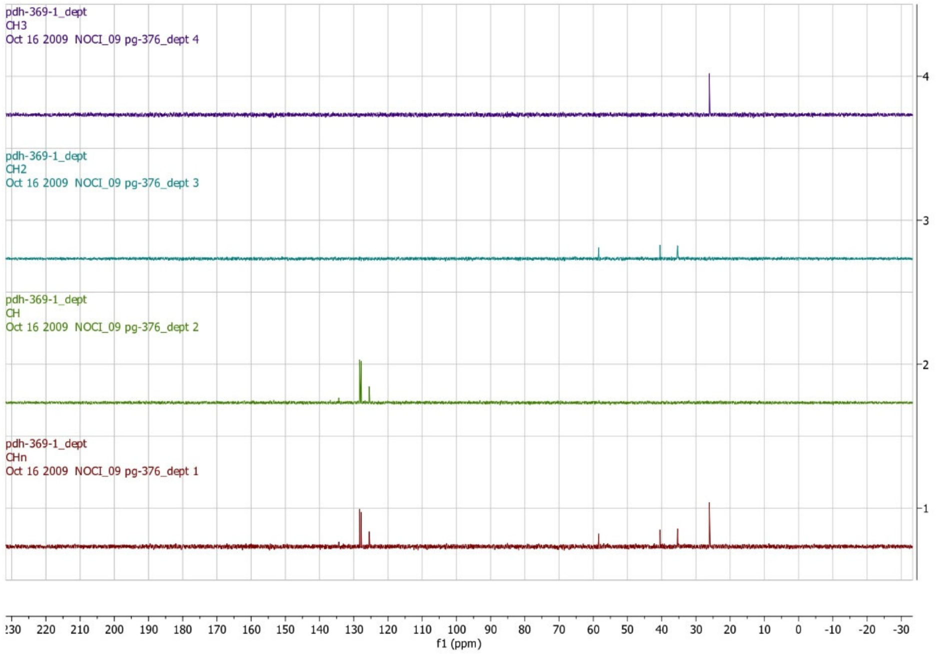

^{1}H and ^{13}C NMR spectrum of compound 6

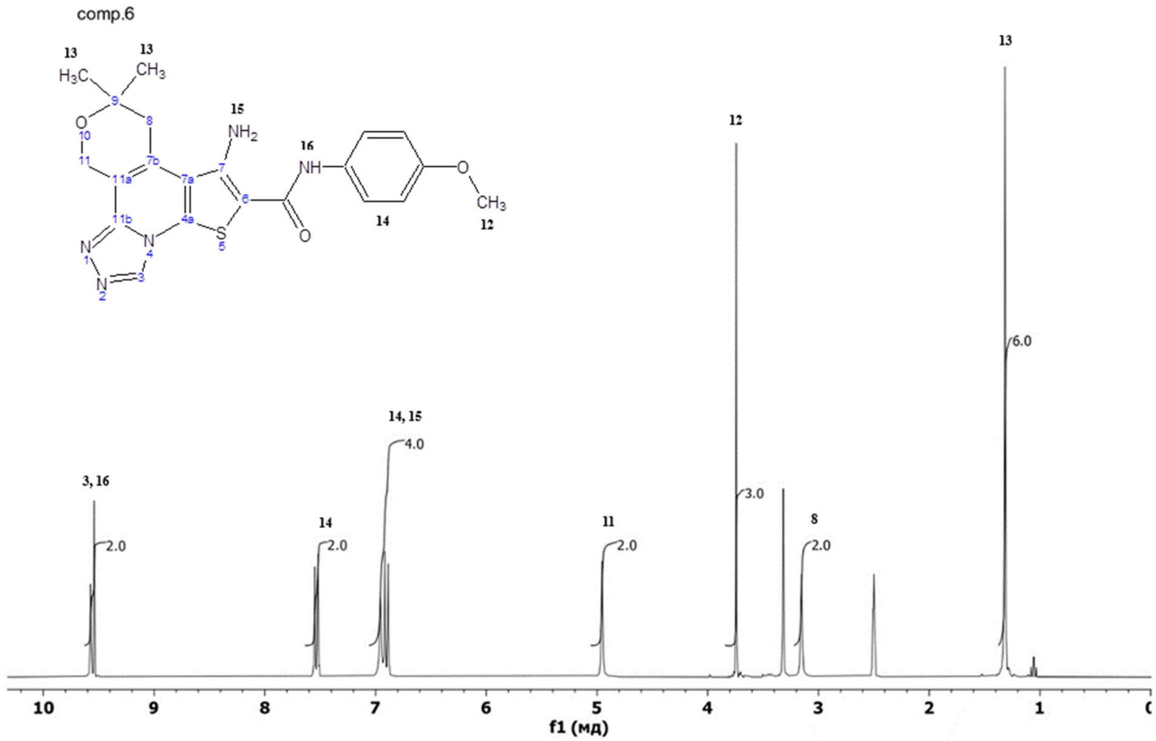

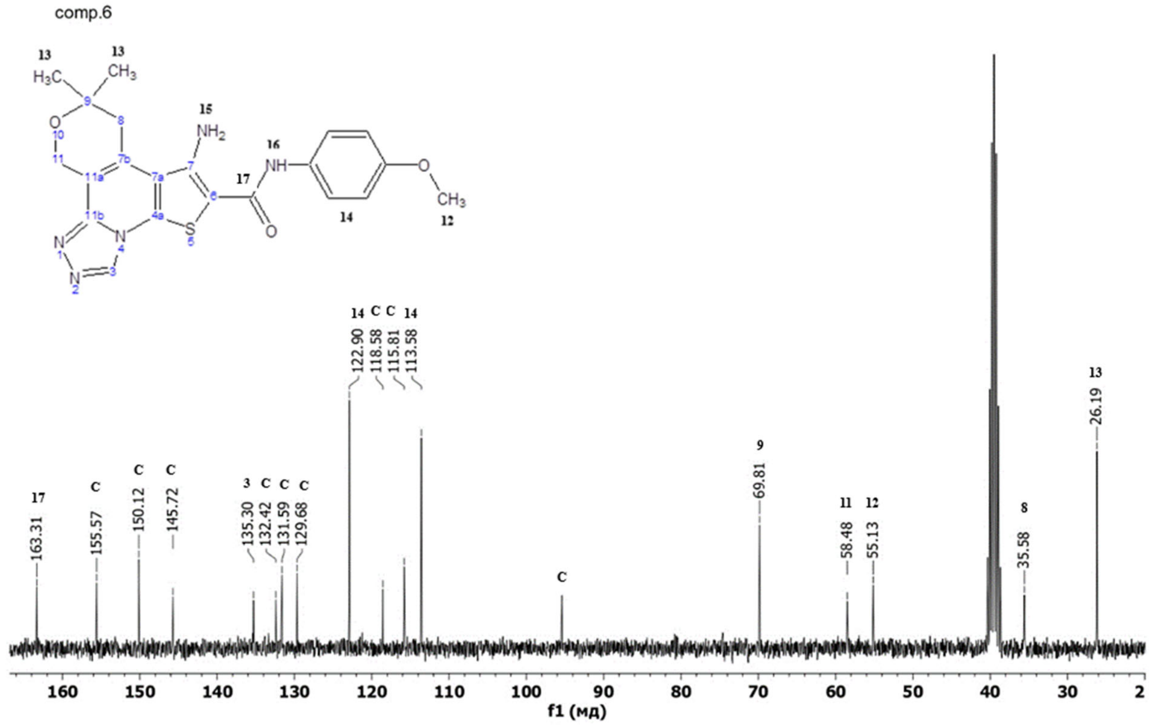

^{1}H and ^{13}C NMR spectrum of compound 7

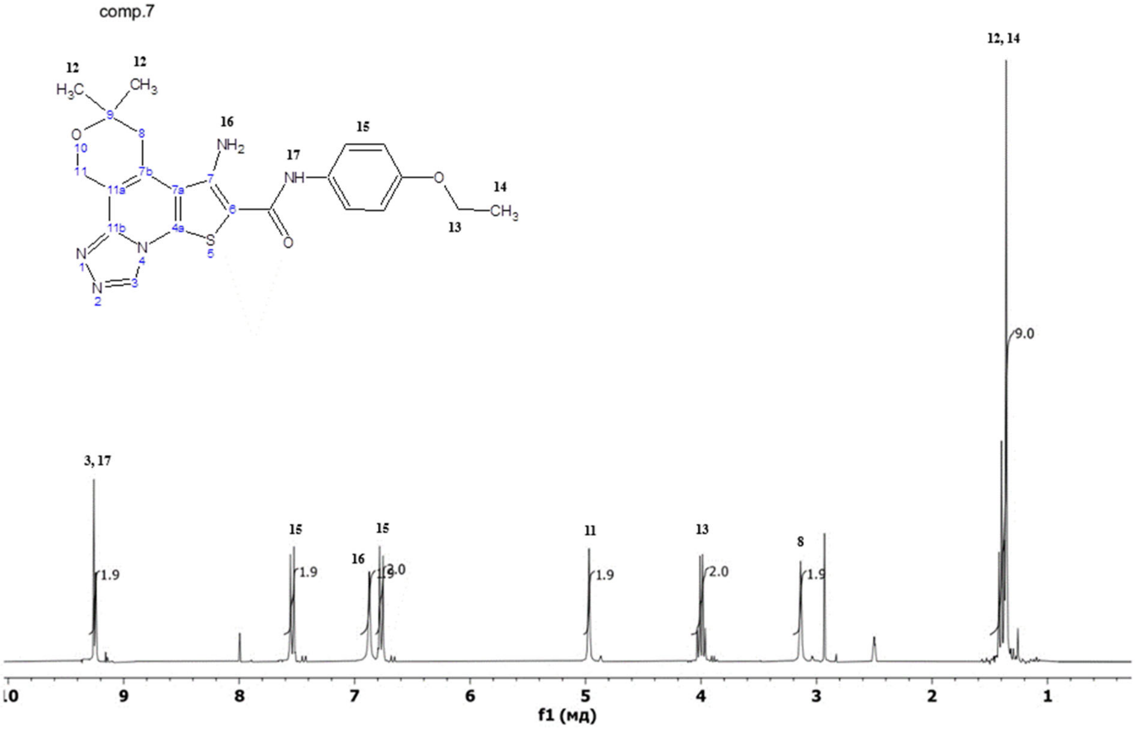

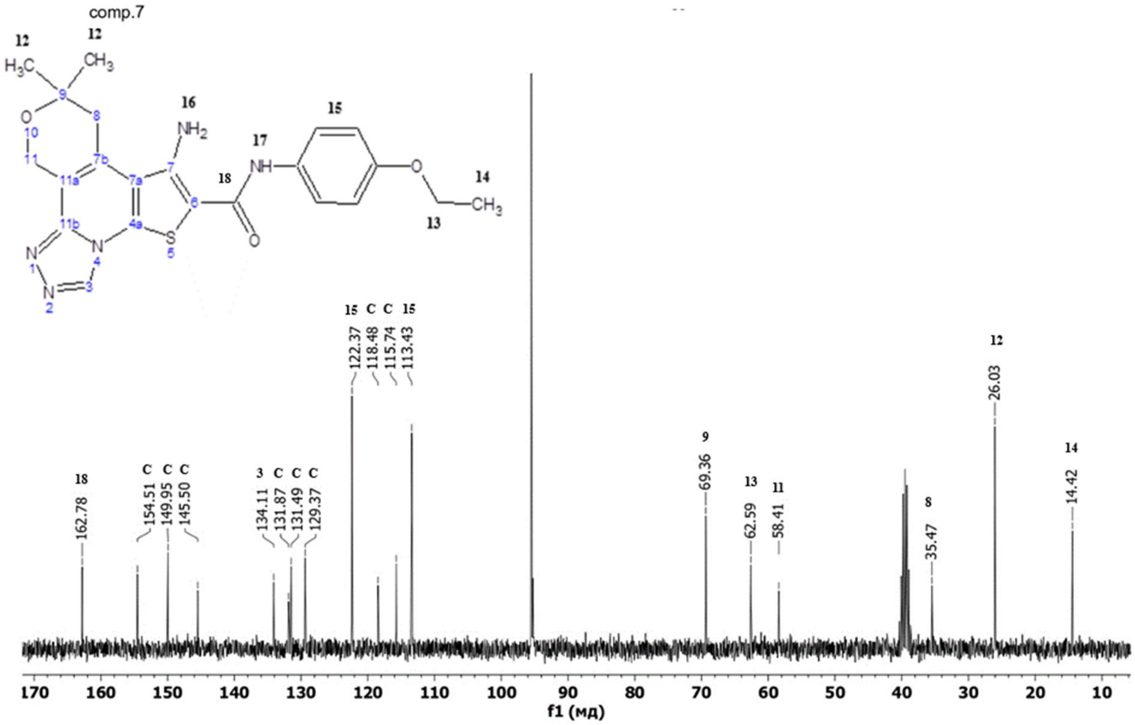

^{1}H and ^{13}C NMR spectrum of compound 8

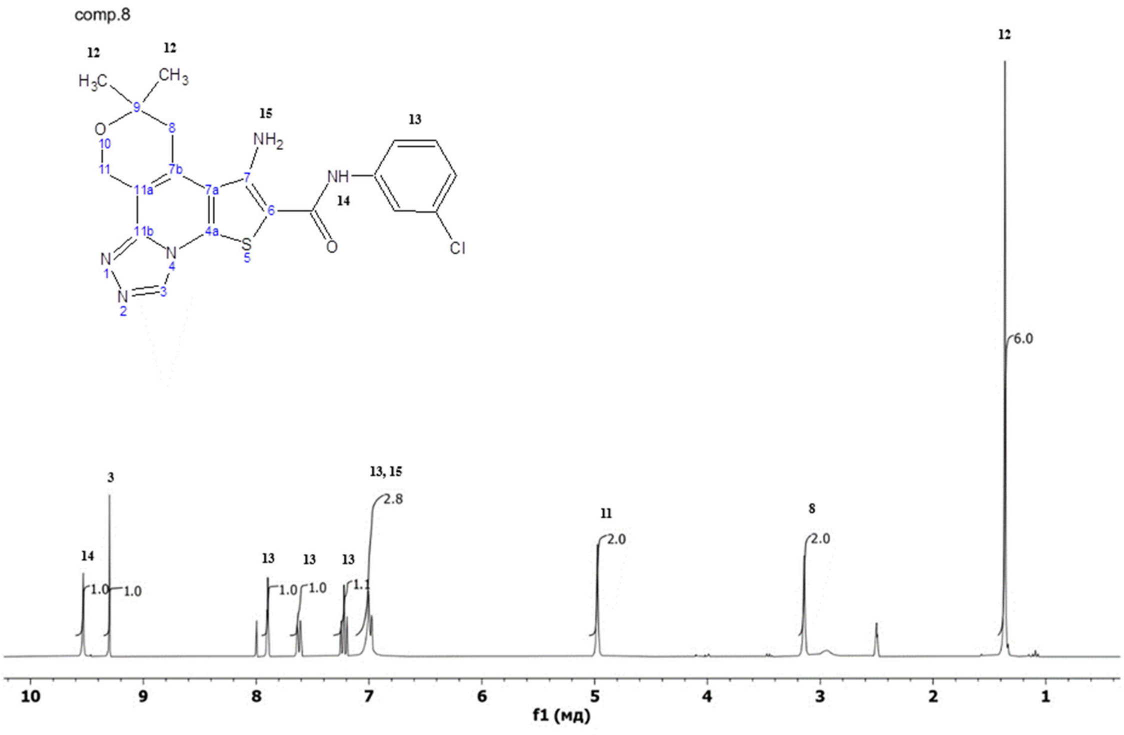

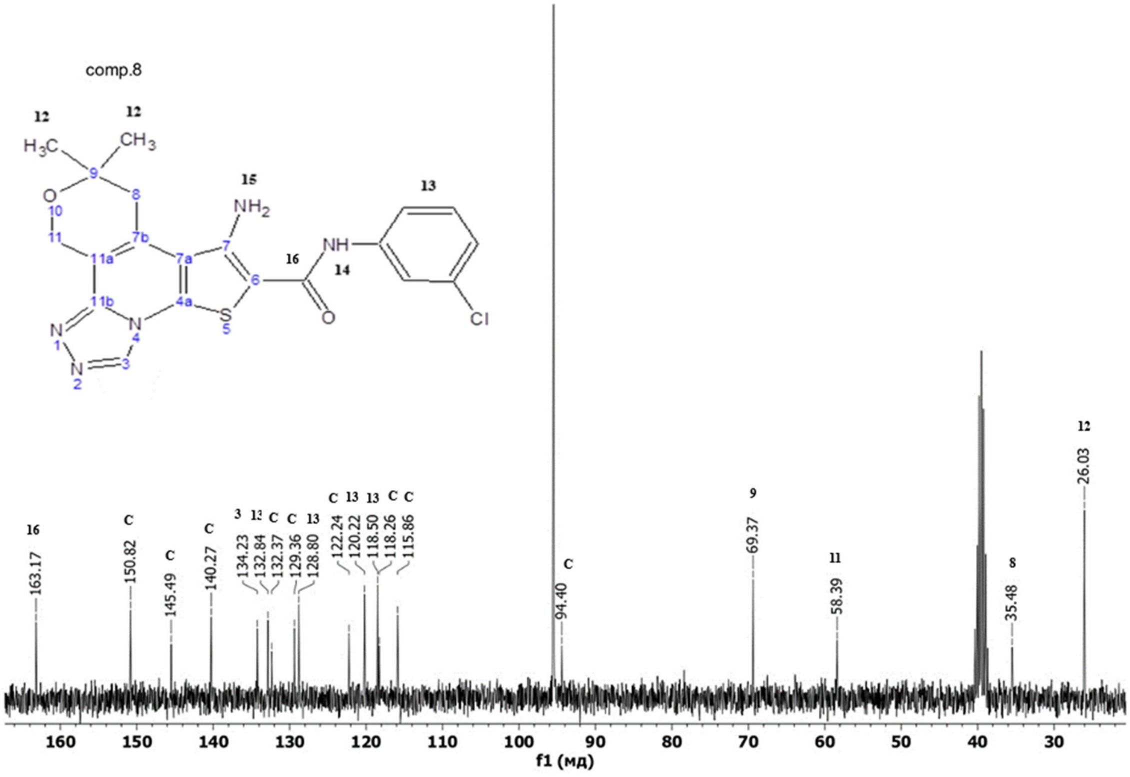

^{1}H and ^{13}C NMR spectrum of compound 9

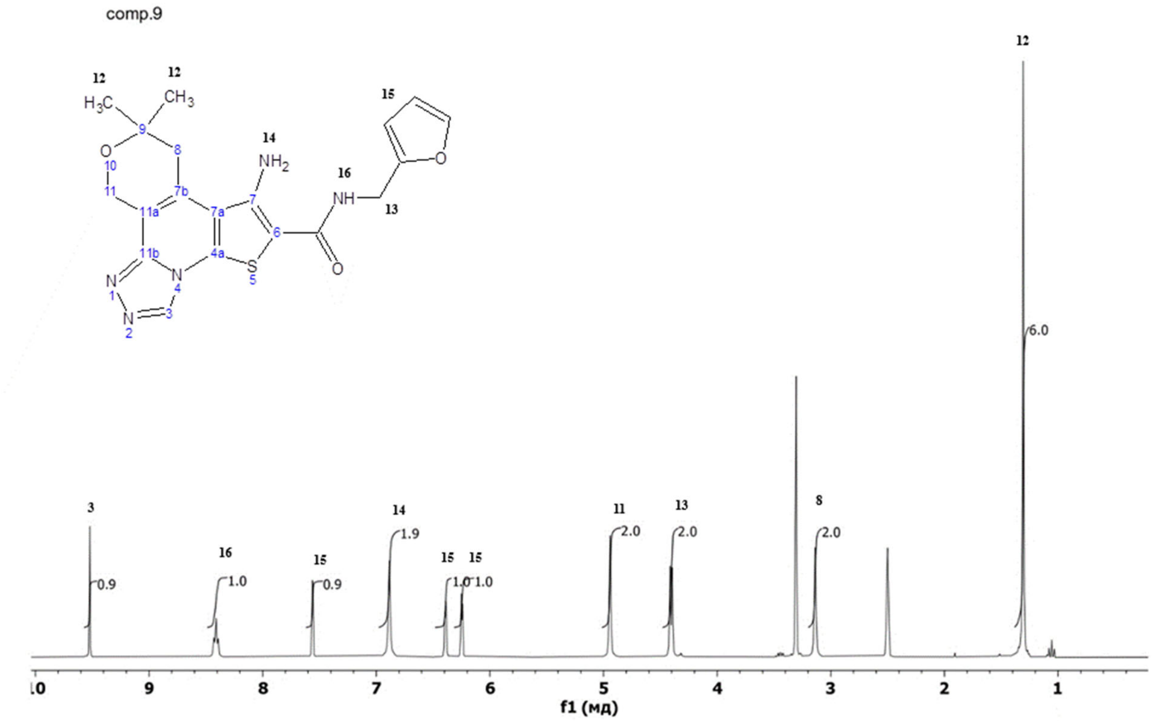

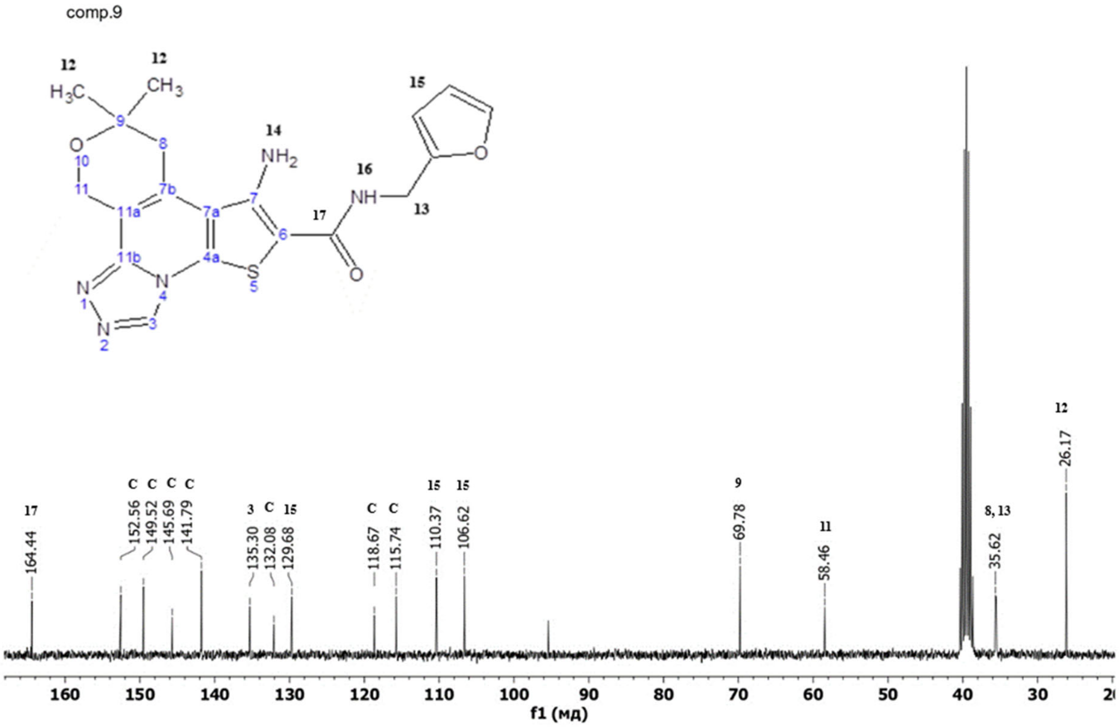

¹H and ¹³C NMR spectrum of compound 10

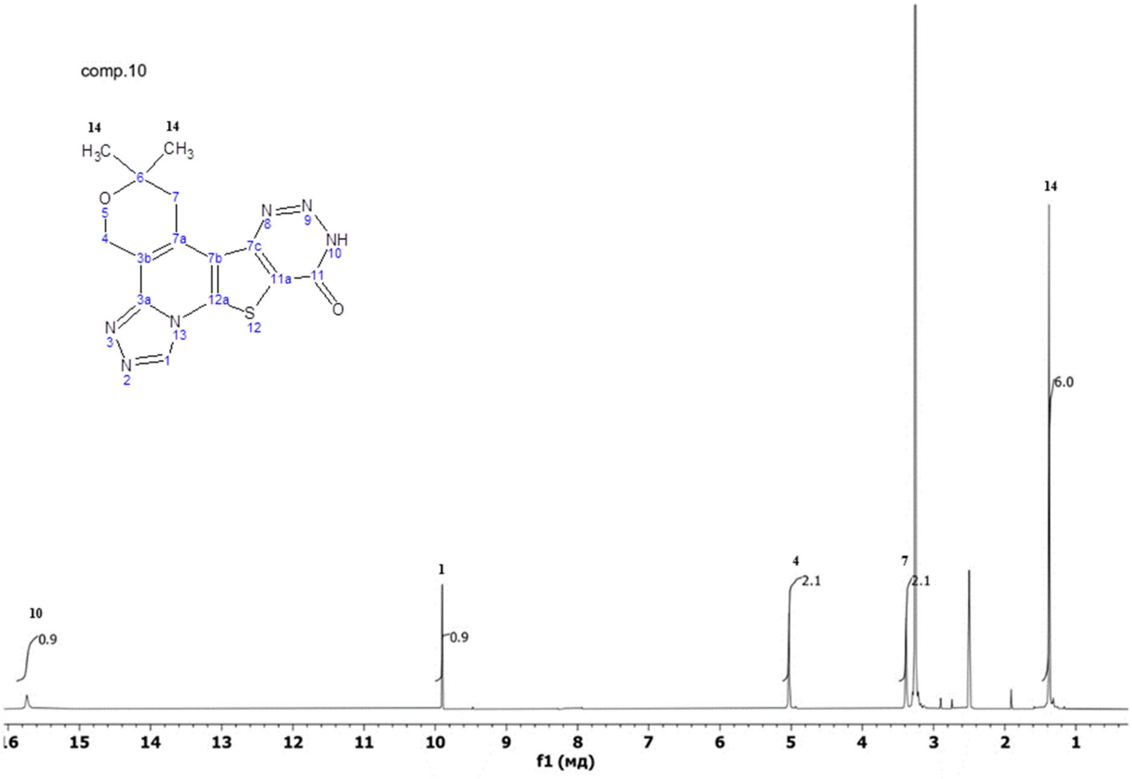

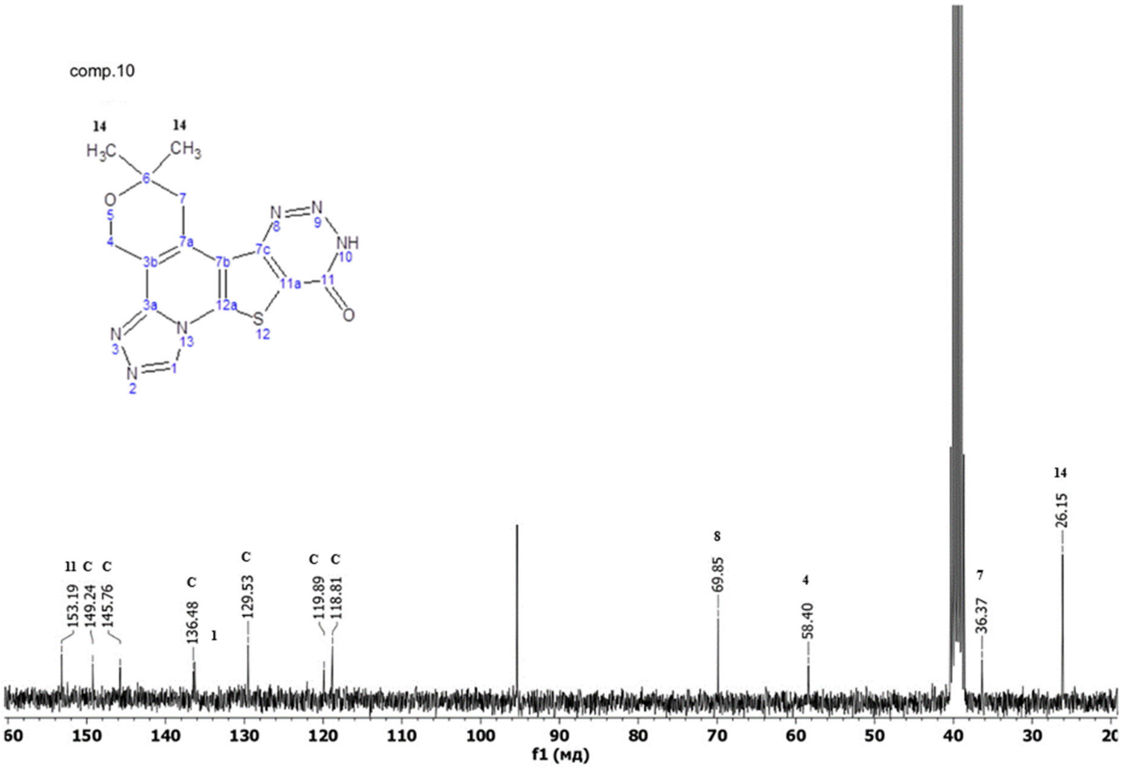

^{1}H and ^{13}C NMR spectrum of compound 11

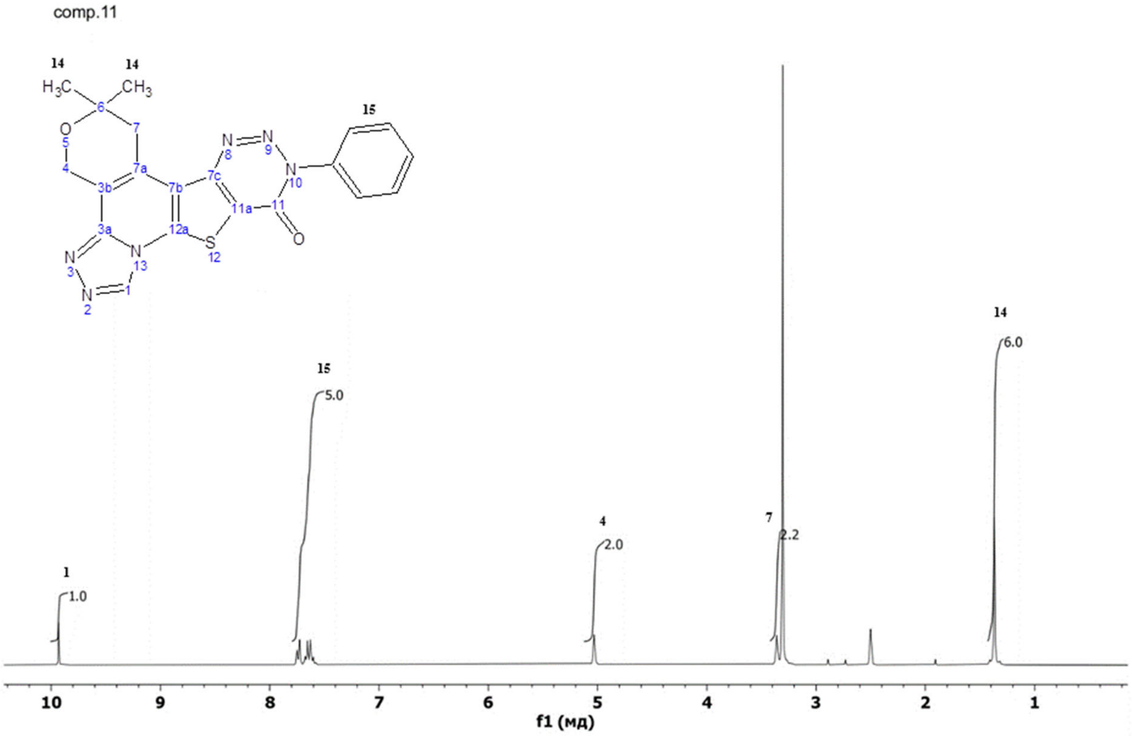

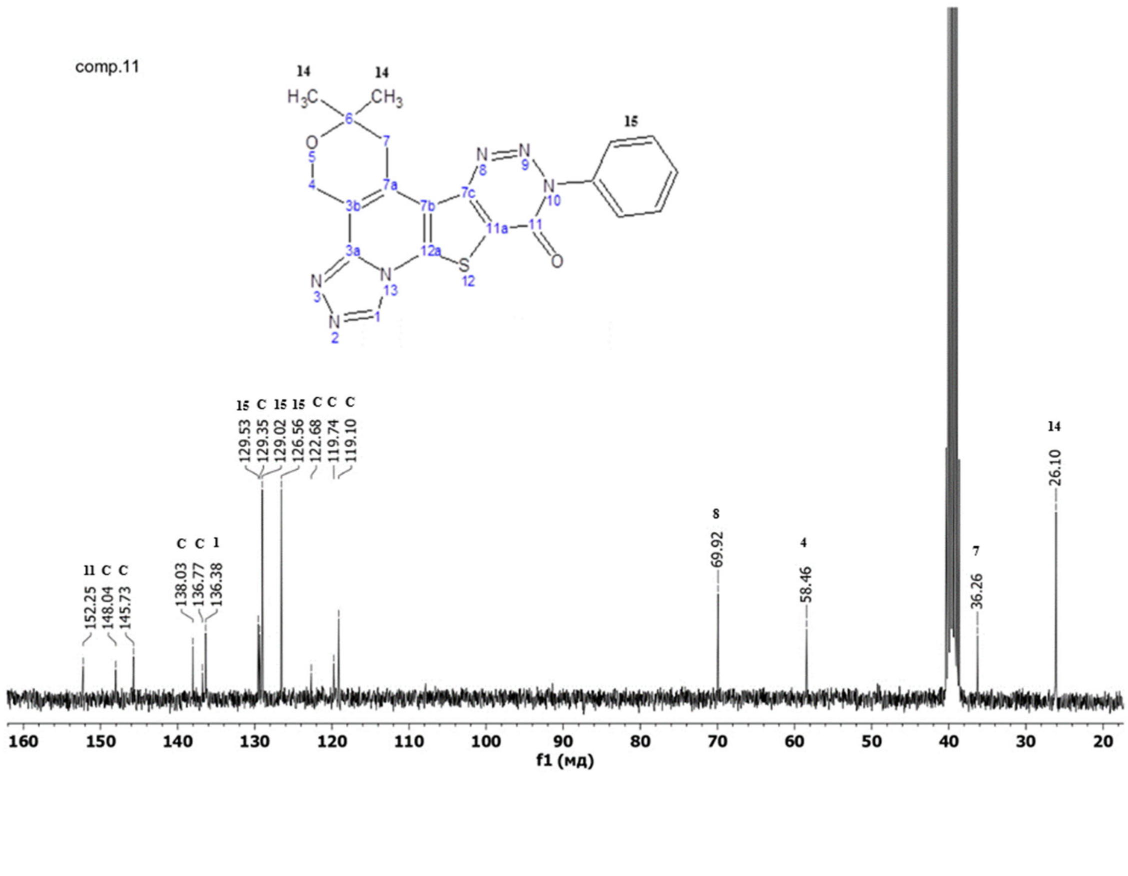

^{1}H and ^{13}C NMR spectrum of compound 12

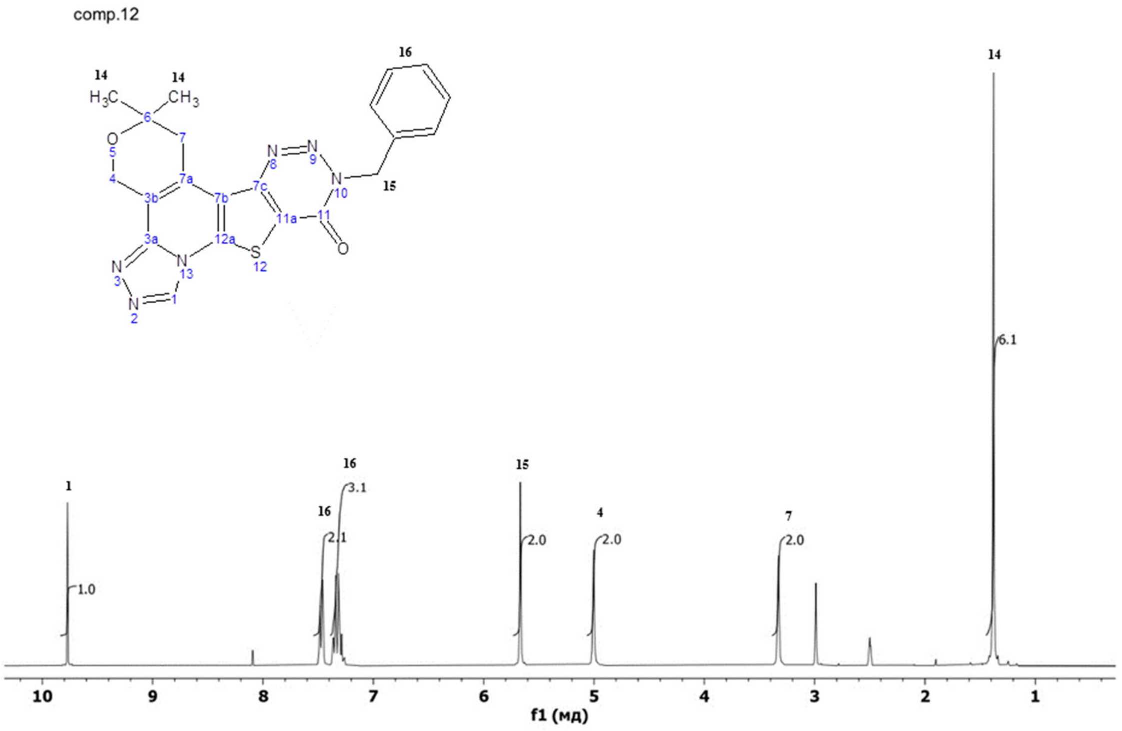

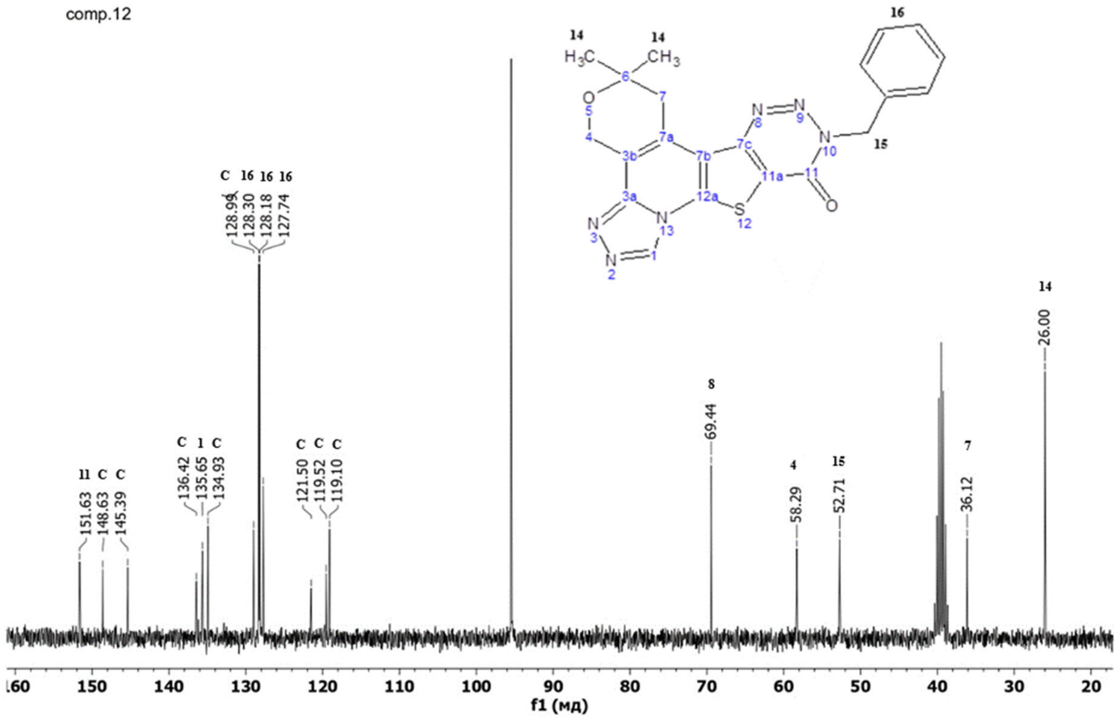

DEPT ^{13}C spectrum of compound 12

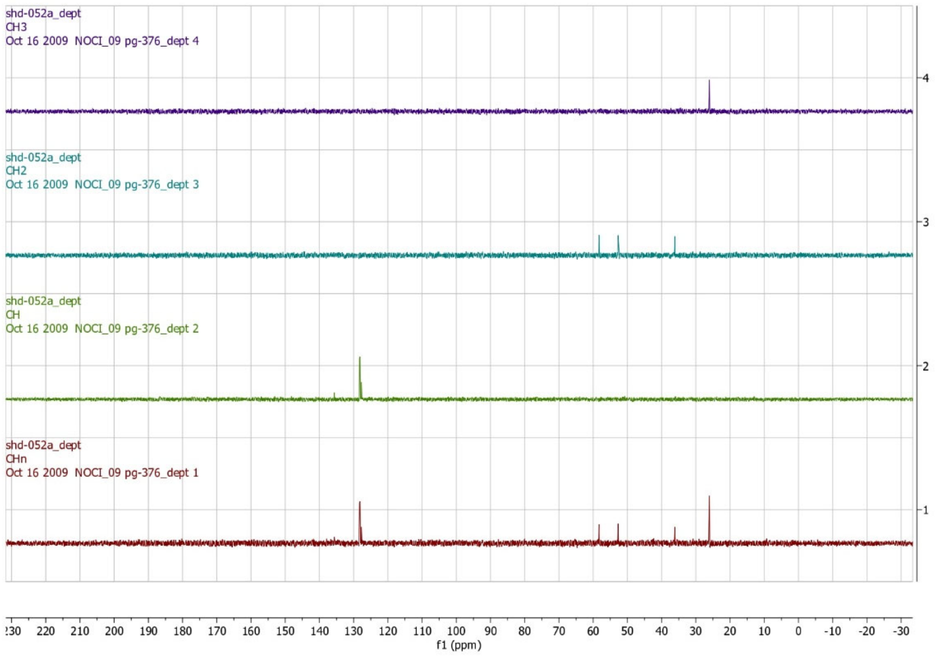

^{1}H and ^{13}C NMR spectrum of compound 13

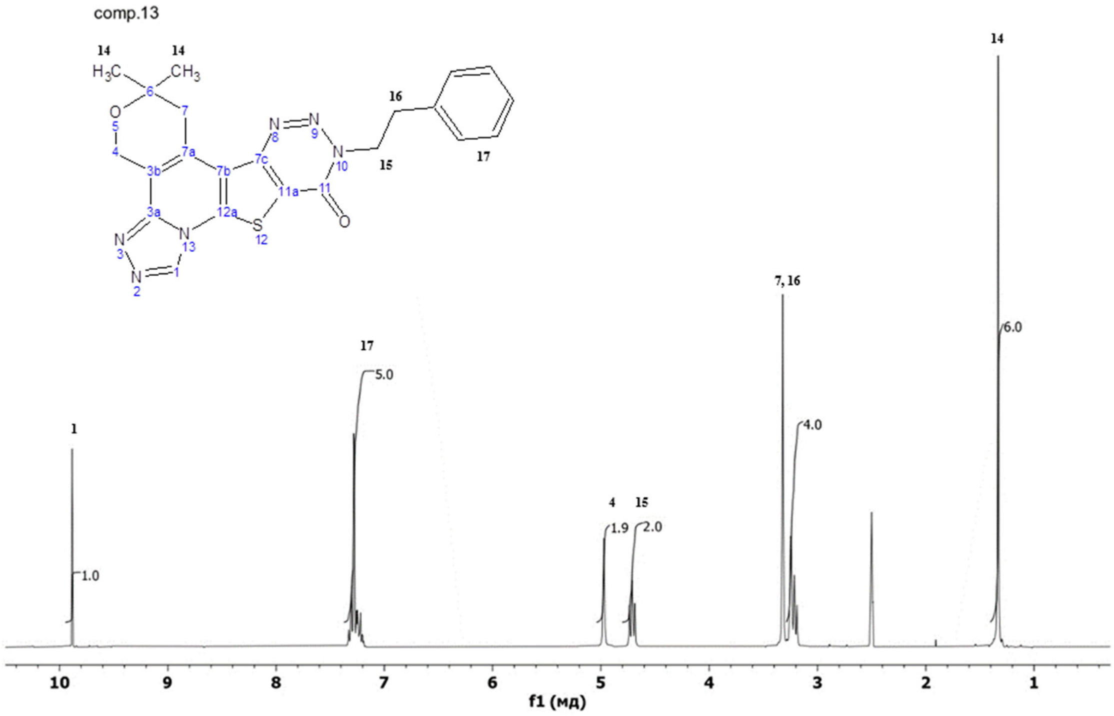

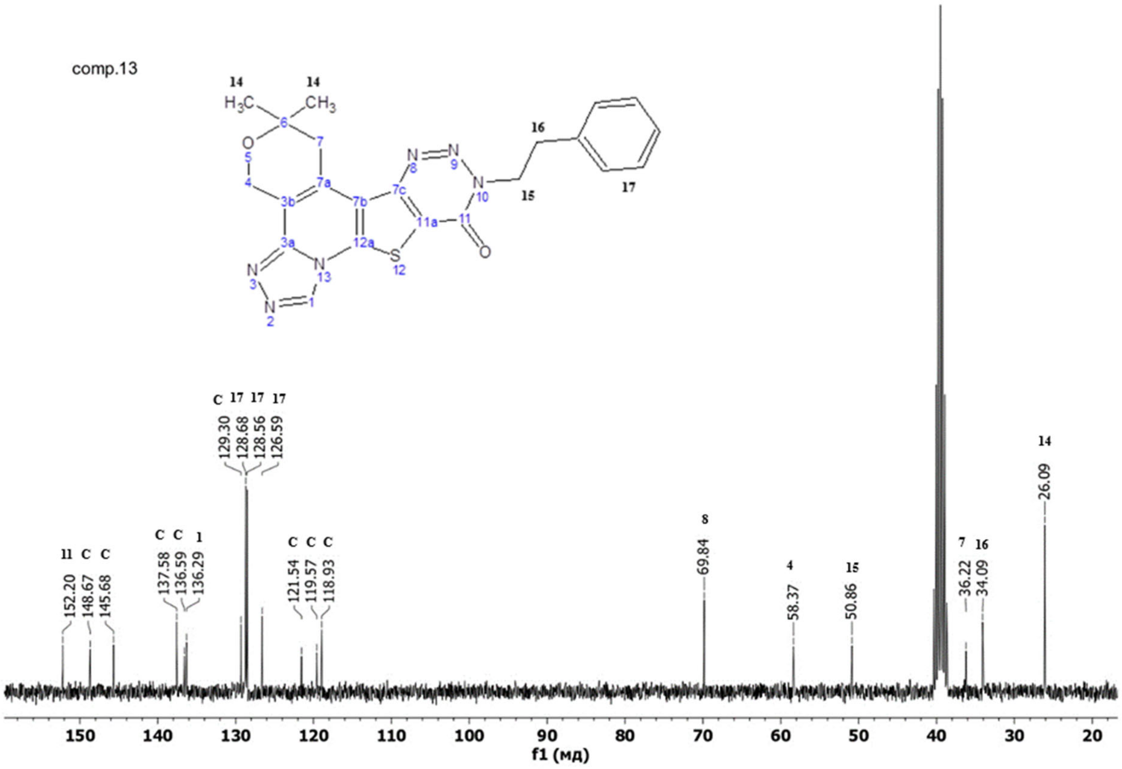

^{1}H and ^{13}C NMR spectrum of compound 14

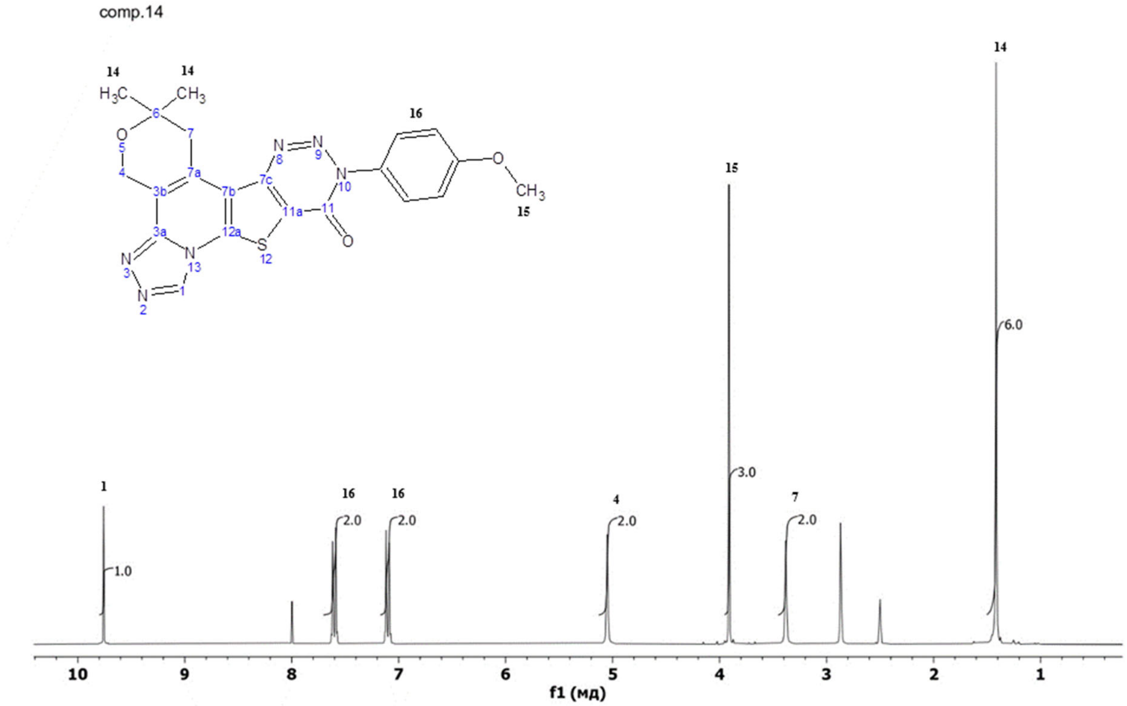

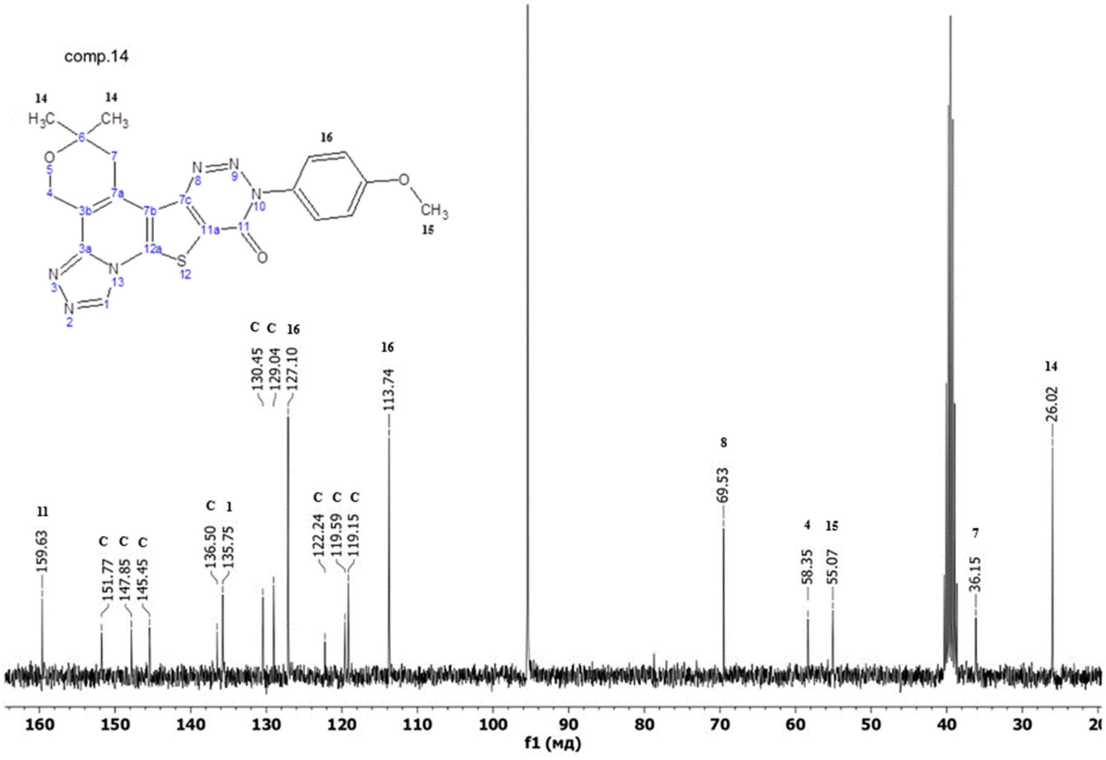

^{1}H and ^{13}C NMR spectrum of compound 15

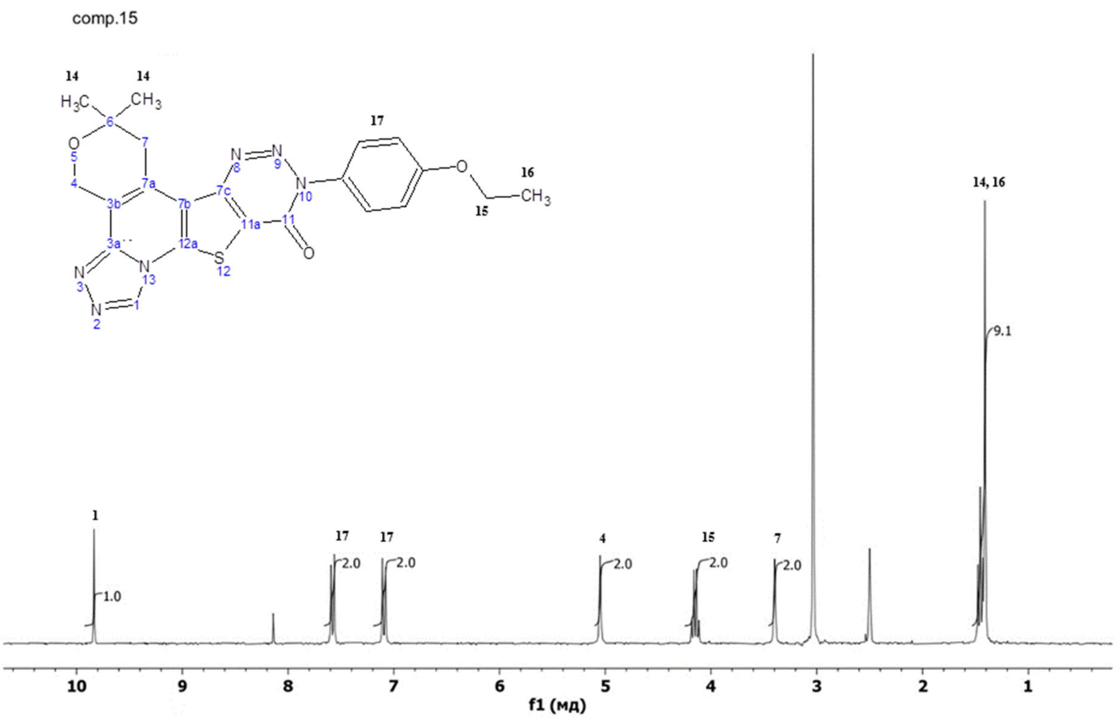

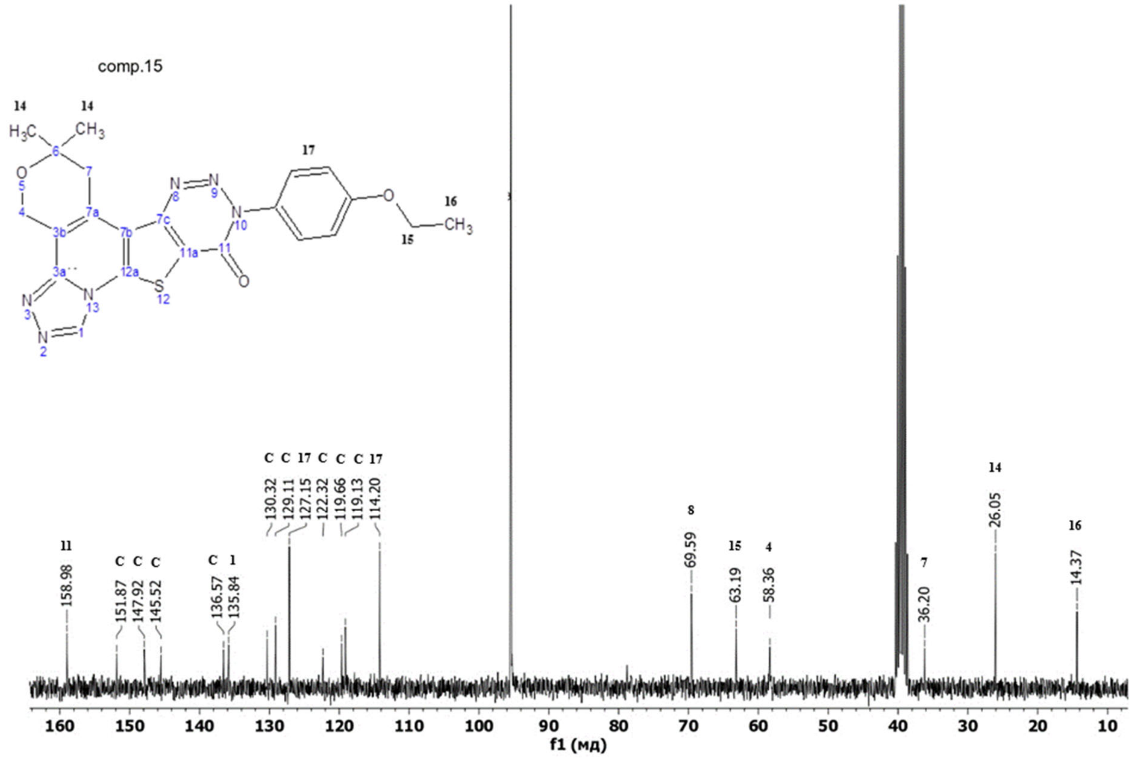

^{1}H and ^{13}C NMR spectrum of compound 16

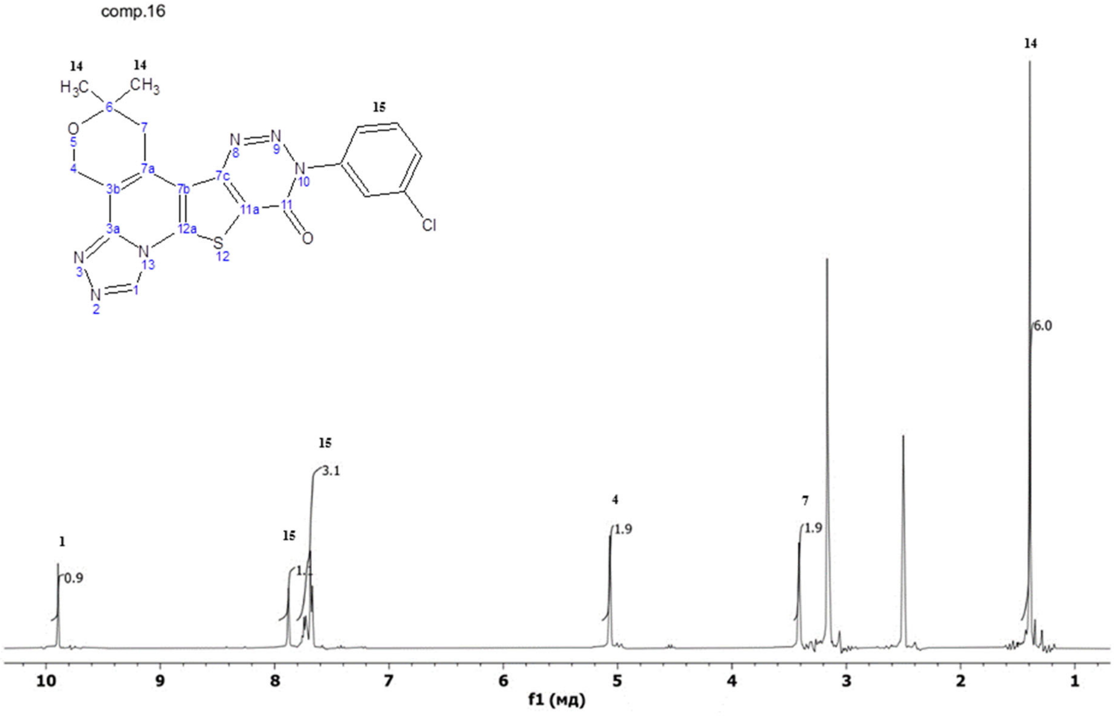

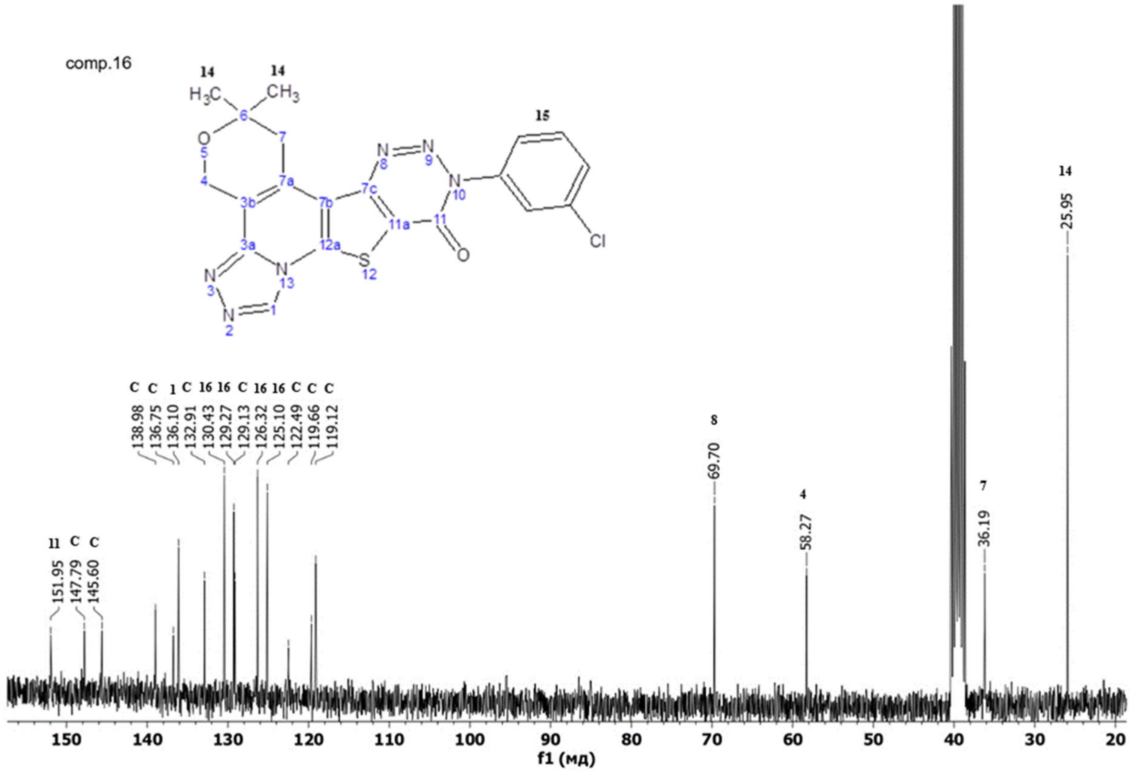

^{1}H and ^{13}C NMR spectrum of compound 17

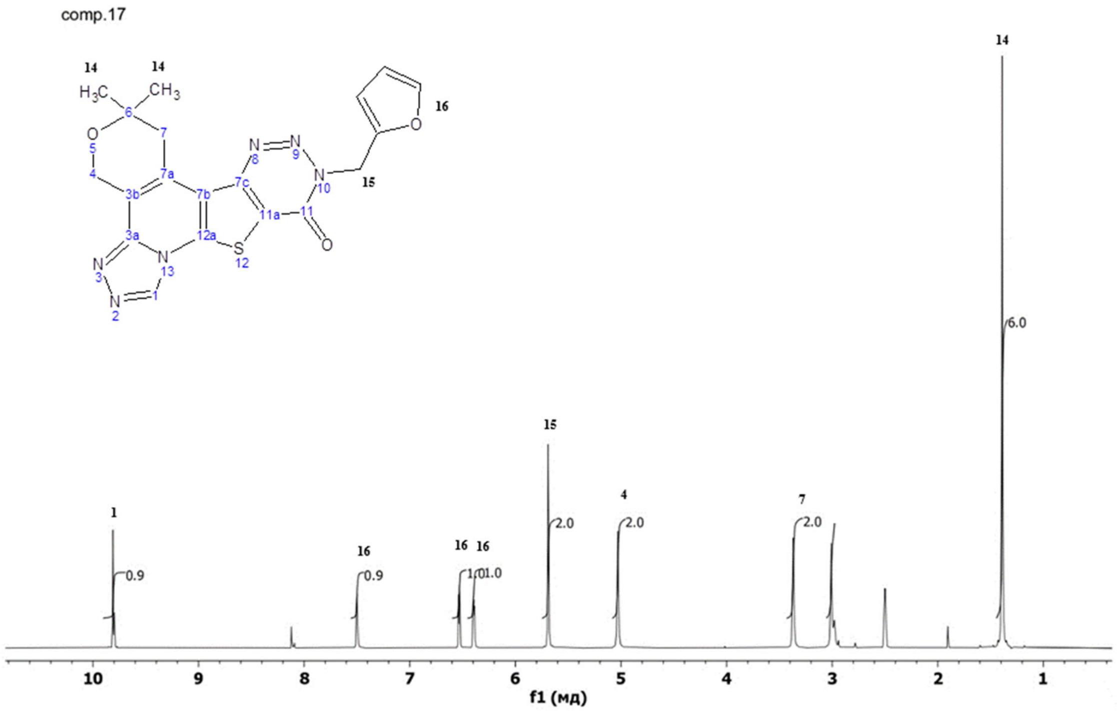

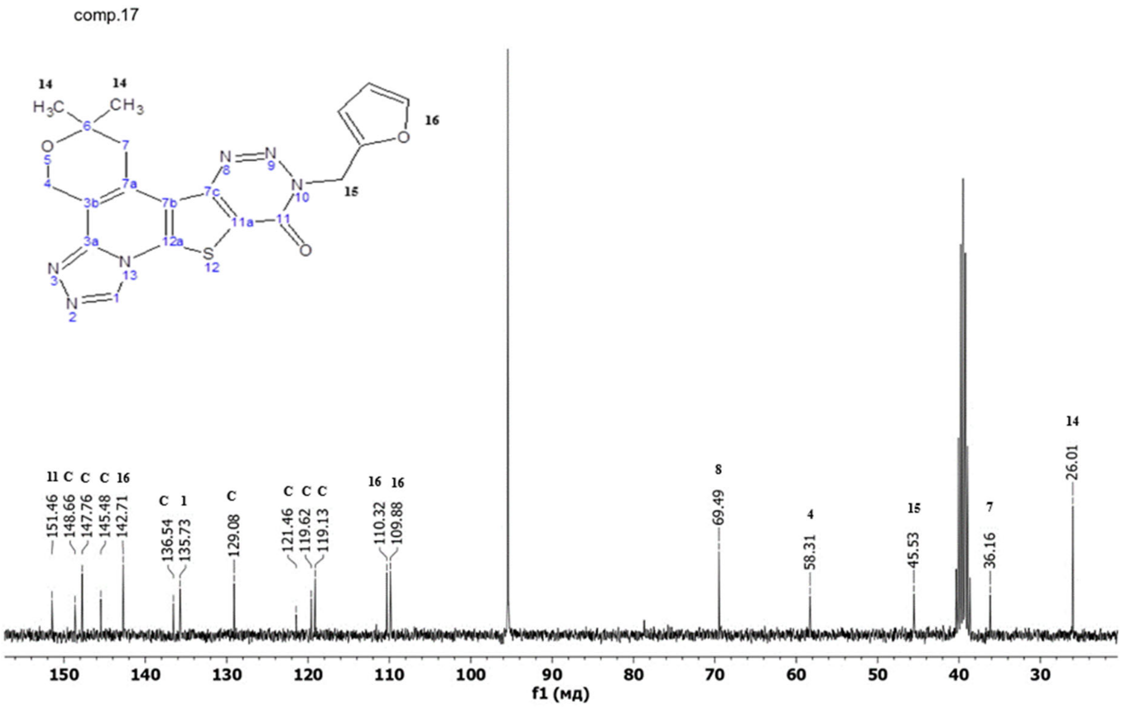

FTIR spectrum of Compound 5

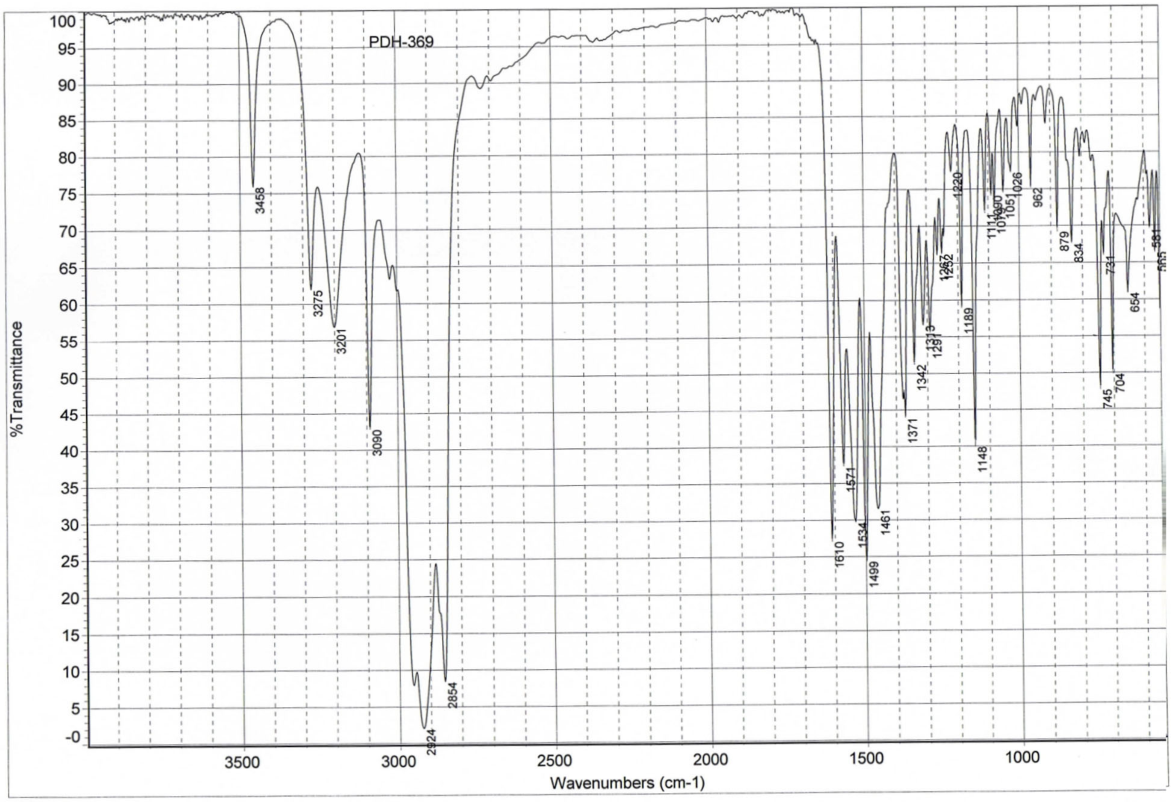

FTIR spectrum of Compound 6

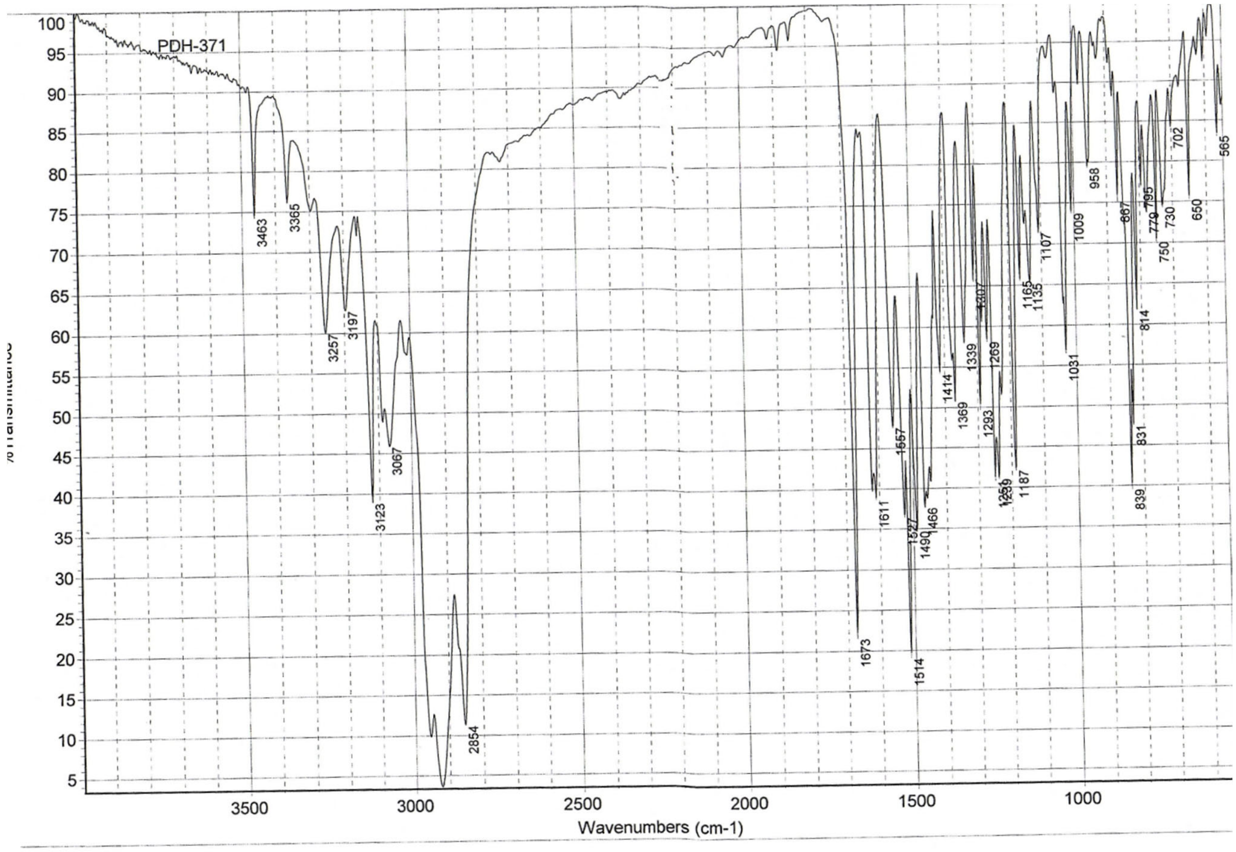

FTIR spectrum of Compound 7

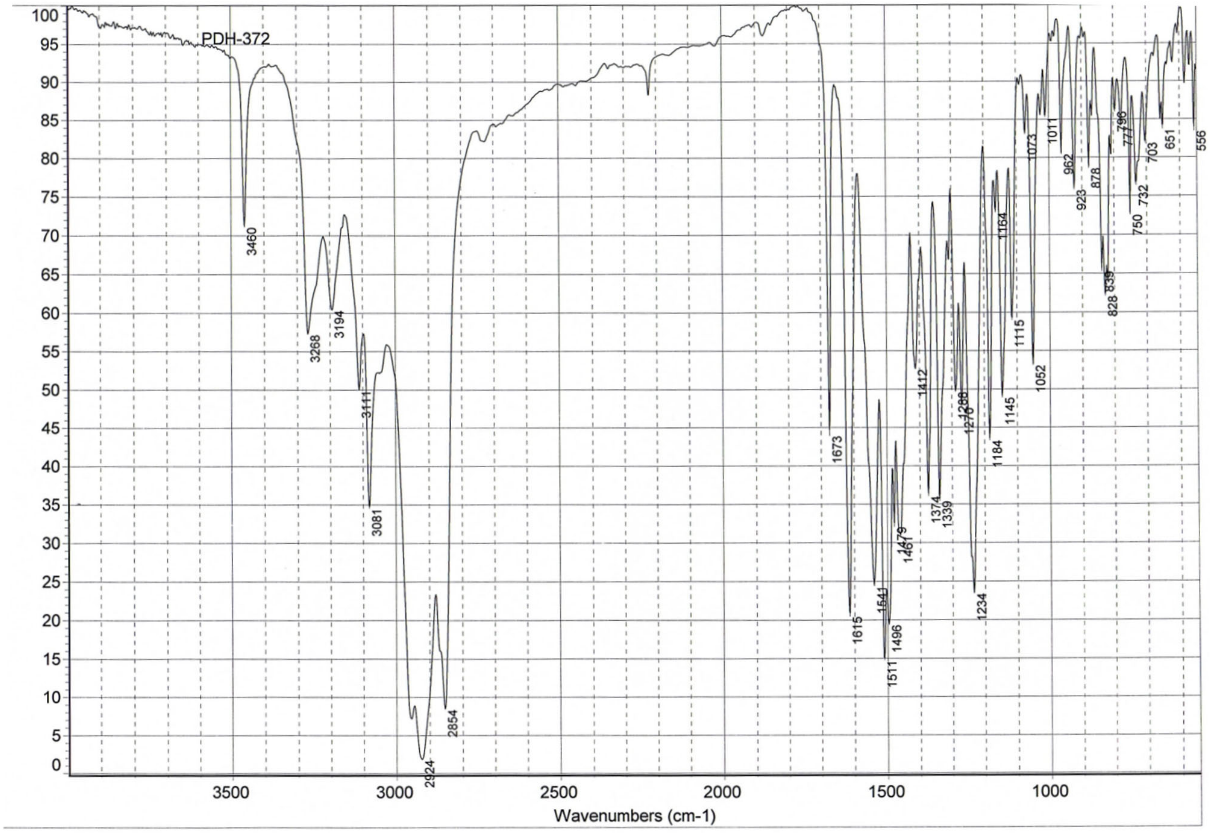

FTIR spectrum of Compound 8

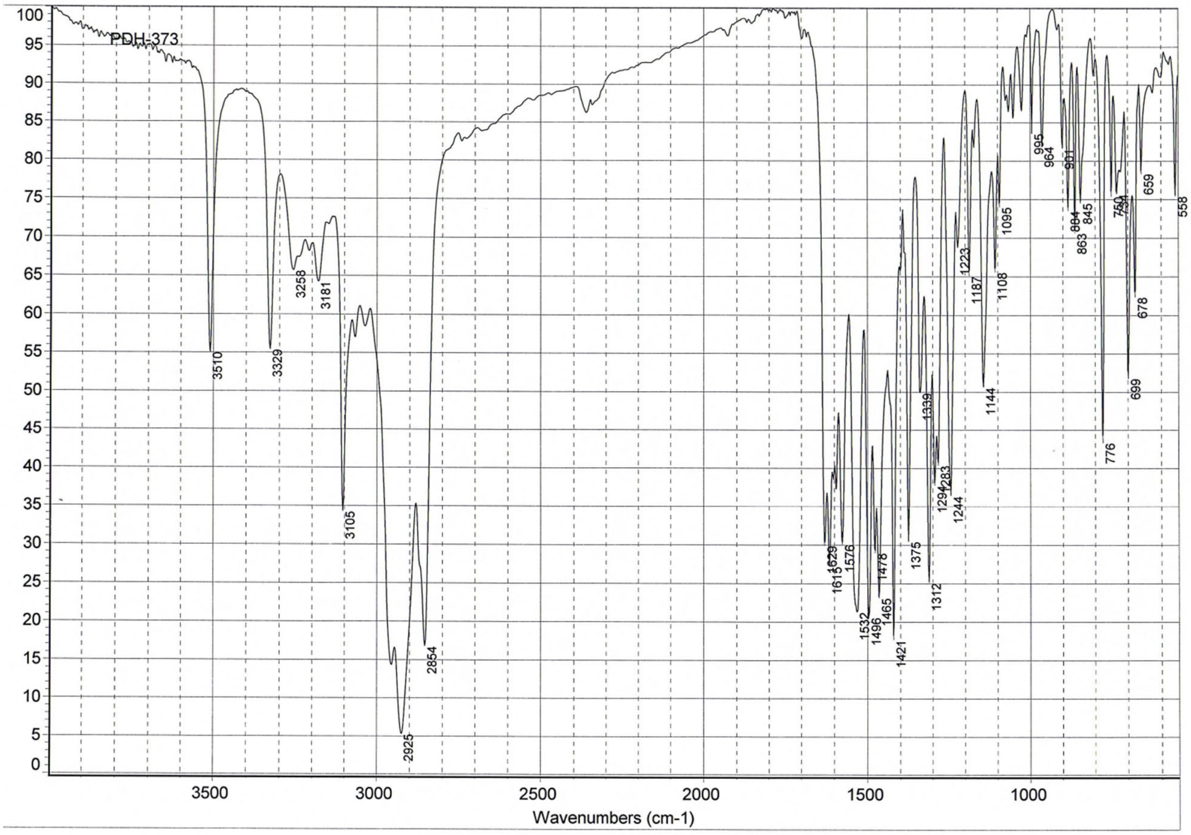

FTIR spectrum of Compound 9

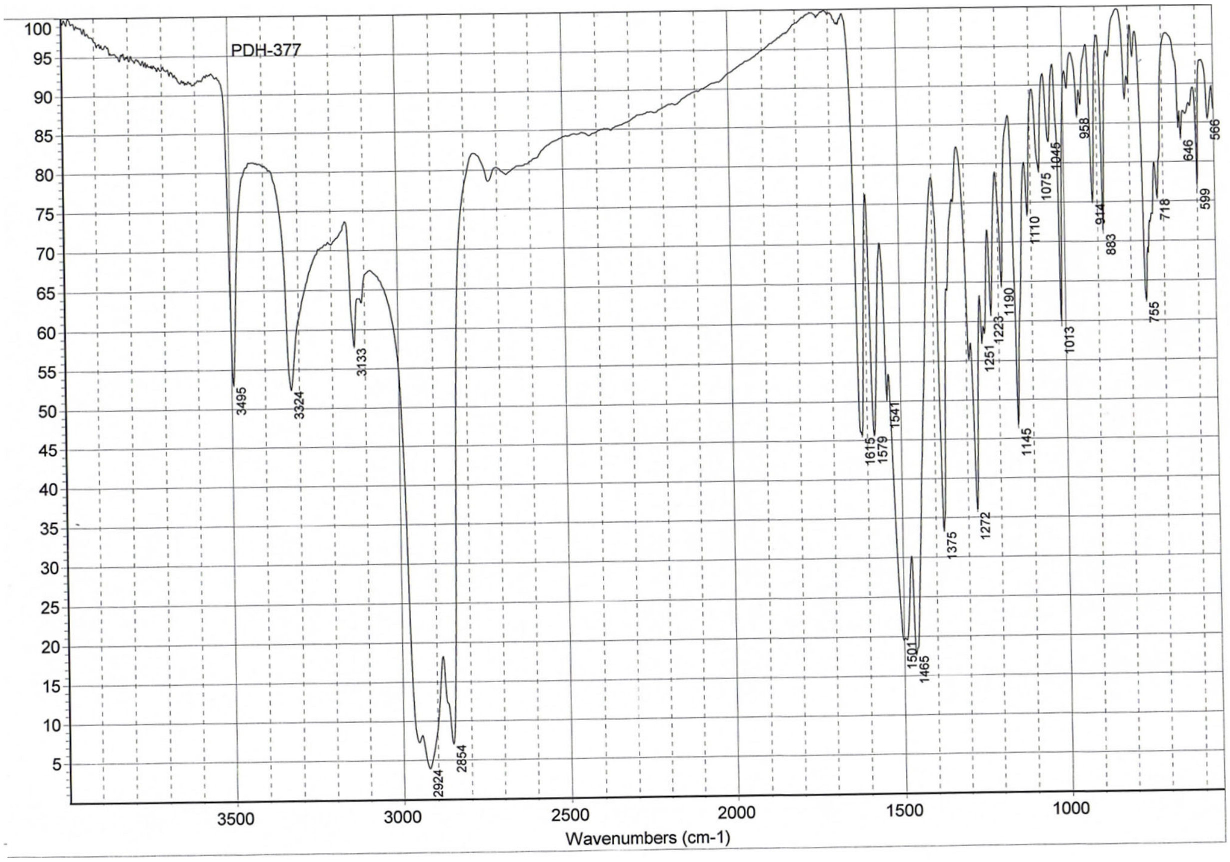

FTIR spectrum of Compound 10

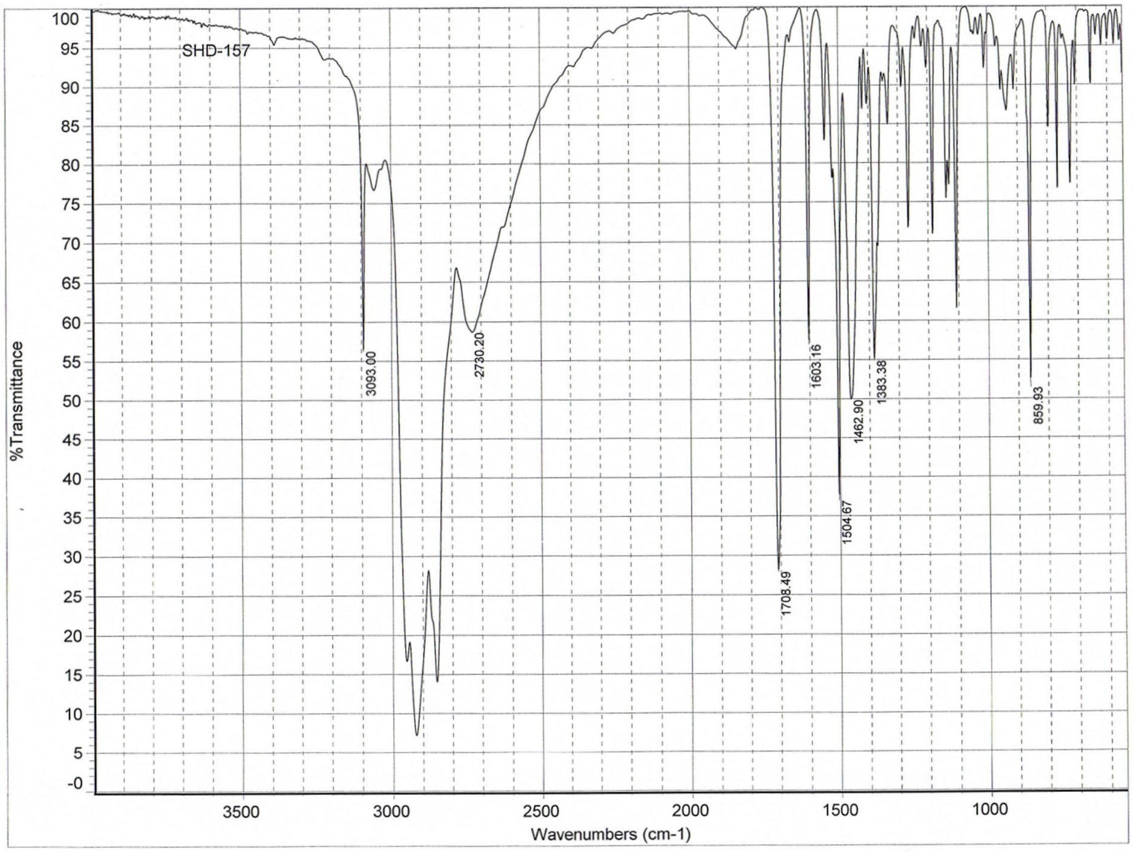

FTIR spectrum of Compound 11

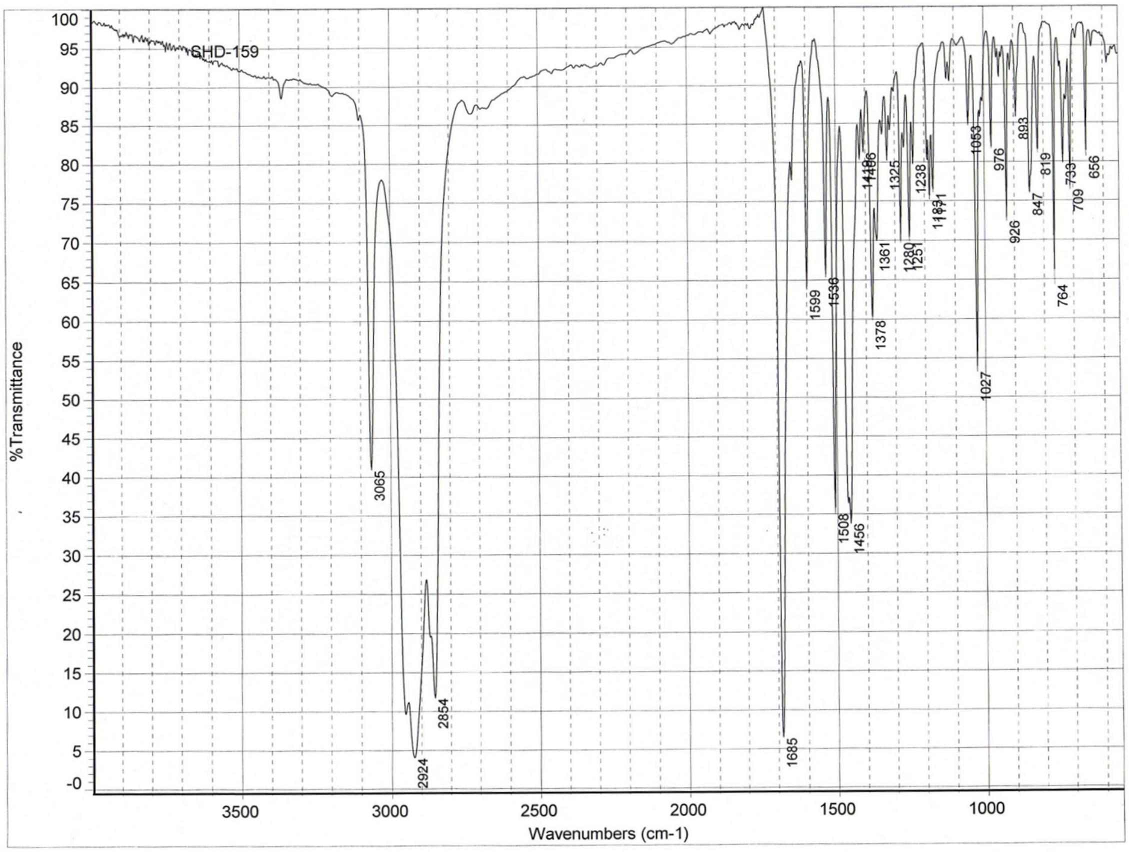

FTIR spectrum of Compound 12

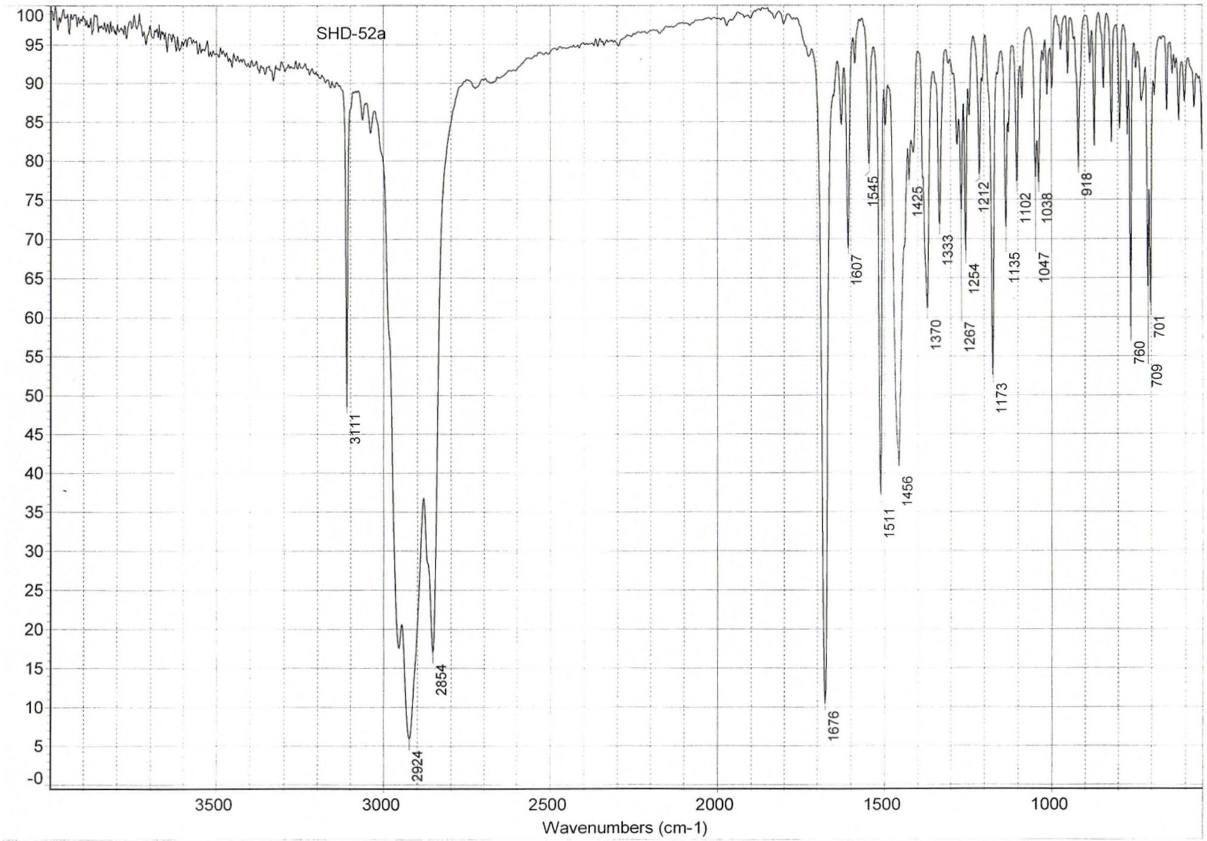

FTIR spectrum of Compound 13

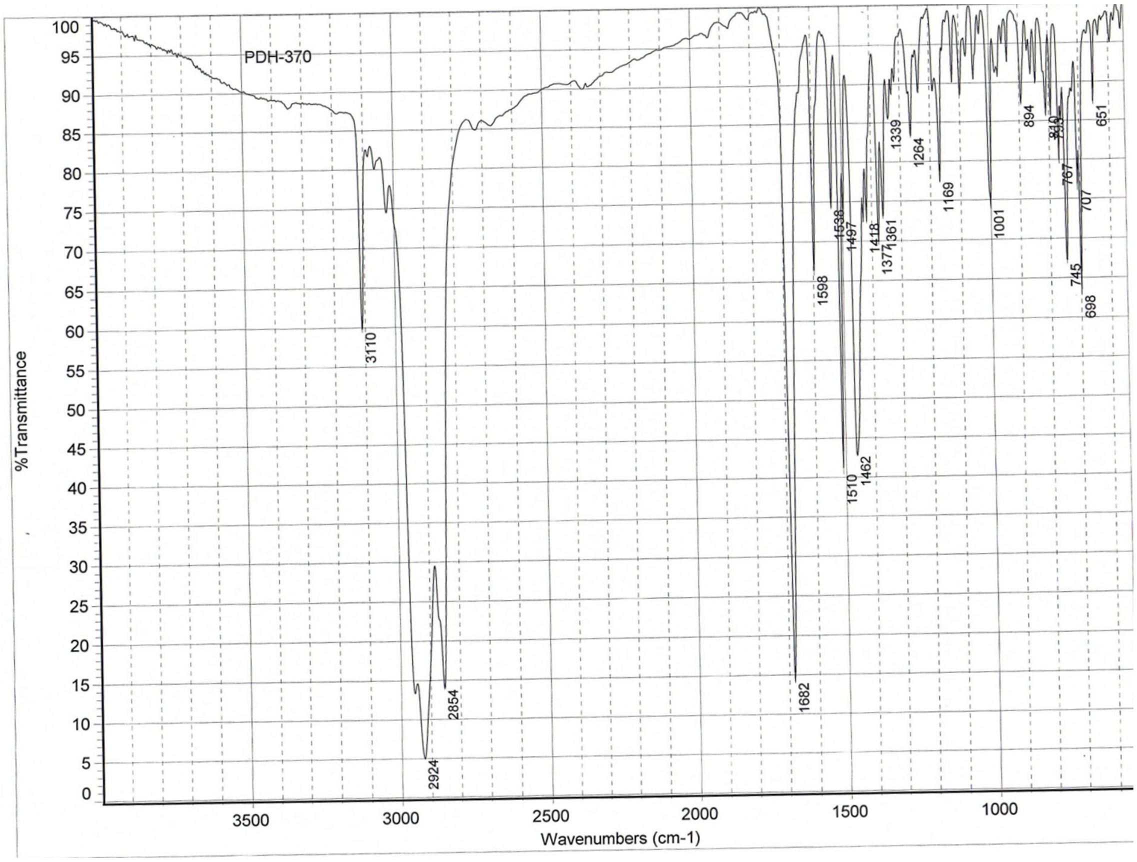

FTIR spectrum of Compound 14

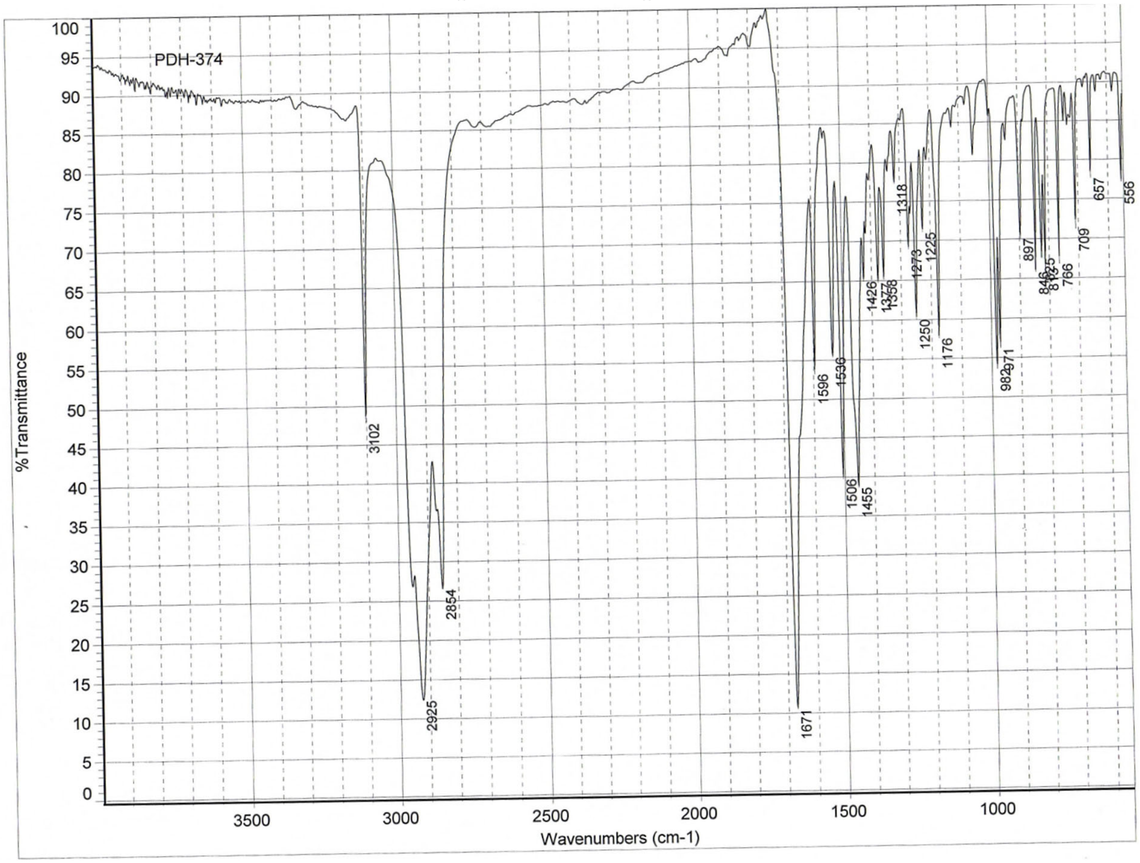

FTIR spectrum of Compound 15

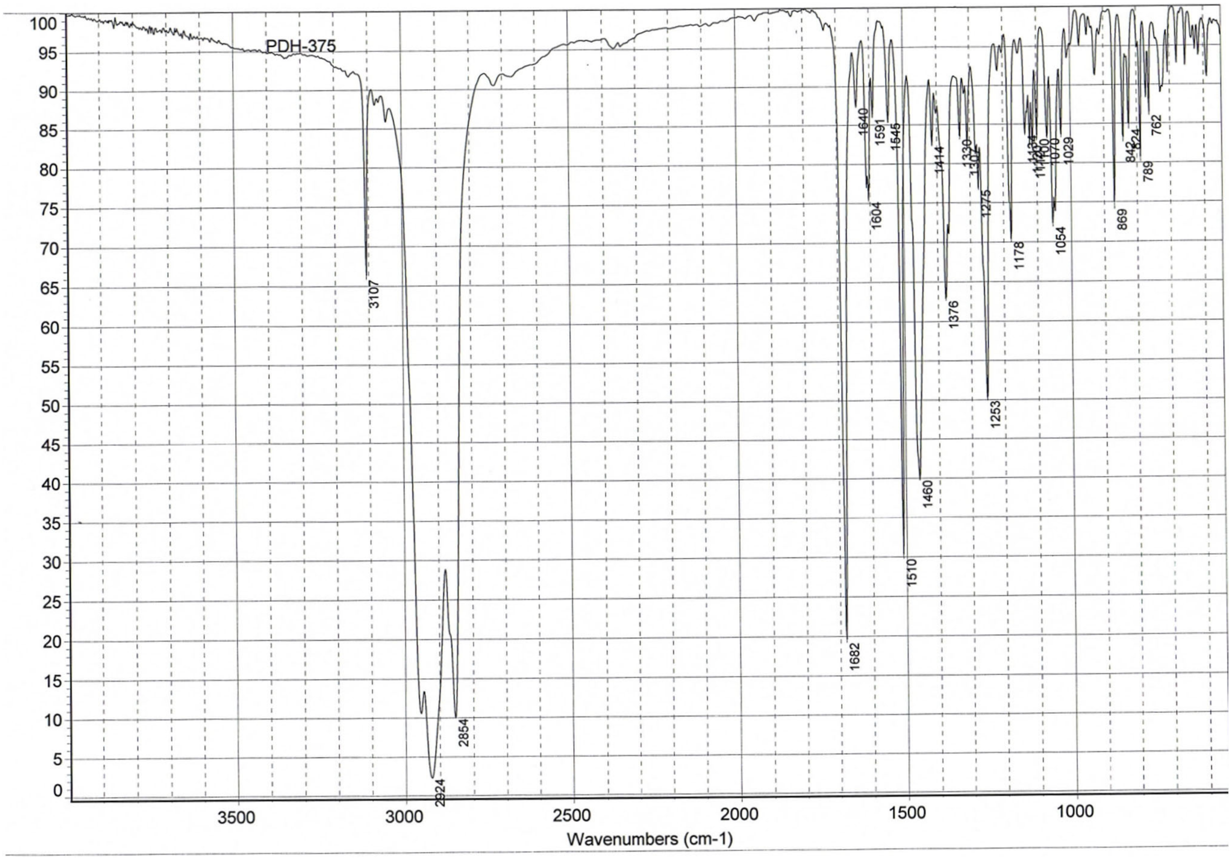

FTIR spectrum of Compound 16

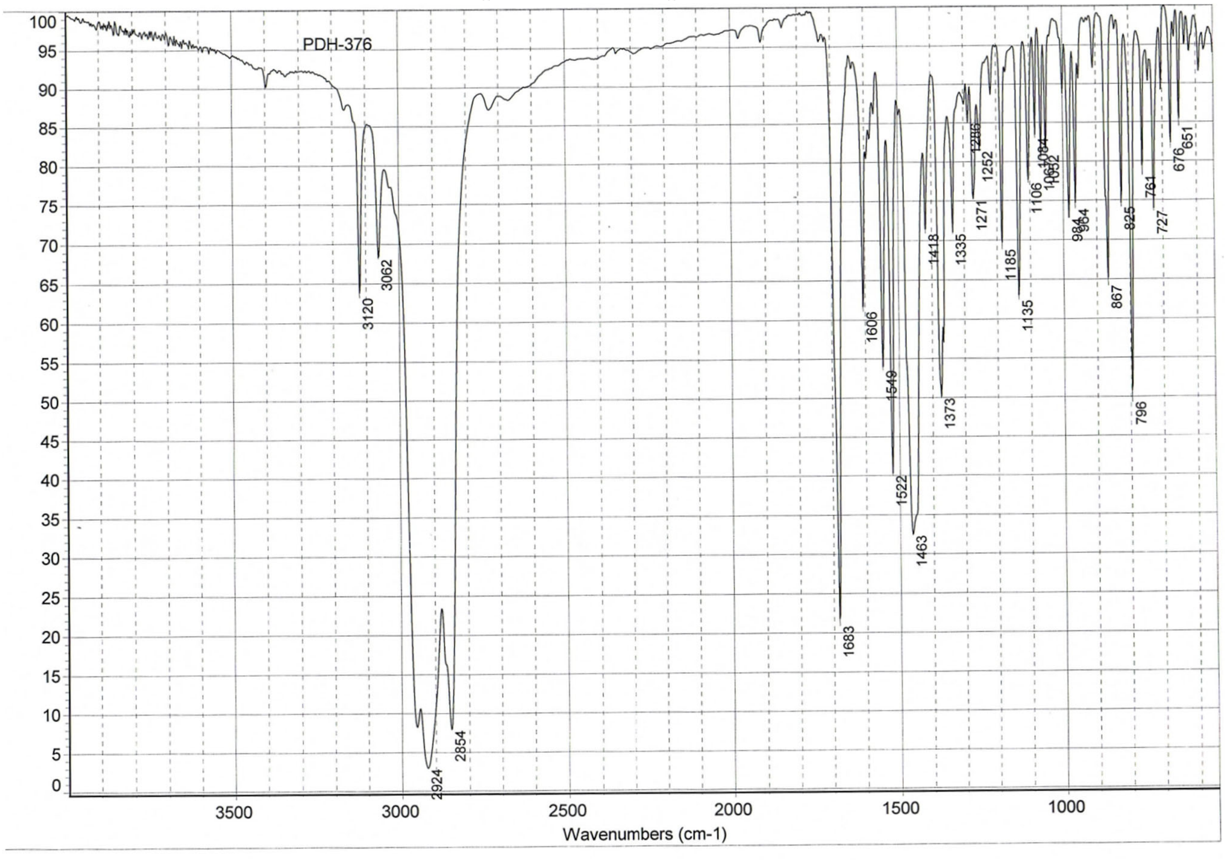

FTIR spectrum of Compound 17

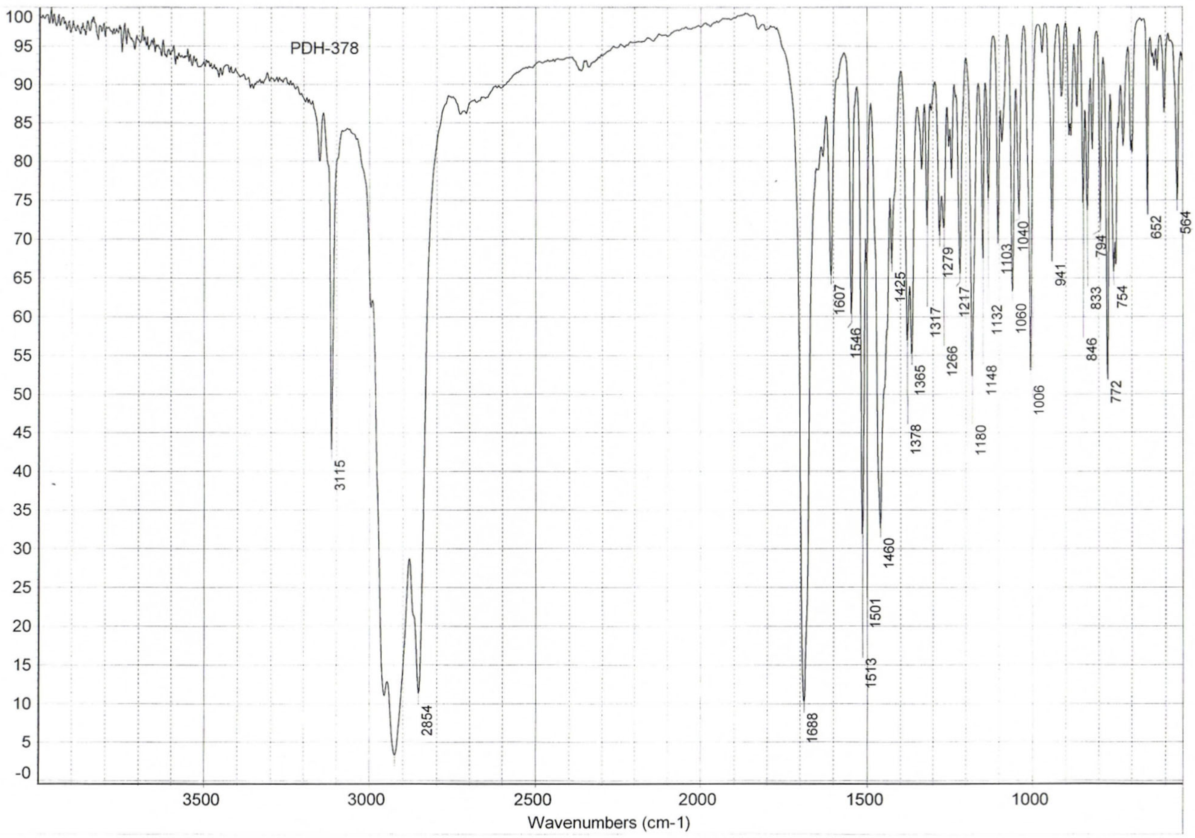

MASS spectrum of Compound 10

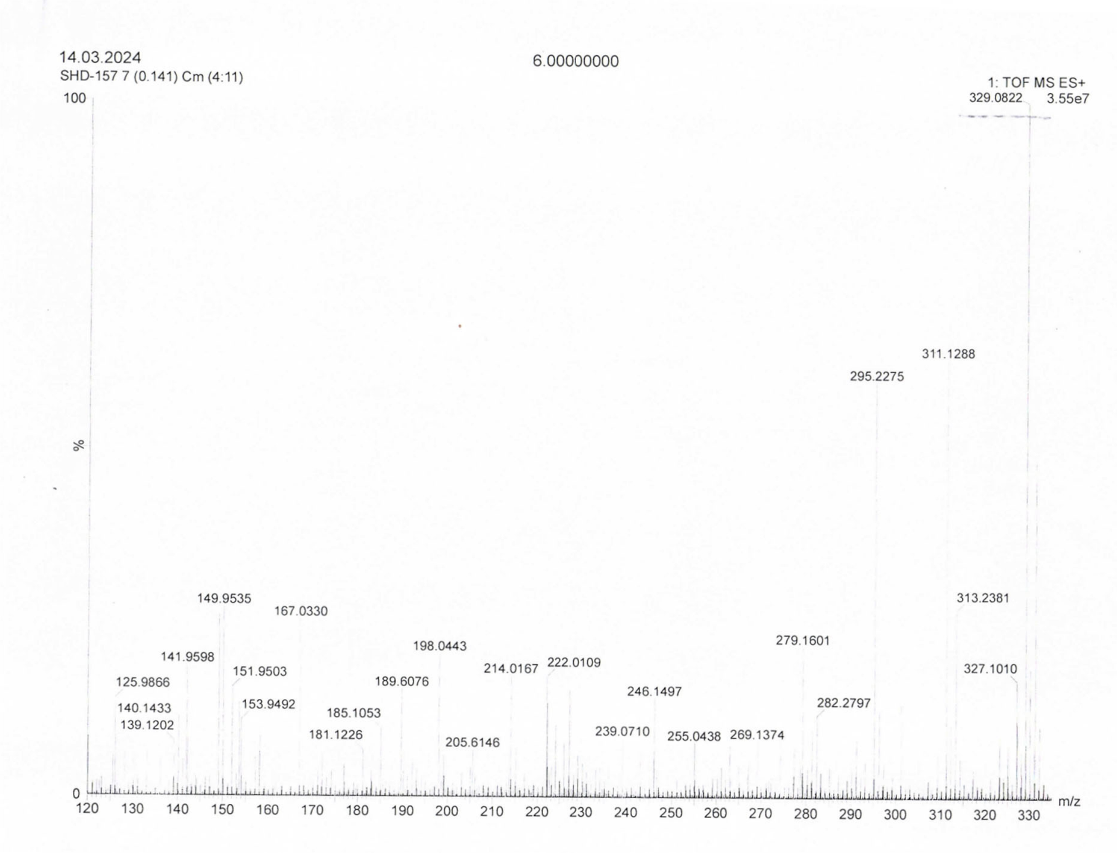

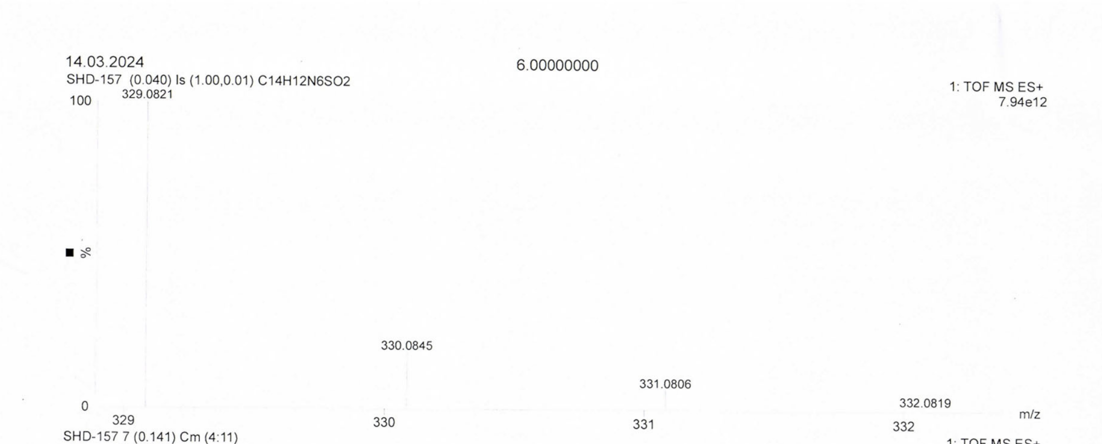

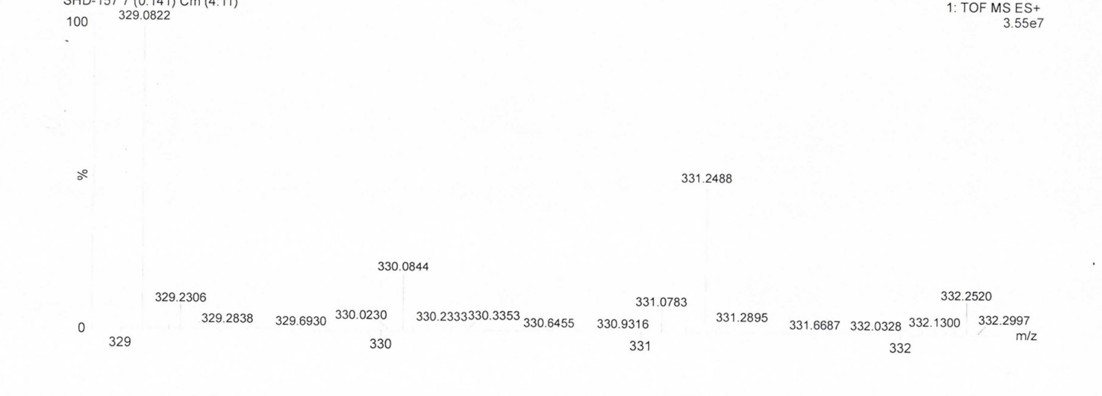

MASS spectrum of Compound 12

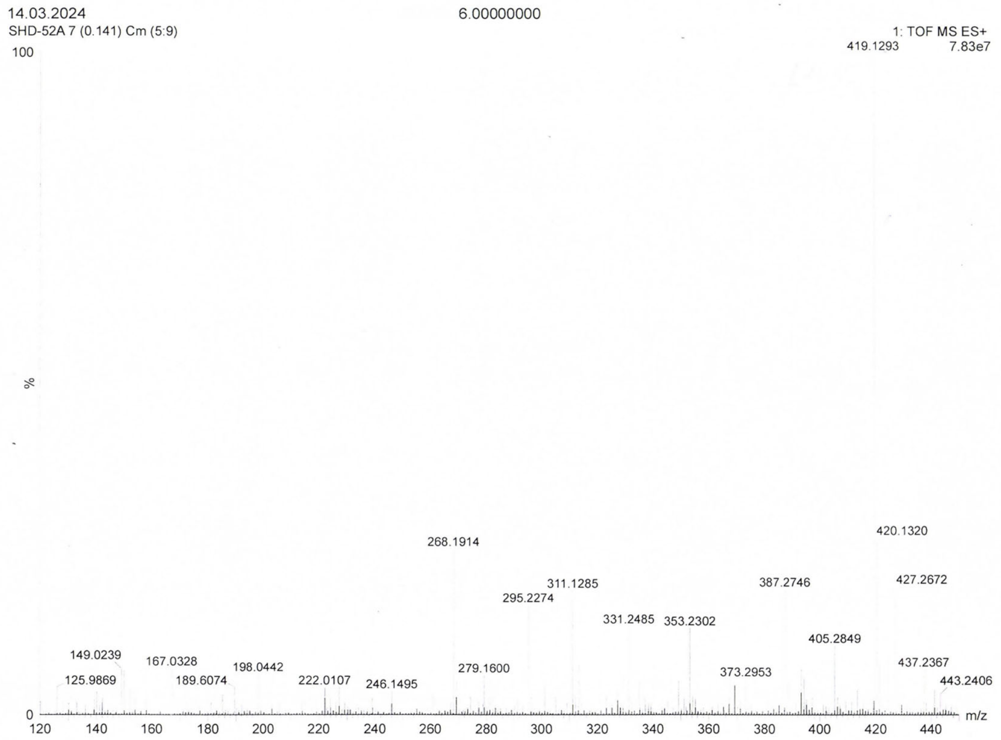

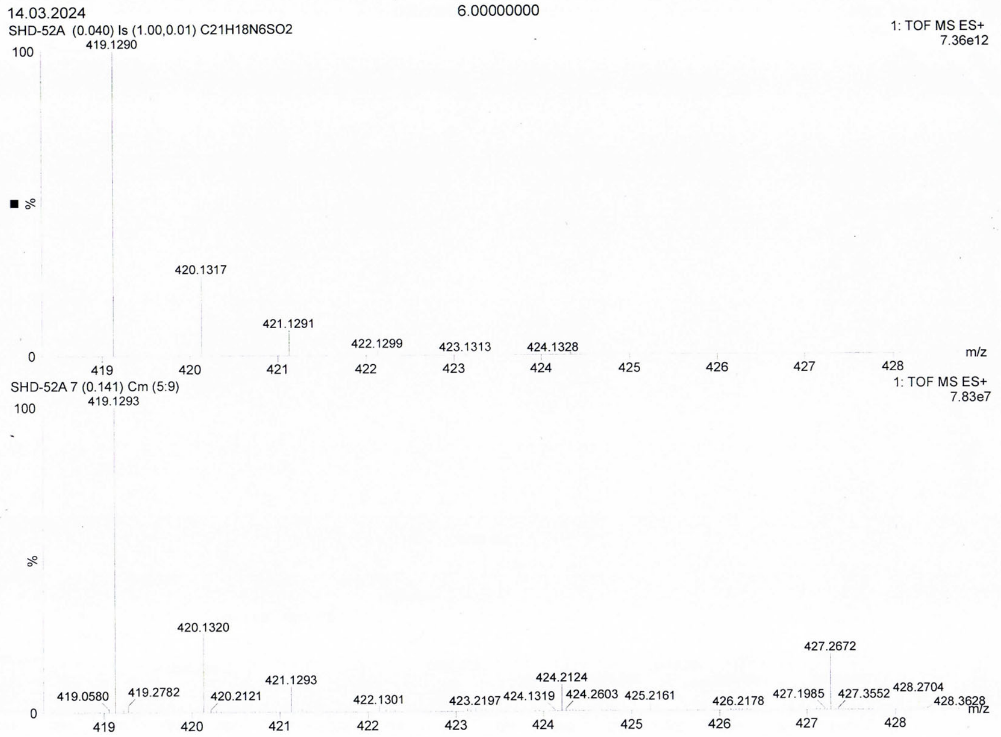

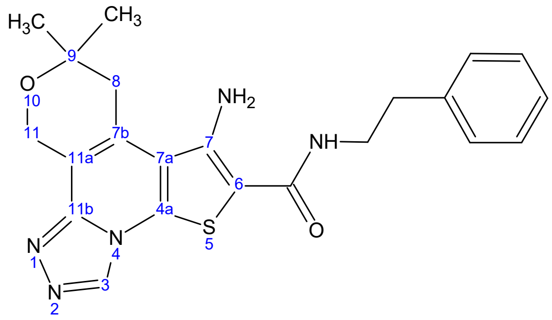

H3C
CH3
9
O
10
8
NH2
11
7b
11a
7a
7
NH
6
11b
4a
O
N
S
N
4
5
1
N
3
2
